# A Series of Novel 3D Coordination Polymers Based on the Quinoline-2,4-dicarboxylate Building Block and Lanthanide(III) Ions—Temperature Dependence Investigations

**DOI:** 10.3390/molecules28176360

**Published:** 2023-08-30

**Authors:** Dmytro Vlasyuk, Renata Łyszczek, Liliana Mazur, Agnieszka Pladzyk, Zbigniew Hnatejko, Przemysław Woźny

**Affiliations:** 1 Department of General and Coordination Chemistry and Crystallography, Faculty of Chemistry, Institute of Chemical Sciences, Maria Curie-Skłodowska University, M. C. Skłodowskiej Sq. 2, 20-031 Lublin, Poland; liliana.mazur@mail.umcs.pl; 2 Department of Inorganic Chemistry, Faculty of Chemistry, Gdańsk University of Technology, Narutowicza 11/12, 80-233 Gdańsk, Poland; 3 Department of Rare Earths, Faculty of Chemistry, Adam Mickiewicz University in Poznań, Uniwersytetu Poznańskiego 8, 61-614 Poznań, Poland; zbigniew.hnatejko@amu.edu.pl (Z.H.); przemyslaw.wozny@amu.edu.pl (P.W.)

**Keywords:** coordination polymers, lanthanide ions, luminescence, crystal structure, hydrothermal synthesis, coordination modes

## Abstract

A series of novel 3D coordination polymers [Ln_2_(Qdca)_3_(H_2_O)_x_]·yH_2_O (x = 3 or 4, y = 0–4) assembled from selected lanthanide ions (Ln(III) = Nd, Eu, Tb, and Er) and a non-explored quinoline-2,4-dicarboxylate building block (Qdca^2−^ = C_11_H_5_NO_4_^2−^) were prepared under hydrothermal conditions at temperatures of 100, 120, and 150 °C. Generally, an increase in synthesis temperature resulted in structural transformations and the formation of more hydrated compounds. The metal complexes were characterized by elemental analysis, single-crystal and powder X-ray diffraction methods, thermal analysis (TG-DSC), ATR/FTIR, UV/Vis, and luminescence spectroscopy. The structural variety of three-dimensional coordination polymers can be ascribed to the temperature effect, which enforces the diversity of quinoline-2,4-dicarboxylate ligand denticity and conformation. The Qdca^2−^ ligand only behaves as a bridging or bridging–chelating building block binding two to five metal centers with seven different coordination modes arising mainly from different carboxylate group coordination types. The presence of water molecules in the structures of complexes is crucial for their stability. The removal of both coordinated and non-coordinated water molecules leads to the disintegration and combustion of metal–organic frameworks to the appropriate lanthanide oxides. The luminescence features of complexes, quantum yield, and luminescent lifetimes were measured and analyzed. Only the Eu complexes show emission in the VIS region, whereas Nd and Er complexes emit in the NIR range. The luminescence properties of complexes were correlated with the crystal structures of the investigated complexes.

## 1. Introduction

Lanthanide complexes have been gaining more and more interest due to the possibility of creating various structures with good magnetic, luminescent, surface, catalytic, and thermal properties. These complexes can find many applications in different fields such as sorbents, storage materials, catalysts, sensors, optoelectronics, and medicine materials. Due to the high coordination number of lanthanide(III) ions and the nature of the ligands used in syntheses, they can create various discrete structures or coordination polymers (CPs) with different dimensionality and porosity (metal–organic frameworks, MOFs). The appropriate selection of metal ions and organic ligands allows the construction of optimized structures with further application possibilities [1,2,3,4,5,6,7]. The structural diversity of the coordination polymers also arises from specific conditions of the applied traditional solvothermal and non-solvothermal synthesis strategies. The influence of obvious factors such as temperature, pH, solvent, and synthesis time on the final architecture of the CP is widely known, but not easily explained [8,9,10,11].

The hydro/solvothermal methods widely used for the synthesis of CPs are characterized as highly efficient methods and allow obtaining compounds with a high degree of crystallinity. Predicting the crystal structure, and designing and controlling the CP topology is possible thanks to the detailed selection of hydro/solvothermal synthesis parameters [12]. The advantage of these hydro/solvothermal methods is the possibility of controlling the synthesis temperature (usually in the range of 100–200 °C), which is a very important factor in obtaining high-quality crystals. The synthesis temperature is also one of the key parameters influencing the structure and properties of CPs/MOFs; however, its impact is not completely recognized. In some reports, the authors claimed that the increase in temperature causes an increase in the dimensionality and coordination number of metal ions while the number of the coordinated solvent molecules decreased. Another group of researchers reported that the crystallinity of the metal complexes increases as the synthesis temperature decreases. Their results revealed that the density and dimensionality of CPs/MOFs increase with increasing temperature [12,13,14]. The impact of temperature on the crystal structures of metal complexes is reflected in the coordination environments of metal centers, as well as coordination modes of ligands [15,16,17,18,19,20,21,22]. In hydro/solvothermal synthesis, the temperature change causes the formation of different autogenous pressure, which influences the crystallization process of the synthesized products, depending on the characteristics of the substrates used. 

It is well known that N-heterocyclic polycarboxylic acids are excellent candidates for the formation of metal complexes due to their different coordination modes. Yet, the properties of quinoline-2,4-dicarboxylic acid as a multidentate N,O-donor ligand are not really well recognized in the construction of discrete metal complexes and coordination polymers. To best of our knowledge, only a few examples of crystal structures of metal complexes are known that contain analogous of quinoline-2,4-dicarboxylic acid [23,24,25]. Most of the reported complexes were built up from transition metals and N-donor auxiliary ligands such as 1,10-phenanthroline; 4,4’-bipyridine, or 2,2’-bipyridine [25,26,27,28,29,30,31,32]. The main aim of this contribution to the topic highlighted above was to determine the impact of temperature effect on the self-assembly coordination polymers constructed from selected lanthanide(III) ions (Ln = Nd, Eu, Tb, Er) and quinoline-2,4-dicarboxylic acid (H_2_Qdca) as a non-explored organic bridging–chelating building block. The second goal of our investigation was to study the luminescence properties of the obtained compounds due to their further application as active luminescent components in polymeric hybrid materials. Lanthanide coordination entities are attractive because of their interesting physicochemical features and numerous applications as so-called new materials. The reason for this is that the luminescence of the lanthanides is unique with their sharp emission bands and higher lifetimes of the excited states [33,34,35,36,37] in the visible (Sm, Eu, and Tb) and NIR region (Pr, Nd, Ho, Er, and Yb) [38,39,40]. In particular, the luminescence of lanthanides in the NIR range is very attractive because of their potential applications in the medical field as luminescent probes, in telecommunication, and in photonic devices such as light-emitting diodes [34,35,36,37,41,42,43,44].

As a result of our work, four groups of isostructural 3D coordination polymers were synthesized under hydrothermal conditions at different temperatures (100 °C, 120 °C. and 150 °C). Single-crystal and powder X-ray diffraction methods were applied for structural investigations of the obtained lanthanide complexes. Crystal structures and diverse conformation and coordination modes of the quinoline-2,4-dicarboxylate linker were analyzed in detail, along with a discussion on *crystal-to-crystal* transformations. Thermal (TG-DSC) and spectroscopic (ATR-FTIR, UV/Vis, and luminescence spectroscopy) characterizations of the investigated lanthanide complexes are presented in detail below.

## 2. Results and Discussion

**Figure 1:**
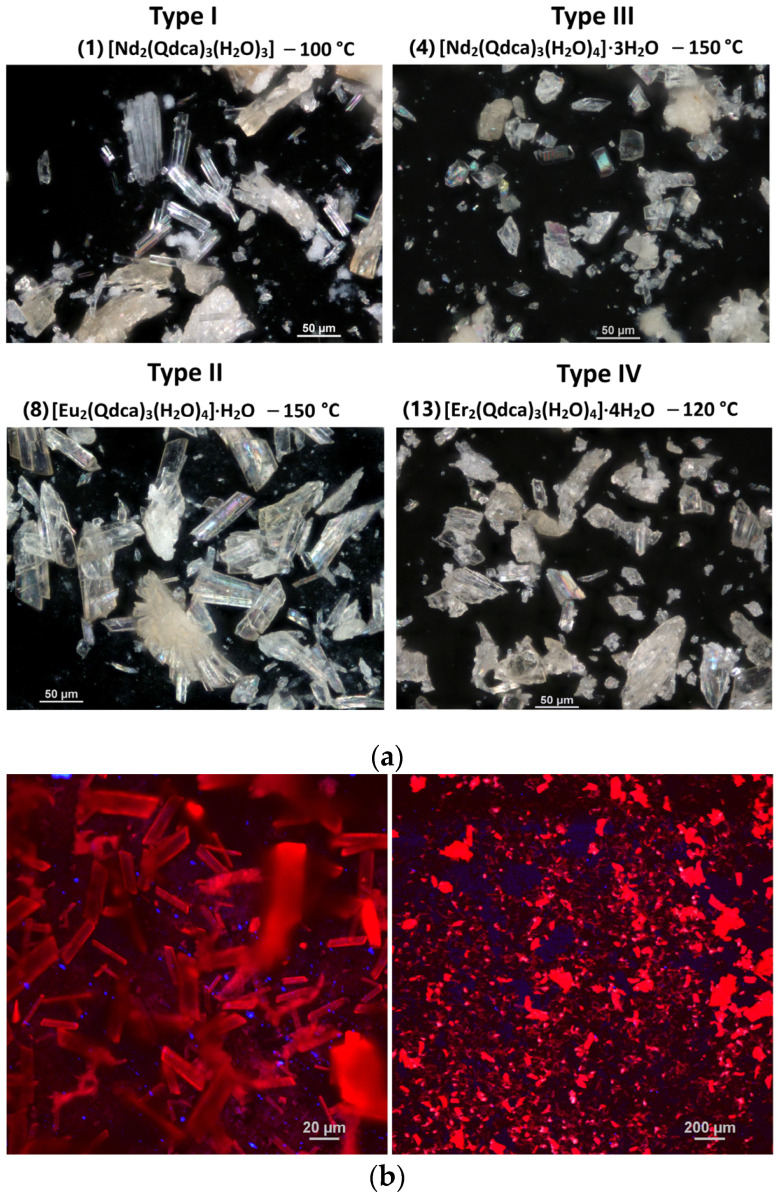
(**a**) Optical microscope images of crystals from four different groups; (**b**) images under a confocal microscope of europium(III) sample obtained at 100 °C (λ_ex_ = 420 nm).

The coordination polymers of selected lanthanide ions, i.e., Nd(III), Eu(III), Tb(III), and Er(III), with the quinoline-2,4-dicarboxylic acid (H_2_Qdca) as a novel building block were prepared via a temperature-dependent hydrothermal method at 100, 120, and 150 °C (Table S1). All as-synthesized coordination polymers at 100 and 120 °C, along with those of Eu(III) and Tb(III) formulated at 150 °C, were obtained as single crystals (Figure 1). Only compounds of Nd(III) and Er(III) ions synthesized at 150 °C were amorphous, and their recrystallization from water resulted in monocrystal forms. The single-crystal X-ray measurements were carried out for all isolated crystalline forms of obtained compounds that allowed establishing the unit cell parameters. Unfortunately, despite multiple repetitions of diffraction measurements, it was not possible to obtain data of appropriate quality that would enable the solution and refinement of the crystal structures of all the measured crystals. All crystallographically investigated samples are indicated by numbers **1**–**14**.

The crystalline compounds of the general formula of [Ln_2_(Qdca)_3_(H_2_O)_x_]·yH_2_O, where (Ln(III) = Nd, Eu, Tb and Er; Qdca^2−^ = C_11_H_5_NO_4_^2−^; x = 3 or 4 and y = 0–4) could be divided into five isostructural (**I**–**V**) groups on the basis of crystal data obtained from both the single-crystal and the powder X-ray diffraction measurements. Taking into account the changes in unit cell parameters of isostructural complexes (Table 1), a gradual decrease in parameters *a*, *b*, and *c* with increasing atomic number of lanthanides was observed due to lanthanide contraction [45]. It is noteworthy to mention that, during hydrothermal synthesis at 100 °C, two crystal phases of neodymium(III) and europium(III) quinoline-2,4-dicarboxylates were formed.

Most of the studied coordination polymers (except group **I**) crystallized in the triclinic *P*-1 space group, and they differed in the number of inner and outer coordination water molecules, as well as in the coordination modes and conformation of Qdca^2−^ anion. The crystal structure of type **I** constituted only the neodymium(III) complex [Nd_2_(Qdca)_3_(H_2_O)_3_] (sample **1**) obtained at 100 °C (the first crystal phase). The second group of compounds (type **II**) contained isostructural [Nd_2_(Qdca)_3_(H_2_O)_4_]·H_2_O observed as the second phase at 100 °C (sample **2**) and only phase at 120 °C (**3**), [Eu_2_(Qdca)_3_(H_2_O)_4_]·H_2_O formed at 100 °C (sample **6**), 120 °C (sample **7**), and 150 °C (sample **8**), and [Tb_2_(Qdca)_3_(H_2_O)_4_]·H_2_O (sample **9**) formed at 100 °C. The third group of isostructural metal complexes (type **III**) represented by the general formula [Ln_2_(Qdca)_3_(H_2_O)_4_]·3H_2_O formed lanthanide ions neodymium(III) at 150 °C (sample **4**), europium(III) second phase at 100 °C (sample **5**), and terbium(III) at 120 °C (sample **10**) and 150 °C (sample **11**). Erbium(III) coordination polymers of the formula [Er_2_(Qdca)_3_(H_2_O)_4_]·4H_2_O obtained at 100 °C (sample **12**) and 120 °C (sample **13**) represented the fourth type (**IV**) of crystal structure. Unfortunately, the quality of single crystals of recrystallized erbium(III) complex obtained at 150 °C (sample **14**) only allowed establishing its unit cell parameters. These data may point to the formation of a fifth undefined type of crystal structure (**V**).

### 2.1. Structural Characterization

The best diffraction data were obtained for crystals selected from samples **1**, **4**, **8**, **9**, and **13** (Table 2), while, for the description of crystal structures representing the distinguished series (**I–IV**) of isostructural coordination polymers, the crystal structures of **1** (type **I**), **8** (type **II**), **4** (type **III**), and **13** (type **IV**) were selected.

**Figure 2:**
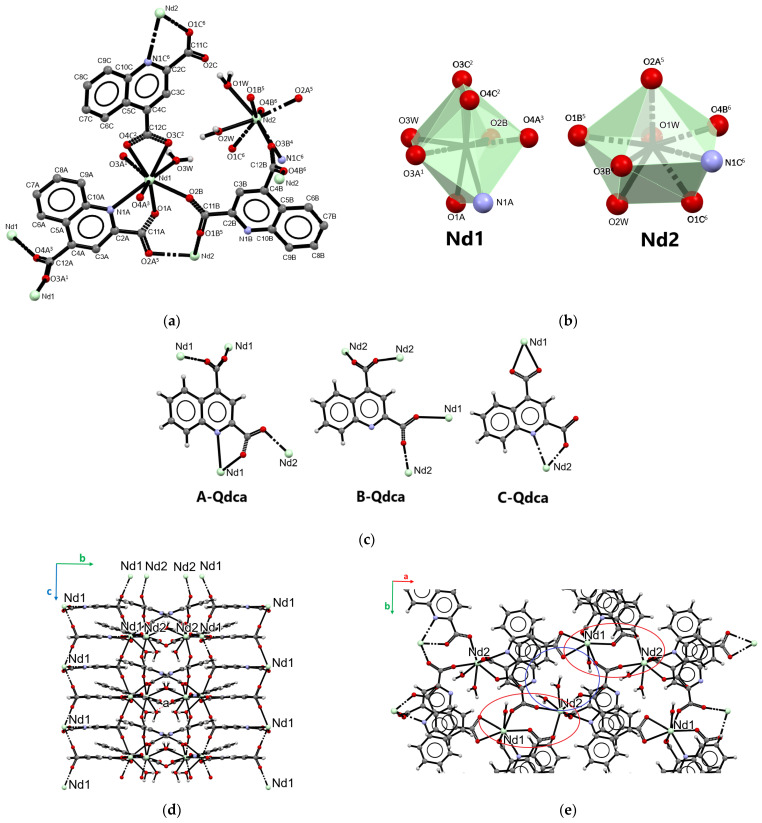
Part of the crystal structure of [Nd_2_(Qdca)_3_(H_2_O)_3_] (type **I**): (**a**) central metal environments in [Nd_2_(Qdca)_3_(H_2_O)_3_]; (**b**) coordination polyhedra of Nd atoms; (**c**) coordination modes of the Qdca^2−^ anions; (**d**) packing view along the *a* axis; and (**e**) packing view along the *c* axis.

The neodymium(III) coordination polymer [Nd_2_(Qdca)_3_(H_2_O)_3_] (sample **1**) formed at 100 °C, as the only representative of type **I**, crystallized in the orthorhombic *Pna*2_1_ space group. The structure of the complex is displayed in Figure 2, while the selected bond lengths and angles are listed in Tables S2, S7 and S12. The asymmetric unit contained two neodymium(III) ions, three quinoline-2,4-dicarboxylate ligands, and three coordinated water molecules (Figure 2a). The complex had the form of a three-dimensional coordination polymer with two crystallographically independent central atoms. Both neodymium atoms were eight-coordinated. The coordination of Nd1 atom occurred through six carboxylate oxygen atoms from five Qdca^2−^ anions, one oxygen atom from the aqua ligand, and one nitrogen atom from the Qdca^2−^ anion. The coordination sphere of the Nd2 atom consisted of five carboxylate oxygen atoms from five Qdca^2−^ anions, two oxygen aqua ligands, and one nitrogen atom (Figure 2b). The Nd–O_carb_ bond distances were in the range 2.355(5)–2.581(5) Å. The Nd–O_w_ bond distances ranged from 2.433(5) to 2.449(6) Å, while the Nd–N bond lengths varied in the range of 2.664(4)–2.888(4) Å (Table S2). The remaining bond lengths and angles in quionoline-2,4-dicarboxylate ligands were within their normal ranges (Tables S2 and S7).

Three different coordination modes and conformations of Qdca^2−^ anion could be distinguished in the structure of [Nd_2_(Qdca)_3_(H_2_O)_3_] (Figure 2c) (Tables S12, S22 and S23). The molecule A-Qdca acted as a pentadentate bridging–chelating ligand with both bidentate-bridging (μ_2_-η^1^:η^1^) carboxylate groups. The adjacent carboxylate oxygen atom O1A, along with the nitrogen atom of A-Qdca ligand, chelated the Nd1 atom to form a five-membered ring. The non-planar *syn*–*anti* carboxylate group in position 2 was only slightly rotated from the plane of the quinoline ring, as indicated by the value of the dihedral angle equals to 12.9° (throughout the work, reference was made to the dihedral angle between the carboxylate group and the pyridine plane of the quinoline). The non-planar *anti*–*anti* carboxylate group at position 4 was almost perpendicular to the plane of an almost plane quinoline ring, as shown by 88.37°. The B-Qdca ligand behaved as tetradentate ligand coordinating four neodymium atoms through both bidentate-bridging carboxylate groups (μ_2_-η^1^:η^1^). The COO group in position 2 was rotated from plane of the quinoline ring by 6.11°, while rotation of the second carboxylate group was greater, being 62.61°. The carboxylate group in position 2 coordinated Nd atoms in a non-planar *syn*–*syn* mode, while the COO group in position 4 had non-planar *anti*–*anti* mode. The C-Qdca ligand could be regarded as tetradentate bridging–chelating agent binding two neodymium atoms. The carboxylate group in position 2 showed a monodentate mode and, along with the nitrogen quinoline atom, chelated the neodymium atom into the five-membered ring. This group was rotated by 6.96° from the quinoline ring. In comparison to the remaining Qdca^2−^ anion, the carboxylate group in position 4 was only rotated by 24.96° from the plane of the heterocyclic ring.

The compound [Nd_2_(Qdca)_3_(H_2_O)_3_] had the form of a three-dimensional coordination polymer. Its crystal packing viewed along the *a* axis (Figure 2d) showed characteristic structural motifs. The Nd1 atoms were connected through single carboxylate bridges from A-Qdca ligands into the linear chains extended in the *c* direction. Moreover, Nd2 atoms were linked by single carboxylate bridges but from B-Qdca linkers into the linear chain in the *c* direction (Figure 2d). The single neighboring chains of Nd1 and Nd2 centers were further interconnected in the *a* direction by double carboxylate bridges from A-Qdca and B-Qdca linkers forming an eight-membered wavy ring, as can be seen in packing view of the compound along the *c* axis (Figure 2e). Among these structural motifs, there were free spaces in which coordinated water molecules were located. 

The shortest (5.409(5) Å) distance between Nd atoms appeared when two carboxylate groups bridged the atoms. When neodymium atoms were joined by a single carboxylate group from A-Qdca (B-Qdca) linker, the Nd1···Nd1 and Nd2···Nd2 distances were 6.672(5) Å. The longest distances 10.037(5) Å among Nd atoms were observed when metal centers were connected by the C-Qdca ligand. As can be seen in Figure 2d, inorganic–organic layers were connected via the A-Qdca ligand. The adjacent A-Qdca ligands were located in the *c* direction in a head–tail fashion, forming stacks along the c axis. The intermolecular distances C···C in the stacks suggested the existence of π···π interactions between aromatic parts of the ligands.

**Figure 3:**
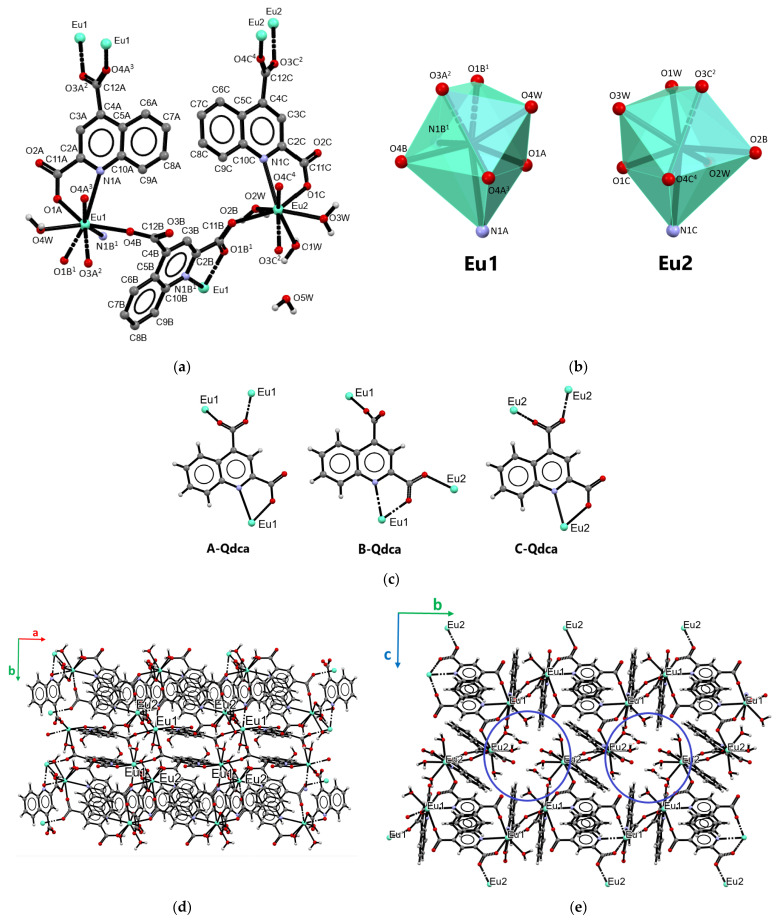
Part of the crystal structure of [Eu_2_(Qdca)_3_(H_2_O)_4_]·H_2_O (type **II**): (**a**) central metal environments in [Eu_2_(Qdca)_3_(H_2_O)_4_]·H_2_O; (**b**) coordination polyhedra of Eu atoms; (**c**) coordination modes of the Qdca^2−^ linker; (**d**) crystal packing in view along the *c* axis; and (**e**) crystal packing in view along the *a* axis.

The Nd(III), Eu(III), and Tb(III) ions formed 3D coordination polymers with quinoline-2,4-dicarboxylic acid of the general formula [Ln_2_(Qdca)_3_(H_2_O)_4_]·H_2_O (samples **2**, **3**, **6**, **7**, **8,** and **9**). Such a type (**II**) of coordination polymer was formed as the only phase for Tb(III) ions and the second phase in the case of the coordination polymers of Nd(III) and Eu(III) ions formed at 100 °C. Additionally, this type of coordination polymer was observed for Nd(III) ions at 120 °C and Eu(III) at 150 °C. The coordination polymer of europium(III) ions [Eu_2_(Qdca)_3_(H_2_O)_4_]·H_2_O (sample **8**) is described as the representative of type **II** crystal structures (Figure 3). These coordination polymers crystallized in the triclinic *P*-1 space group. The structure of complex **8** is displayed in Figure 3, and the selected bond lengths and angles are listed in Tables S4, S5, S9, S10, S14 and S15.

The asymmetric unit of the compound comprised three Qdca^2−^ anions, two crystallographically independent metal ions, and five water molecules. The coordination environment of Eu1 atom was built up from five carboxylate oxygen atoms from five Qdca^2−^ anions, two nitrogen atoms, and one oxygen atom from the aqua ligand. The second atom Eu2 was coordinated by four carboxylate oxygen atoms from four Qdca^2−^ anions, one nitrogen atom, and three oxygen atoms from water molecules. In the outer coordination sphere of the metal complex appeared one water molecule (Figure 3a). The Eu–O_carb_ bond distances were in the range 2.280(4)–2.424(4) Å, while the bond lengths of Eu–N varied from 2.689(5) to 2.830(4) Å. The Eu–O_w_ bond lengths ranged from 2.454(4) to 2.473(5) Å (Table S4). The coordination polyhedra of eight-coordinated europium(III) atoms are given in Figure 3b. In the complex [Eu_2_(Qdca)_3_(H_2_O)_4_]·H_2_O, the Qdca^2−^ anion exhibited three coordination types (Figure 3c, Tables S14, S22 and S23). The A-Qdca ligand behaved as a tetradentate bridging–chelating ligand binding three different metal centers. The carboxylate group in the 2 position acted as a monodentate group, joining the europium atom with the nitrogen from the quinoline ring into the five-membered ring. The non-planar *syn–syn* COO group in the 4 position displayed a bidentate-bridging mode (μ_2_-η^1^:η^1^). The carboxylate groups from positions 2 and 4 were rotated from the quinoline ring by 6.7°and 32.20°, respectively. The B-Qdca ligand functioned as a tetradentate bridging–chelating ligand. In comparison to the A-Qdca ligand, carboxylate group in the 4 position deviated from the heterocyclic ring by 58.63°, showing a monodenate character, while the COO group in the 2 position exhibited a *syn–anti* bidentate-bridging mode (μ_2_-η^1^:η^1^), being rotated from the quinoline ring by 10.64°. Similarly to the coordination fashion of A-Qdca, B-Qdca ligands also chelated the metal center through neighboring N and O atoms. The C-Qdca ligand showed the same coordination mode as the A-Qdca ligand but a different conformation, related to the diverse rotation of the COO group from position 4 in relation to the quinoline ring plane (39.50°) and the *syn–syn* conformation mode of COO group. The second carboxylate group of the C-Qdca linker showed a rotation by 18.58° from the plane of pyridine ring. 

In the crystal structure of the complex [Eu_2_(Qdca)_3_(H_2_O)_4_]·H_2_O, the characteristic motif in the form of layers composed of dimeric Eu1 units double-bridged by carboxylate groups from A-Qdca located in a head–tail fashion was observed (Figure 3d). Such an arrangement in this structural motif was enforced by strong π···π interactions between aromatic parts of the A-Qdca ligands with intermolecular distances C···C of 3.176(9) Å. As can be seen from the packing view along the *a* axis (Figure 3e), in the structure of complex [Eu_2_(Qdca)_3_(H_2_O)_4_]·H_2_O, a characteristic secondary building block in the form of wavy chain could also be distinguished, containing alternatively arranged metallic centers Eu2Eu1Eu1Eu2Eu2Eu1. The same type Eu1 coordination centers were bridged by two carboxylate groups (position 4) from A-Qdca linkers with a Eu1···Eu1 distance of 5.348(4) Å, while Eu2 atoms were joined by carboxylate (position 4) bridges from C-Qdca ligands with a distance of 5.426(4) Å. Eu1 and Eu2 atoms were linked by a single carboxylate bridge (position 2) from the B-Qdca ligand with a distance of 5.880(4) Å. These chains were connected by B-Qdca ligands into the two-dimensional inorganic–organic layers extended in the *bc* plane direction. Further such layers were joined by A-Qdca and C-Qdca linkers into the three-dimensional metal–organic framework. One-dimensional hexagonal channels occupied by free water molecules were also visible in the crystal packing when viewed along the *a* axis. The shape and character of such channels (Figure 3e) were defined by the coordinated water molecules making such parts of structure more polar. 

**Figure 4:**
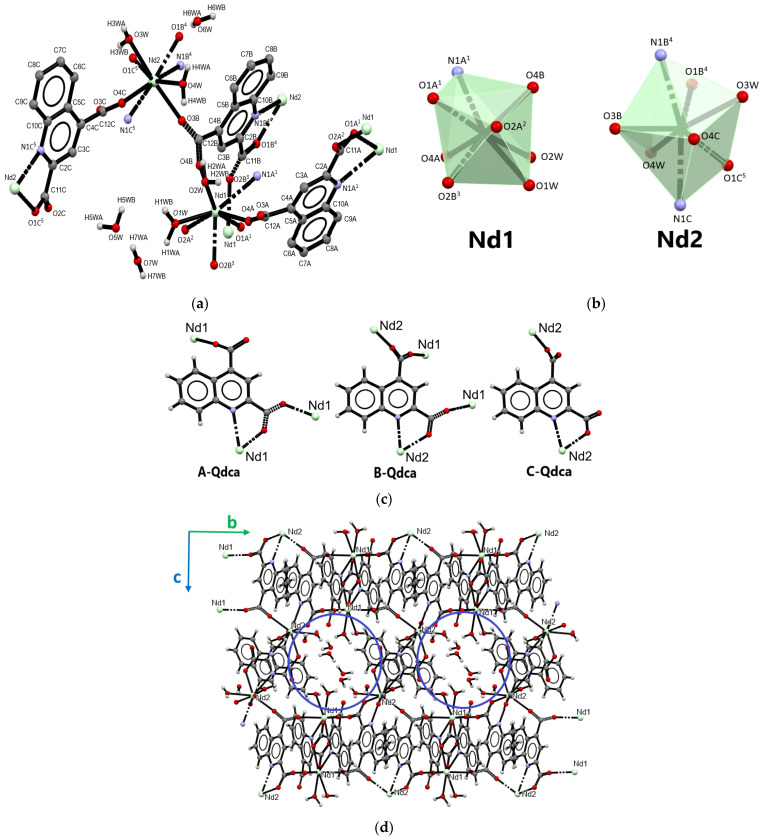
Part of the crystal structure of [Nd_2_(Qdca)_3_(H_2_O)_4_]·3H_2_O (type **III**): (**a**) central metal environments in [Nd_2_(Qdca)_3_(H_2_O)_4_]·3H_2_O; (**b**) coordination polyhedra of Nd atoms; (**c**) coordination modes of the Qdca^2−^ linker; and (**d**) crystal packing in view along the *a* axis.

The coordination polymer of neodymium(III) ions [Nd_2_(Qdca)_3_(H_2_O)_4_]·3H_2_O (sample **4**) is described as the representative of **III** type of crystal structures (samples: **4**, **5**, **10** and **11**). This metal complex crystallized in the triclinic *P*-1 space group. The structure of complex [Nd_2_(Qdca)_3_(H_2_O)_4_]·3H_2_O is displayed in Figure 4, and the selected bond lengths and angles are listed in Tables S3, S8 and S13. The asymmetric unit of the compound contained two crystallographically independent Nd(III) ions, three Qdca^2−^ anions, four coordinated water molecules, and three water molecules in the outer coordination sphere (Figure 4a). 

Both neodymium atoms adopted a coordination number of eight. The coordination sites of the Nd1 atom were occupied by five carboxylate oxygen atoms from five different Qdca^2−^ anions, one nitrogen atom, and two aqua ligands. The Nd2 atom was also coordinated by two oxygen atoms from water molecules, four carboxylate oxygen atoms, and two nitrogen atoms from four Qdca^2−^ anions (Figure 4b). The bond distances Nd–O_carb_ varied from 2.302(5) to 2.508(5) Å. The Nd–N bond lengths were in the range 2.724(5)–2.780(5) Å. The Nd–O_w_ bond lengths ranged from 2.484(6) to 2.571(7) Å (Table S3) Similarly to the above-described structures, three independent Qdca^2−^ anions appeared in the complex [Nd_2_(Qdca)_3_(H_2_O)_4_]·3H_2_O (Figure 4c, Tables S18, S22, and S23). The tetradentate bridging–chelating A-Qdca molecule coordinated three Nd1 atoms. The *syn–anti* COO group in position 2 exhibited a bidentate-bridging character (μ_2_-η^1^:η^1^), while the second COO group displayed only monodentate behavior. Additionally, this ligand chelated the Nd1 atom through neighboring N and O atoms. The carboxylate group (position 2) deviated from the plane of quinoline ring by 25.40°, while the second COO group (position 4) was rotated by 35.88°. The B-Qdca ligand showed a pentadentate bridging–chelating character and coordinated four neodymium (Nd1 and Nd2) atoms. Both COO groups exhibited a bidentate-bridging coordination mode (μ_2_-η^1^:η^1^), while their conformations were different (*syn–anti* and *anti–anti*). The COO group (in 2 position) was only rotated from the quinoline ring by 8.82°, while the second COO group was significantly deviated from the quinoline plane by 61.65°. The C-Qdca ligand exhibited a three-dentate bridging–chelating character with monodentate COO groups. These groups (positions 2 and 4) were rotated from the quinoline ring plane by 28.88° and 51.93°.

The packing view of the [Nd_2_(Qdca)_3_(H_2_O)_4_]·3H_2_O structure along *a* axis allowed distinguishing the chain of metal centers of type Nd1Nd2Nd1Nd2 in which neighboring neodymium atoms were connected by single carboxylate bridges from parallel head-to-tail B-Qdca ligands. In the *c* axis direction, Nd1 atoms were joined by two carboxylate groups (from position 2) of A-Qdca ligands with a Nd1···Nd1 distance of 5.669(5) Å. The Nd2 atoms from neighboring chains were linked by B-Qdca ligands in the *c* axis direction. In addition to the connectivity among Nd atoms, the packing view along the *a* axis clearly revealed the presence of one-dimensional channels occupied by uncoordinated water molecules (Figure 4d). The shape of pseudo-hexagonal channels was determined by the coordination environment of six metal centers Nd1Nd2Nd2Nd1Nd2Nd2. The Nd1···Nd2 distances were 6.710(5) and 6.594(5) Å, while metal sites were bridged by carboxylate groups from position 4 and position 2 of the B-Qdca ligand, respectively. The Nd2···Nd2 distances were 9.014(5) Å.

**Figure 5:**
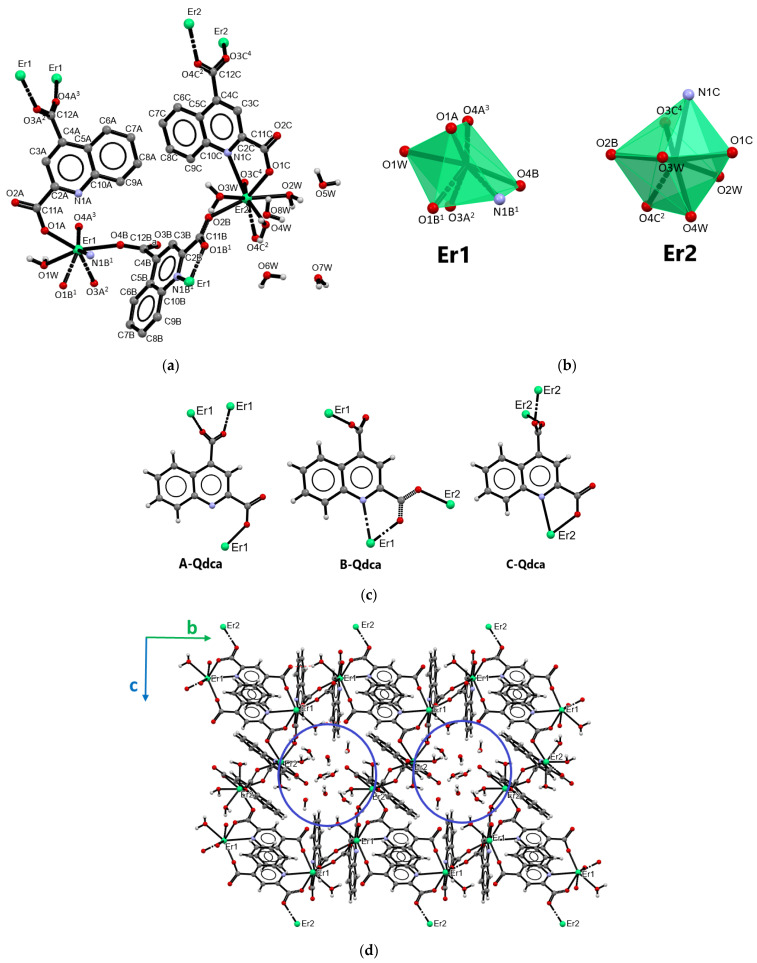
Part of the crystal structure of [Er_2_(Qdca)_3_(H_2_O)_4_]·4H_2_O (type **IV**): (**a**) central metal environments in [Er_2_(Qdca)_3_(H_2_O)_4_]·4H_2_O; (**b**) coordination polyhedra of Er atoms; (**c**) coordination modes of the Qdca^2−^ linker; (**d**) packing view along the *a* axis.

Erbium(III) ions formed the same coordination polymer (**IV** type) with quinoline-2,4-dicarboxylic acid at 100 and 120 °C of the formula [Er_2_(Qdca)_3_(H_2_O)_4_]·4H_2_O (samples **13**). Similarly to the above-described coordination polymers [Eu_2_(Qdca)_3_(H_2_O)_4_]·H_2_O and [Nd_2_(Qdca)_3_(H_2_O)_4_]·3H_2_O, the erbium compound also crystallized in the triclinic *P*-1 space group. The structure of complex [Er_2_(Qdca)_3_(H_2_O)_4_]·4H_2_O is displayed in Figure 5, and the selected bond lengths and angles are listed in Tables S6, S11 and S16. The asymmetric unit contained the same coordination components as in [Eu_2_(Qdca)_3_(H_2_O)_4_]·H_2_O, but four water molecules appeared in the outer coordination sphere. The crystal structure of the erbium(III) complex contained seven-coordinated Er1 and eight-coordinated Er2 metal centers (Figure 5a,b). The coordination environment of Er1 was composed of one aqua ligand, five carboxylate oxygen atoms from five Qdca^2−^ anions, and one nitrogen atom. The coordination sphere of Er2 atoms contained three aqua ligands, four carboxylate oxygen atoms from four Qdca^2−^ anions, and one nitrogen atom. The Er–O_carb_ bond distances were in the range of 2.236(3)–2.375(3) Å while the bond lengths of Er–O_w_ varied from 2.291(3) to 2.384(3) Å. The Er–N bond lengths were 2.601(3) and 2.715(3) Å (Table S6). The structure of **13** also exhibited three types of Qdca^2−^ anions (Figure 5c, Tables S16, S22 and S23). A-Qdca behaved as a three-dentate bridging linker coordinating Er1 atoms. Monodentate and *syn–syn* bidentate bridging carboxylate groups at positions 2 and 4 deviated from the quinoline ring by 8.20° and 44.51°, respectively. The B-Qdca ligand acted as a tetradentate bridging linker binding Er1 and Er2 atoms. Carboxylate groups at position 2 (*syn–anti*) and 4 were rotated from the quinoline ring by 11.70° and 69.12°, respectively. The C-Qdca ligand behaved as a tetradentate bridging–chelating agent coordinating three Er2 atoms. The angles among the mean plane of the quinoline ring, the monodentate COO group at the 2 position, and the bidentate-bridging (*syn–syn)* COO group at the 4 position were 8.74° and 82.27°, respectively.

As can be seen from the packing view of [Er_2_(Qdca)_3_(H_2_O)_4_]·4H_2_O, similarly to the [Eu_2_(Qdca)_3_(H_2_O)_4_]·H_2_O structure (Figure 5d), a characteristic motif could be distinguished consisting of wavy chains of erbium atoms Er1Er1Er2Er2Er1 alternatively interconnected by single (Er1Er2) and double (Er1Er1, Er2Er2) carboxylate bridges. They were further connected by Qdca^2−^ anions into the three-dimensional coordination network.

As noted above, water molecules appeared in all crystal structures both in the inner and in the outer coordination spheres, implying hydrogen-bond formation. The details of O−H···O and O−H···N hydrogen bonds in lanthanide quinoline-2,4-dicarboxylates are given in Tables S17–S21. Carboxylate oxygen atoms from C-Qdca ligands and quinoline nitrogen atoms from the B-Qdca linker appeared in the crystal structure of **1** as proton acceptor atoms in hydrogen bonds. In the crystal structures of the remaining lanthanide coordination polymers, water molecules from the outer coordination sphere appeared as additional proton donors and acceptors in O_w_−H···O_w_ hydrogen bonds.

### 2.2. PXRD Analysis and Structural Transformations

**Figure 6:**
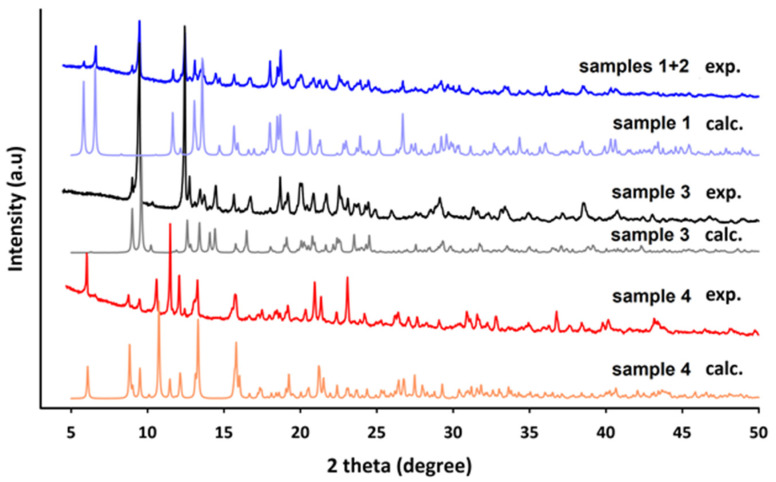
The experimental XRD patterns of neodymium(III) samples (**1–4**) synthesized at different temperatures (100, 120, and 150 °C), along with calculated XRDP from single-crystal data.

The experimental XRD patterns of polycrystalline samples prepared at 100, 120, and 150 °C (including product of recrystallization) are given in Figure 6 and Figures S1–S4. Figure 6 shows the powder X-ray diffraction patterns of all crystalline phases synthesized at various temperatures for neodymium complexes, as well as simulated XRD patterns from the determined crystal structures. Good matching of the main diffraction peak positions in the experimental and simulated XRD patterns reflected the phase purity and identity of polycrystalline samples with isolated monocrystals of coordination polymers synthesized at specific temperature. The analysis of XRD patterns of neodymium(III) quinolin-2,4-dicarboxylate prepared at 100 °C confirmed the presence of two crystal forms of **I** and **II** types. The experimental powder pattern of the europium(III) complex with the Qdca^2−^ ligand under hydrothermal conditions at 100 °C was also regarded as a mixture of crystals of **II** and **III** types.

In addition to the X-ray analysis of the as-synthesized lanthanide complexes under hydrothermal conditions, the recrystallization process of compounds formed at 120 °C was investigated. In the case of Nd(III) complex, the formation of crystals with different morphology was observed after only a few hours. Single-crystal X-ray diffraction reveled that a transformation of phase **II** into phase **III** took place. This *crystal-to-crystal* process was related to the rearrangement of the coordination sphere of neodymium(III) atoms, changes in the coordination modes and conformation of ligands, and the larger amount of uncoordinated water molecules. No structural changes were observed in the Eu(III) (phase **II**) and Er(III) (phase **IV**) complexes that were also recrystallized.

### 2.3. Infrared and Electronic Spectra

**Figure 7:**
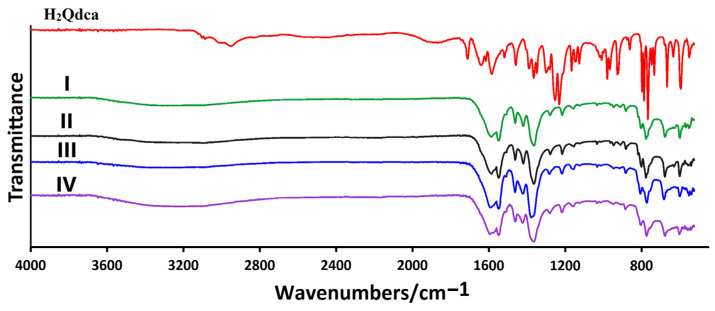
ATR/FTIR spectra of the quinoline-2,4-dicarboxylic acid and obtained lanthanide(III) complexes of the different crystal groups.

In order to characterize quinoline-2,4-dicarboxylic acid as a ligand prior to the complexation reaction, the ATR/FTIR technique was performed (Figure 7). The obtained results of the spectroscopic analysis show that the absorption bands at 3103 and 3088 cm^−1^ (range 3110–3000 cm^−1^) came from the ν(C_Ar_–H) stretching vibrations. In the range of 3100–2200 cm^−1^, a broad, strong absorption band appeared, originating from the ν(O–H) stretching vibrations. A broad band ranging from 2100 cm^−1^ to 1800 cm^−1^ was the sum of overtones from out-of-plane deformation vibrations γ(C_Ar_–H), showing the substitution of the aromatic ring. The sharp absorption band at 1711 cm^−1^ came from the stretching vibrations of the carbonyl groups ν(C=O) derived from the carboxylic groups. Vibration stretching of the aromatic ring ν(C=N, C=C) gave rise to the bands observed at wavenumbers 1639, 1616, 1585, 1518, and 1459 cm^−1^. The bands at 1390, 1365, and 1348 cm^−1^ and those at 1252 and 1232 cm^−1^ were ascribed to stretching vibrations ν(C–O) and bending vibrations δ(O–H, C–O–H). The in-plane bending vibrations of aromatic bonds δ(C_Ar_–H) gave several weak, sharp bands in the region of 1350–980 cm^−1^. The absorption bands at wavenumbers 926 and 922 cm^−1^ corresponded to the deformation vibrations γ(O–H). Sharp bands in the range from 880 to 650 cm^−1^ were ascribed to the deformation out-of-plane vibrations γ(C_Ar_–H). The bands in the range from 650 to 530 cm^−1^ belonged to out-of-plane deformation vibrations γ(C_Ar_=C_Ar_) [46,47].

The ATR/FTIR spectra of as-synthesized lanthanide(III) quinoline-2,4-dicarboxylates were very similar to each other (Figures S5–S8). All spectra were characterized by a broad band in the 3700–2700 cm^−1^ range originating from the stretching vibrations of hydroxyl groups ν(OH), proving the presence of hydrogen-bonded water molecules in the structures of the obtained complexes. The absence of characteristic bands from COOH groups confirmed the existence of deprotonation forms of the ligand in the complexes. The infrared spectra of metal complexes showed characteristic bands for the asymmetric ν_asym_(COO) and symmetric stretching vibrations ν_sym_(COO) in the regions 1552–1548 cm^−1^ and 1381–1362 cm^−1^, respectively. The great diversity of coordination modes of the Qdca^2−^ ligand in the metal complexes did not allow distinguishing different types of structures only on the basis of their infrared spectra. The disappearance in the ATR/FTIR of metal complex bands at 1639 and 1616 cm^−1^ from the aromatic skeleton, which were observed in the infrared spectrum of free H_2_Qdca acid, testified to the participation of the nitrogen atom from the quinoline ring in metal bonding.

Three different types of transitions can be found in electronic spectra of lanthanide trivalent ions: f↔d, LMCT/MLCT charge-transfer, and f↔f transitions [48,49]. Usually, f↔d and LMCT/MLCT charge-transfer transitions are not observed in electronic spectra due to the fact that the f electrons are shielded by the 5s and 5p orbitals and, thus, less affected by the ligand environment [48]. For this reason, the lanthanide ions generally exhibit absorption and emission bands in visible, near-ultraviolet, and infrared, attributed to the transitions between 4f levels, and they emerge as sharp lines with high oscillator strengths (10^−6^).

The electronic spectrum of quinoline-2,4-dicarboxylic acid (H_2_Qdca) exhibited intense absorptions with λ_max_ of about 250 nm and a shoulder band of about 220 nm, as well as a second broad asymmetric absorption with a maximum of about 340 nm and two poorly separated bands in the region of 380–400 nm which overlapped the visible range (Figures S9–S12). All of these transitions may be described as ligand-centered π–π* and n–π* transitions. The absorption spectra of lanthanide complexes showed no significant differences from the spectrum of H_2_Qdca in the range of 200–350 nm; however, the maxima of the bands exhibited a slight hypsochromic shift in comparison with free acid (Figures S9–S12). Moreover, they did not show a subtle vibronic structure, potentially indicating strong π-delocalization throughout the ligand. The visible spectra of lanthanide complexes exhibited the presence of non-separated and asymmetric absorption bands with very low intensities of about 444 nm for Tb(III) complexes and 475 nm for Eu(III) complexes only. Furthermore, low absorptions with small intensities occurred in the range of 480–670 nm and 530–880 nm in the case of Eu(III) and Nd(III) complexes, respectively, which may have been related to the 4f electron transitions.

### 2.4. Thermal Analysis

**Figure 8:**
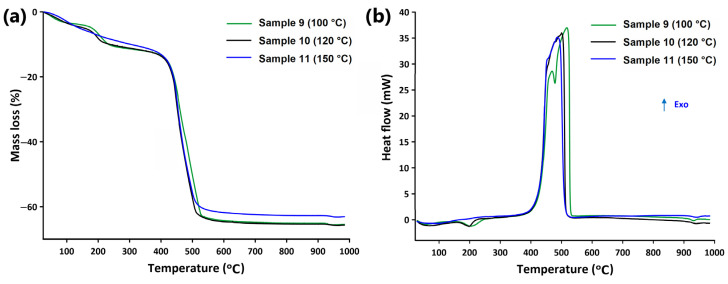
(**a**) TG and (**b**) DSC curves (air atmosphere) of terbium complexes (samples **9–11**) prepared at 100, 120, and 150 °C.

The thermal behavior of as-synthesized lanthanide(III) quinoline-2,4-dicarboxylates was investigated using simultaneous TG-DSC methods in air atmosphere (Figure 8 and Figures S13–S15, Table S24). It was difficult to find significant differences in their thermal decomposition due to their high structural similarity. Additionally, neodymium(III) and europium(III) samples prepared at 100 °C were obtained as the mixture of two crystal phases. It was impossible to separate them; therefore, the recorded TG-DSC curves refer to the mixtures of compounds. In general, all investigated lanthanide(III) complexes were stable in air atmosphere at room temperature and then started to exhibit multi-step decomposition during heating. The first step in the temperature range of 30–357 °C corresponded to the release of both coordinated and uncoordinated water molecules in the overlapping stages. Dehydration processes were accompanied by the endothermic effects observed on the DSC curves. It is worth emphasizing that the dehydrated forms of lanthanide(III) complexes were unstable. This fact suggests that the elimination of coordinated water molecules from the coordination sphere of atom centers implied the formation of unsaturated metal nodes that were most probably crucial for the decomposition process [50]. Further heating caused their decomposition and burning, accompanied by significant mass losses (about 50%) recorded on the TG curves associated with strong exothermic effects. As the solid residues of metal complex heating in air atmosphere, the corresponding metal oxides Nd_2_O_3_, Eu_2_O_3_, Tb_4_O_7_, and Er_2_O_3_ were formed above 600 °C [51,52,53].

The profiles of TG-DSC curves for neodymium samples obtained at 100 and 120 °C were very similar in spite of the fact that two crystal phases appeared at 100 °C. Their dehydration process occurred in two quite well-separated stages in the temperature ranges 30–135 and 30–122 °C for samples prepared at 100 and 120 °C, respectively. Next, further elimination of water molecules took place up to 357 and 345 °C with mass losses of 7.49% and 8.33%, respectively. Endothermic effects recorded at 188 and 193 °C for phases as-synthesized at 100 and 120 °C corresponded to the removal of coordinated water molecules. In the case of the amorphous phase obtained at 150 °C, only a gradual mass loss of 10.54% in the temperature range 30–304 °C without a visible effect on the DSC curve was recorded. At higher temperature, the decomposition of neodymium samples took place, accompanied by several exothermic effects on DSC curves. In all cases, the formation of some unstable intermediate products at about 520 °C was postulated from the profiles of TG and DSC curves. The final solid product, i.e., neodymium(III) oxide, was formed above 675 °C.

The dehydration process of the europium(III) sample prepared at 100 °C proceeded in four overlapping steps, as can be deduced from the profiles of TG and DSC curves. In the range of 30–326 °C, mass losses of 3.05%, 1.67%, 2.17%, and 6.03% were observed. These steps were related to the liberation of inner and outer coordination water molecules from both types of crystal phases (**II** and **III**). The endothermic effects associated with these stages were recorded at 152 and 199 °C. Next, the decomposition of the dehydrated forms of complexes took place along with burning processes. The final product Eu_2_O_3_ was formed at 697 °C. The TG curves of europium(III) samples obtained at 120 and 150 °C, which presented the same type of crystal structure, exhibited a two-step dehydration process. The first mass loss occurred up to about 150 °C, while the second one occurred up to 295 °C. The removal of coordinated water molecules, which most probably occurred at higher temperature, was reflected by the presence of endothermic effects at 192 and 205 °C on the DSC curves. Next, the formation of europium(III) oxide was observed as the result of decomposition and burning of desolvated forms of complexes.

The dehydration of terbium(III) complexes as-synthesized at 100 and 120 °C occurred in two overlapping stages in the corresponding temperature ranges of 30–122, 30–132 °C and 123–325, 133–314 °C, respectively (Figure 8). Similarly to the previously described compounds, the second stage of water molecule liberation was associated with a strong endothermic effect, whereas the first step was accompanied by a weak effect. The terbium(III) complex obtained at 150 °C showed a gradual mass loss ranging from 30 to 323 °C at the dehydration temperature, without a distinctive energetic effect on the DSC curve. Above 690 °C, all terbium complexes decomposed into the Tb_4_O_7_ oxide.

The dehydration process of the erbium(III) complex obtained at 100 °C occurred in three distinct stages above 30 °C. The mass loss of 4.80% recorded in the range of 30–114 °C was accompanied by an endothermic effect at 69 °C (peak top) on the DSC curve. This stage could be attributed to the removal of weakly bonded, most probably uncoordinated water molecules located in the 1D channel presence in its structure. At higher temperature, two well-separated mass losses of 3.55% and 5.10%, derived from the elimination of coordinated water molecules in the temperature ranges 115–190 and 190–330 °C, appeared on the TG curve. Both stages were accompanied by endothermic effects at 170 and 226 °C. For erbium(III) complexes synthesized at 120 and 150 °C, only gradual mass losses of 18.10% and 20.30% were observed on TG curves, not accompanied by endothermic effects. Above 330 °C, the decomposition of desolated forms of complexes along with the burning process yielded erbium(III) oxide formation as the final solid product of heating.

### 2.5. Luminescence Investigations

**Figure 9:**
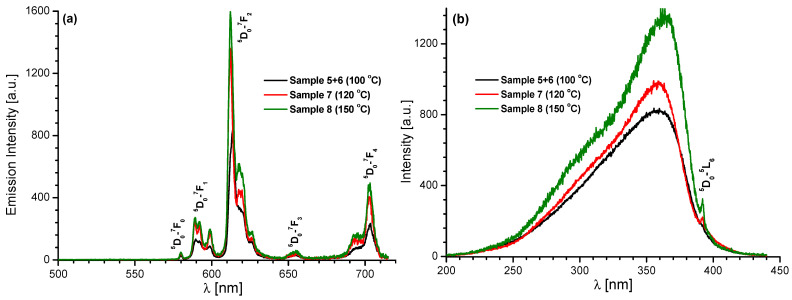
Solid-state emission spectra (**a**, λ_ex_ = 360 nm) and excitation spectra (**b**, obtained monitoring emission on the hypersensitive ^5^D_0–_^7^F_2_ transition of the Eu(III) ion at 612 nm) of Eu(III) samples prepared at different temperatures (100, 120, and 150 °C).

**Figure 10:**
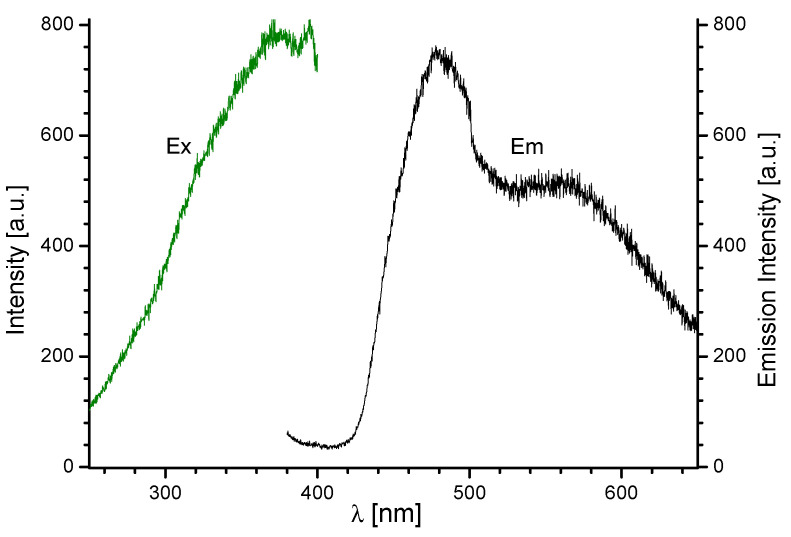
The excitation and emission spectra of H_2_Qdca ligand recorded at room temperature (λ_ex_ = 360 nm, λ_ob_ = 480 nm).

In order to characterize the optical properties of the investigated materials, their emission spectra were recorded. Figure 9 and Figure S16 demonstrates the emission spectra of the Eu(III) complexes prepared at 100, 120, and 150 °C recorded under 360 and 392 nm excitation at room temperature. As presented in Figure 9a, Eu(III) exhibited an intense luminescent emission band at 612 nm upon excitation at 360 nm, confirming the efficient energy transfer from the ligand to the excited states of the europium ions. To confirm and check the excitation wavelengths for 612 nm emission, the excitation spectra were monitored, as shown in Figure 9b. For comparison, Figure 10 shows the excitation and emission spectra of the H_2_Qdca ligand. As can be seen from Figure 10, the H_2_Qdca ligand showed a broad band in the blue-green region, assigned to π–π transitions. The very intense metal-centered emission observed in the Eu(III) compounds and the lack of a broad emission band coming from the organic ligand molecules observed in the blue-green region indicated an effective energy transfer from the excited level of the ligand to the europium(III) ions. In addition, the intensity of this emission was four times lower when the complex was excited using λ_ex_ = 392 nm, typical for the Eu(III) ion (Figure S16). This also confirmed the occurrence of intramolecular energy transfer from the H_2_Qdca ligand to Eu(III) ions.

Furthermore the broad and intensive band with the intensity maximum at 360 nm (Figure 9b) and weak band correspondence to ^7^F_0_ → ^5^L_6_ transition in Eu(III) ions (392 nm) gave evidence that the europium luminescence sensitizations through intramolecular energy transfer from the Qdca^2−^ anion to Eu(III) ion were efficient. 

From Figure 9a, it can be seen that the samples, excited at 360 nm, showed five narrow spectral lines in the range of 500−720 nm, characteristic for Eu(III) ions. The emissions at 580, 590, 612, 655, and 703 nm corresponded to the transitions ^5^D_0_ → ^7^F_0_, ^5^D_0_ → ^7^F_1_, ^5^D_0_ → ^7^F_2_, ^5^D_0_ → ^7^F_3_, and ^5^D_0_ → ^7^F_4_, respectively, with the dominant band at 612 nm being the so-called “hypersensitive” transition. This is an electric-dipole emission which is forbidden in free ions. The intensity of the ^5^D_0_ → ^7^F_2_ transition was greater than that of the ^5^D_0_ → ^7^F_1_ transition (magnetic dipole). The intensity ratio of the ^5^D_0_ → ^7^F_2_ transition to ^5^D_0_ → ^7^F_1_ transition of Eu(III) compounds was 6.2, 5.2, and 5.9, respectively. These results could most likely be ascribed to the non-centrosymmetric coordination environment around the Eu(III) centers [33,38,54].

The intensity of emission of the europium(III) coordination polymers prepared at 120 and 150 °C was similar and higher than in the sample prepared at 100 °C (Figure 9a). This observation is in good agreement with the crystal structures of europium complexes. The same coordination polymer of the formula [Eu_2_(Qdca)_3_(H_2_O)_4_]·H_2_O was formed at 100 °C, whereas, at 120 and 150 °C, a mixture of [Eu_2_(Qdca)_3_(H_2_O)_4_]·H_2_O and [Eu_2_(Qdca)_3_(H_2_O)_4_]·3H_2_O phases was observed. It can be postulated that the presence of a crystal phase with a greater number of outer coordination water molecules yielded a decrease in luminescence intensity. In other words, a reduction in the number of non-coordinated water molecules in the structure of europium complexes resulted in a substantial enhancement of luminescence intensity. This is in accordance with the observations made by Salaam et al. [55]. Under identical experimental conditions, no emission was observed in the case of Tb(III) complexes.

To elucidate the energy transfer processes in the Eu(III) complex, the triplet state energy of the Qdca^2−^ anion was determined. The phosphorescence spectrum of [Gd_2_(Qdca)_3_(H_2_O)_4_]·H_2_O was measured at −196 °C in ethanol. The gadolinium(III) complex was selected for the determination of the triplet state energy of the Qdca^2−^ anion because the triplet energy level of the ligand is not significantly affected by the Ln(III) ions, and the lowest-lying excited level of the Gd(III) ion, ^6^P_7/2_, is located above 32,000 cm^−1^ [33]. Thus, the phosphorescence spectrum of [Gd_2_(Qdca)_3_(H_2_O)_4_]·H_2_O allowed evaluating the triplet energy level Qdca^2−^ for all our studied lanthanide(III) complexes. From this recorded spectrum, the triplet state energy of Qdca^2−^ was determined to be 21,322 cm^−1^ on the basis of the maximum of the phosphorescence band located at 469 nm (as shown in Figure S17). The triplet level of Qdca^2−^ was found to be moderately higher than that of 2,4′-biphenyldicarboxylic acid and slightly lower than that of 4-quinoline carboxylic acid [56,57]. In order to transfer the energy from the ligand to the lanthanide ion, the triplet state energy needs to be higher than the resonance level of the metal ion. The excited levels of Eu(III) ^5^D_0_, and Tb(III) ^5^D_4_ are 17,265 and 20,500 cm^−1^, respectively [33]. The energy differences between the triplet state of Qdca^2−^ and the resonance energy level of ^5^D_0_ and ^5^D_4_ (E(T1) − E(^5^D_j_)]) can be calculated as 4057 and 822 cm^−1^, respectively. A difference of 2000–5000 cm^−1^ is necessary to efficiently sensitize the luminescence of the lanthanide [58,59]. These energy differences show that Qdca^2−^ can effectively sensitize Eu(III) ions. On the other hand, the energy back transfer is responsible for the lack of emission of Tb(III) ions [60]. Latva et al. reported that the energy back transfer occurs when the difference between the lowest energy level of the ligand in the triplet state and the ^5^D_4_ emission level of the Tb(III) ion is less than 1850 cm^−l^ [59].

In order to complete spectroscopic analysis of the obtained europium(III) coordination polymers, the quantum yield and luminescent lifetimes were measured and analyzed. The luminescence decay curves of these complexes were investigated at the maximal excitation and emission wavelengths (Figure S18). The decay curves for Eu(III) samples prepared in 100 °C and 150 °C were adjusted with a single exponential function, and the lifetime values (τ) of the ^5^D_0_ emitting level were found to be 202.7 ± 0.2 and 200.5 ± 0.2 μs, respectively. The experimental data were fitted with *R*^2^ = 0.9980 using a single exponential function. Because the luminescence lifetime of Ln(III) is very sensitive to the composition of the inner coordination sphere [61,62], the short lifetimes determined herein can be attributed to the presence of luminescence quenchers, H_2_O molecules, in the coordination spheres of Eu(III) ions. The numbers of water molecules in these compounds calculated from Kimura’s empirical rule [63] were determined to be 4.5 (with an accuracy of 0.5 molecule). The obtained results indicate the presence of water molecules in the first coordination sphere of the metal ions as shown earlier. The emission quantum efficiency of the ^5^D_0_ emitting level of the Eu(III) ion for the sample prepared at 150 °C was determined according to Equation (1)

(1) $$\phi =\frac{I_{emEu^{3+}}}{I_{exLa_{2}O_{3}}-I_{exEu^{3+}}}\cdot 100\%$$

where *ϕ* is the quantum yield, $I_{emEu^{3+}}$ is the integrated intensity of sample luminescence, and $I_{exLa_{2}O_{3}}$ and $I_{exEu^{3+}}$ are the integrated intensities of scattered excitation radiation not absorbed by the reference (La_2_O_3_) and the integrated intensities of the scattered excitation light for the investigated material, respectively. All procedures were carried out in accordance with [64,65]. The luminescence quantum yield was calculated as 0.023.

**Figure 11:**
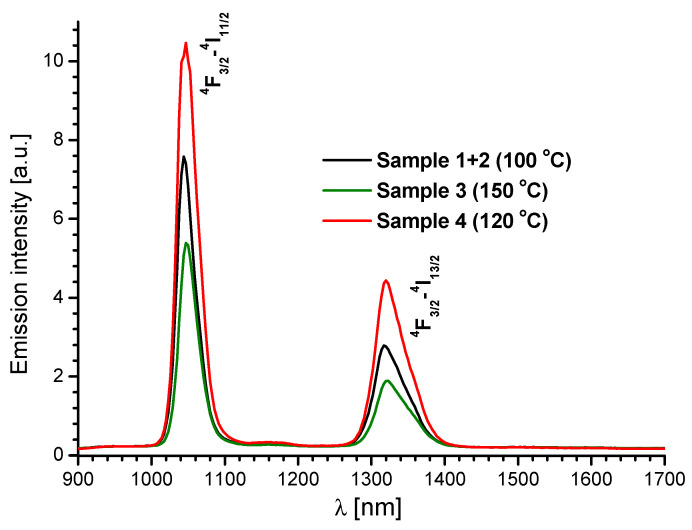
Solid-state NIR emission spectra of Nd(III) complexes prepared at 100, 120, and 150 °C, recorded at room temperature (λ_ex_ = 369 nm).

Figure 11 presents the emission spectra of the Nd(III) coordination polymers. These spectra were recorded at room temperature under 369 nm LED pumping, in the near-infrared (NIR) region from 900 to 1700 nm. The complexes show characteristic NIR emissions of the Nd(III) and Er(III) ions. The emission spectra of Nd(III) arose from the excited state ^4^F_3/2_ in the form of two transitions, ^4^F_3/2_→^4^I_11/2_ (1047 nm) and ^4^F_3/2_→^4^I_13/2_ (1323 nm) [66]. All the emission peaks were identical, except for the peak intensities. The ^4^F_3/2_→^4^I_11/2_ transition (1047 nm) was the most intense and dominated the emission spectra of the complexes. Slight differences in the intensity of these transitions could be attributed to the slightly different number of water molecules in these compounds, as shown in Table 1. The energy of the triplet state of Qdca^2−^ existed 9795 cm^−1^ above the ^4^F_3/2_ (11,527 cm^−1^) emitting level of Nd(III). The energy gap between the ligand triplet state and the emitting level of lanthanide ion in the case of the present Nd(III) complexes is larger than the optimum range for effective transfer of energy. Therefore, non-radiative decay of excited states can occur with high efficiency, resulting in lower emission intensity of Nd(III) ions.

**Figure 12:**
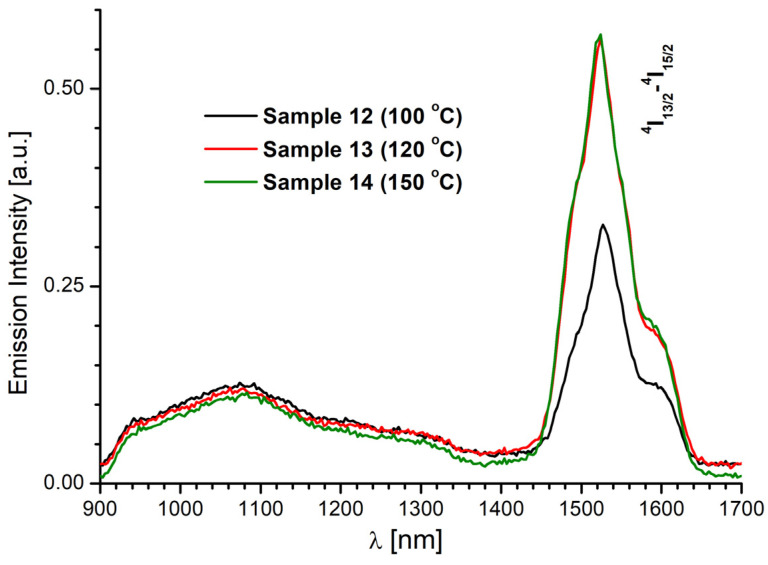
Solid-state NIR emission spectra of Er(III) complexes prepared at 100, 120, and 150 °C, recorded at room temperature (λ_ex_ = 369 nm).

The NIR emission plot of Er(III) complexes, measured in the spectral range of 900–1700 nm, upon the excitation at 369 nm is presented in Figure 12. The spectra show broad and weak bands with maximum intensity at around 1524 nm, assigned to the ^4^I_13/2_→^4^I_15/2_ transition of Er(III) ions. As in the case of europium and neodymium compounds, an increase in the temperature at which the synthesis was carried out favored better emission properties of the samples [67].

## 3. Materials and Methods

Hydrates of lanthanide(III) nitrates(V) (99.9%) were purchased from Merck and used without further purification. Quinoline-2,4-dicarboxylic acid (95%) was purchased from Alfa Aesar and purified by heating with distilled water.

### 3.1. Synthetic Procedures

**Synthesis of [Ln_2_(Qdca)_3_(H_2_O)_x_]·yH_2_O.** The hydrothermal synthesis of lanthanide(III) complexes required two steps. In the first step, dissolution and deprotonation of H_2_Qdca acid were performed using a 0.2 M NaOH solution. In the second stage, the synthesis of complexes was carried out by mixing aqueous solutions of Ln(NO_3_)_3_ (1.9 mmol, 20 mL) (where Ln(III) = Nd, Eu, Tb, and Er) with sodium salt of acid (2.3 mmol, 25 mL). The pH of the reaction mixture was 5. The obtained mixtures were placed in 100 mL Teflon vessels and enclosed in steel autoclaves. In order to achieve hydrothermal conditions, the synthesis proceeded at the temperatures of 100 °C, 120 °C, and 150 °C for 72 h. Then, the autoclaves were allowed to cool to room temperature. The obtained suspensions were filtered, and the resulting precipitates were washed with distilled water and dried in air.

The recrystallization process of the amorphous phases of Nd(III) and Er(III) complexes hydrothermally obtained at 150 °C, as well as of metal complexes prepared at 120 °C, was performed by adding 10 mL of distilled water to about 100 mg of pristine metal complexes, followed by stirring (30 min.). After 24 h, in the case of all samples, new crystal phases were noticed.

Additionally, the gadolinium(III) complex ([Gd_2_(Qdca)_3_(H_2_O)_4_]·H_2_O) was prepared according to the procedure given above (hydrothermal synthesis at 120 °C) for luminescence analysis purposes.

### 3.2. Analytical Methods

The contents of C, H, and N in the obtained compounds were determined by elemental analysis using a EuroEA Elemental Analyzer, and the results are given in Table S1 (Supplementary Materials).

The X-ray powder diffraction patterns of the prepared complexes were obtained by means of an Empyrean powder diffractometer PANalytical using the Bragg–Brentano method.

Optical images of the crystals were taken on a scanning confocal microscope MA200 Nikon. Images of the europium complexes were obtained using an excitation wavelength of 420 nm.

The single-crystal X-ray measurements on obtained compounds were obtained using the Rigaku XtaLAB MM7HFMR diffractometer equipped with a “quarter-chi single” goniometer, rotating anode generator (graphite monochromated Cu Kα radiation), and Pilatus 200 K detector. The CrysAlisPro 1.171.39.27b program was used for data collection, cell refinement, and data reduction [68]. The structures were solved using the direct methods implemented in SHELXS-97 and refined with the SHELXL-18/3 program, both operating under WinGX [69,70]. All hydrogen atoms attached to water oxygens were found in the difference Fourier maps and initially freely refined. In the final refinement cycles, the O_w_-bonded H atoms were repositioned in their calculated positions and refined with the O–H bond lengths restrained to 0.840(2) Å, H∙∙∙H distances restrained to 1.380(2) Å, and U_iso_(H) = 1.5 U_eq_(O). The hydrogen atoms attached to carbon were positioned geometrically and refined using a riding model with U_iso_(H) = 1.2 U_eq_(C).

The weak quality of the crystals of obtained erbium(III) quinoline-2,4-dicarboxylate compound synthesized at 150 °C allowed only determining the unit cell parameters.

The infrared spectra (ATR/FTIR) of the ligand and the obtained compounds were recorded using a Nicolet 6700 spectrophotometer equipped with the Smart iTR accessory (diamond crystal) in the range of 4000–600 cm^−1^ (Thermo Scientific, Waltham, MA, USA)

Solid electronic spectra for H_2_Qdca and complexes were recorded on a PerkinElmer Labmda 365+ double bean UV/Vis spectrometer, with BaSO_4_ as the reference.

Thermal analyses of the prepared complexes were made by applying the thermogravimetric (TG) and differential scanning calorimetry (DSC) methods using the SETSYS 16/18 analyzer (Setaram, Caluire, France). The samples (about 5–9 mg weighed on the WAX62 (Radwag wagi elektroniczne, Radom, Poland) analytical balance with a precision of 0.01 mg) were heated in alumina crucibles up to 1000 °C at a heating rate of 10 °C min^−1^ in a dynamic air atmosphere (v = 0.75 dm^3^ h^−1^).

The emission and excitation spectra in the UV/Vis range were recorded on a Hitachi F7000 Fluorescence Spectrometer using a 450 W Xenon lamp, with a wavelength range of 200–800 nm. The luminescence spectrum of the gadolinium complex in ethanol solution was recorded at 148 °C using the same equipment, but operating in phosphorescence mode. The photoluminescence spectroscopy experiments in the NIR range were performed in an Andor Shamrock 500 spectrometer (300 l/mm—blaze 1200 nm) equipped with a CCD camera iDus 420 and spectrometer QuantaMasterTM 40 s (Photon Technology Instrumental, Pemberton, NJ, USA) equipped with Photomultiplier Tubes H10330C-75 (900–1800 nm). The samples were excited using an LED revolver with high-power 360–370 nm LEDs. Excitation and emission spectra were corrected for the instrumental response. Emission intensity measurements were carried out using the adapter and holder supplied by the manufacturer of the spectrofluorometer, for emission measurements of solid samples. The QuantaMasterTM 40 (Photon Technology International) spectrophotometer equipped with an Opolette 355LD UVDM (Opotek Incorporation, Carlsbad, CA, USA) tunable laser (excitation source), which had a repetition rate of 20 Hz as the excitation source, and a Hamamatsu R928 photomultiplier as a detector, was used to measure luminescence decays. All measurements were collected at room temperature. 

## 4. Conclusions

**Figure 13:**
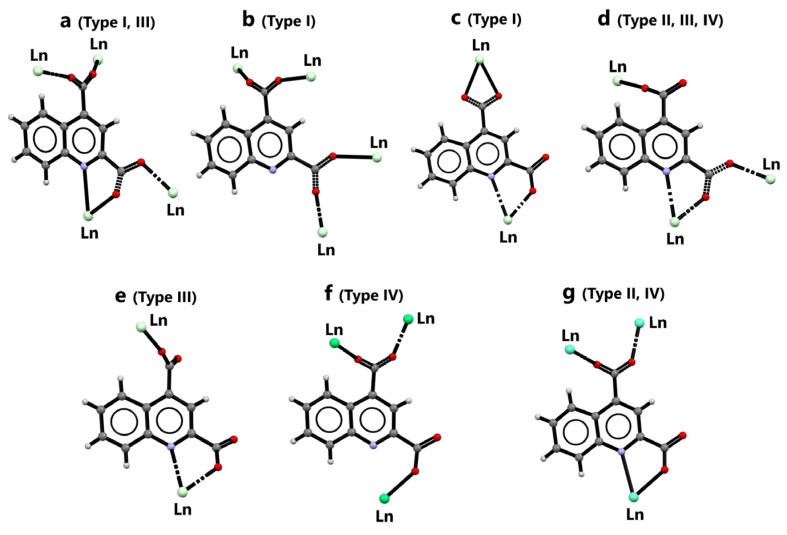
Coordination modes (**a**–**g**) of quinoline-2,4-dicarboxylate ligand observed in **I**–**IV** crystal structure types.

We demonstrated the first examples of four structurally characterized groups of novel three-dimensional coordination polymers built up from selected lanthanides (Nd(III), Eu(III), Tb(III), and Er(III)) and quinoline-2,4-dicarboxylate ligand as the building block. We showed that the structural diversity of coordination polymers was prompted by temperature. In general, a higher temperature of hydrothermal synthesis was advantageous for the formation of more hydrated metal complexes. Only one crystal phase of Nd (at 100 °C) was formed without non-coordinated water molecules, whereas the remaining 3D coordination polymers exhibited structures with one-dimensional channels occupied by one, three, or four water molecules. It can be assumed that the coordination and conformation flexibility of Qdca^2−^ anions, which exhibited seven different coordination modes in the prepared complexes (Figure 13), was also affected by temperature. 

The europium complexes exhibited luminescence in the red spectral range, while the erbium and neodymium complexes displayed weak emission in the NIR region. The Qdca^2−^ anion effectively sensitized Eu(III) ions, while, for Tb(III) ions, back transfer energy was observed. The number of water molecules in the structure of complexes was crucial for luminescence intensity. 

## Figures and Tables

**Table 1:** The main crystallographic parameters along with composition, numbering of investigated samples (**1**–**14**), and crystal structure types (**I**–**V**).

Complex/ Temperature	100 °C		120 °C	150 °C
	Crystal Structure Type x; y (Number of Water Molecules from Inner and Outer Coordination Sphere) Crystal System, Space Group a/b/c [Å] α/β/ƴ [°]			
**[Nd_2_(Qdca)_3_(H_2_O)x]·yH_2_O**	**Type-I** x = 3; y = 0 (**1**) orthorhombic, *Pna*2_1_ 14.985/30.366/6.672 90/90/90	**Type-II** x = 4; y = 1 (**2**) triclinic, *P*-1 9.932/12.315/14.211 89.697/82.258/86.568	**Type-II** x = 4; y = 1 (**3**) triclinic, *P*-1 9.946/12.323/14.184 89.901/81.851/86.24	**Type-III** x = 4; y = 3 (**4**) triclinic, *P*-1 10.593/11.806/15.040 97.070/101.073/103.862
**[Eu_2_(Qdca)_3_(H_2_O)x]·yH_2_O**	**Type-III** x = 4; y = 3 (**5**) triclinic, *P*-1 10.531/11.698/14.9681 96.498/101.195/104.43	**Type-II** x = 4; y = 1 (**6**) triclinic, *P*-1 9.859/12.339/14.144 89.89/97.53/93.55	**Type-II** x = 4; y = 1 (**7**) triclinic, *P*-1 9.853/12.305/14.075 90.02/98.182/93.877	**Type-II** x = 4; y = 1 (**8**) triclinic, *P*-1 9.858/12.3124/14.055 90.133/98.245/94.016
**[Tb_2_(Qdca)_3_(H_2_O)x]·yH_2_O**	**Type-II** x = 4; y = 1 (**9**) triclinic, *P*-1 9.802/12.309/13.988 90.212/98.326/94.157		**Type-III** x = 4; y = 3 (**10**) triclinic, *P*-1 10.492/11.608/14.911 96.144/101.267/104.977	**Type-III** x = 4; y = 3 (**11**) triclinic, *P*-1 10.492/11.632/14.969 96.000/101.459/104.980
**[Er_2_(Qdca)_3_(H_2_O)x]·yH_2_O**	**Type-IV** x = 4; y = 4 (**12**) triclinic, *P*-1 9.937/12.285/15.121 84.246/86.472/89.165		**Type-IV** x = 4; y = 4 (**13**) triclinic, *P*-1 9.936/12.269/15.093 84.151/86.415/89.199	**Type-V** x + y = 14 (**14**) triclinic, *P*-1 11.75/13.39/15.37 91.27/105.56/114.09

**Table 2:** Crystal data and structure refinement details for crystals **1**, **4**, **8**, **9**, and **13**.

Compound (Sample Number)	[Nd_2_(Qdca)_3_(H_2_O)_3_] (1)	[Nd_2_(Qdca)_3_(H_2_O)_4_]·3H_2_O (4)	[Eu_2_(Qdca)_3_(H_2_O)_4_]·H_2_O (8)	[Tb_2_(Qdca)_3_(H_2_O)_4_]·H_2_O (9)	[Er_2_(Qdca)_3_(H_2_O)_4_]·4H_2_O (13)
Empirical formula	C_33_H_21_N_3_O_15_Nd_2_	C_33_H_29_N_3_O_19_Nd_2_	C_33_H_25_N_3_O_17_Eu_2_	C_33_H_25_N_3_O_17_Tb_2_	C_33_H_31_N_3_O_20_Er_2_
Formula weight	988.01	1060.07	1039.48	1053.40	1124.13
T/K	295(2)	295(2)	295(2)	295(2)	295(2)
Crystal system	*orthorhombic*	*triclinic*	*triclinic*	*triclinic*	*triclinic*
Space group	*Pna*2_1_	*P*-1	*P*-1	*P*-1	*P*-1
*a/*Å	14.9850(1)	10.5934(4)	9.8575(4)	9.8018(4)	9.9361(2)
*b/*Å	30.3661(2)	11.8063(4)	12.3124(4)	12.3088(5)	12.2695(2)
*c/*Å	6.6721(1)	15.0403(3)	14.0546(4)	13.9881(5)	15.0930(3)
*α/*°	90	97.070(2)	90.133(2)	90.212(3)	84.151(1)
*β/*°	90	101.073(3)	98.245(3)	98.326(3)	86.415(1)
*γ/*°	90	103.862(3)	94.016(3)	94.157(3)	89.199(2)
*Volume/*Å^3^	3036.05(5)	1763.8(1)	1683.9(1)	1665.29(1)	1826.79(6)
*Z*	4	2	2	2	2
*d*_calc_/g∙cm^3^	2.162	1.996	2.050	2.101	2.044
*μ*/mm^−1^	26.57	23.00	27.16	21.39	9.09
2Θ range/°	8.29–136.85	7.84–136.94	7.20–138.06	7.20–136.83	8.87–136.03
Ref. collected	18,174	26,149	23,719	20,895	20,077
Independent reflections	5154 [R_int_ = 0.0326]	6384 [R_int_ = 0.0539]	6084 [R_int_ = 0.0437]	6004 [R_int_ = 0.0446]	6525 [R_int_ = 0.0228]
Data/restr./ parameters	5154/10/494	6384/21/518	6084/15/526	6004/13/526	6525/23/568
GooF on F^2^	1.020	1.110	1.044	1.100	1.064
Final R_1_, *w*R_2_ indices [*I* > 2*σ*(I)]	0.0301, 0.0789	0.0504, 0.1381	0.0395, 0.1079	0.0477, 0.1316	0.0315, 0.0874
Final R_1_, *w*R_2_ indices [all data]	0.0308, 0.0794	0.0574, 0.1428	0.0458, 0.1119	0.0541, 0.1364	0.0322, 0.0879
Largest diff. peak/hole/e Å^−3^	1.40/−0.91	0.84/−1.70	1.35/−1.43	0.91/−2.26	1.39/−1.45
Flack parameter	−0.016(4)	-	-	-	-
CCDC number	2281669	2281670	2281671	2281672	2281673

## Data Availability

The data underlying this article will be shared on reasonable request from the corresponding authors.

## Supplementary Materials

The following supporting information can be downloaded at https://www.mdpi.com/article/10.3390/molecules28176360/s1: Figure S1. X-ray diffraction patterns of neodymium(III) samples obtained at different temperatures (100, 120, and 150 °C); Figure S2. X-ray diffraction patterns of europium(III) samples obtained at different temperatures (100, 120, and 150 °C); Figure S3. X-ray diffraction patterns of terbium(III) samples obtained at different temperatures (100, 120, and 150 °C); Figure S4. X-ray diffraction patterns of erbium(III) samples obtained at different temperatures (100, 120, and 150 °C); Figure S5. ATR/FTIR spectrum of neodymium(III) samples obtained at different temperatures (100, 120, and 150 °C); Figure S6. ATR/FTIR spectrum of europium(III) samples obtained at different temperatures (100, 120, and 150 °C); Figure S7. ATR/FTIR spectrum of terbium(III) samples obtained at different temperatures (100, 120, and 150 °C); Figure S8. ATR/FTIR spectrum of erbium(III) samples obtained at different temperatures (100, 120, and 150 °C); Figure S9. UV/Vis spectra of the neodymium(III) samples obtained at different temperatures (100, 120, and 150 °C); Figure S10. UV/Vis spectra of the europium(III) samples obtained at different temperatures (100, 120, and 150 °C); Figure S11. UV/Vis spectra of the terbium(III) samples obtained at different temperatures (100, 120, and 150 °C); Figure S12. UV/Vis spectra of the erbium(III) samples obtained at different temperatures (100 and 120 °C); Figure S13. (**a**) TG and (**b**) DSC curves of neodymium(III) complexes in air atmosphere, obtained at different temperatures (100, 120, and 150 °C); Figure S14. (**a**) TG and (**b**) DSC curves of europium(III) complexes in air atmosphere, obtained at different temperatures (100, 120, and 150 °C); Figure S15. (**a**) TG and (**b**) DSC curves of erbium(III) complexes in air atmosphere, obtained at different temperatures (100, 120, and 150 °C); Figure S16. Room-temperature emission spectra of [Eu_2_Qdca_3_ (H_2_O)_4_]·H_2_O in solid state (λ_ex_ = 360 and 392 nm); Figure S17. Low-temperature phosphorescence spectrum of [Gd_2_(Qdca)_3_(H_2_O)_4_]·H_2_O in ethanol solution at 148 °C (λ_ex_ = 360 nm); Figure S18. Typical luminescence decay profiles observed for Eu(III) complexes in the solid state at room temperature prepared at 100 °C and 150 °C (blue points are the raw data, while the red line is a monoexponential fit); Table S1. Results of the elemental analysis of prepared complexes; Table S2. Bond lengths for (1) [Nd_2_(Qdca)_3_(H_2_O)_3_]; Table S3. Bond lengths for (4) [Nd_2_(Qdca)_3_(H_2_O)_4_]·3H_2_O; Table S4. Bond lengths for (8) [Eu_2_(Qdca)_3_(H_2_O)_4_]·H_2_O; Table S5. Bond lengths for (9) [Tb_2_(Qdca)_3_(H_2_O)_4_]·H_2_O; Table S6. Bond lengths for (13) [Er_2_(Qdca)_3_(H_2_O)_4_]·4H_2_O; Table S7. Bond angles for (1) [Nd_2_Qdca_3_(H_2_O)_3_]; Table S8. Bond angles for (4) [Nd_2_(Qdca)_3_(H_2_O)_4_]·3H_2_O; Table S9. Bond angles for (8) [Eu_2_(Qdca)_3_(H_2_O)_4_]·H_2_O; Table S10. Bond angles for (9) [Tb_2_(Qdca)_3_(H_2_O)_4_]·H_2_O; Table S11. Bond angles for (13) [Er_2_(Qdca)_3_(H_2_O)_4_]·4H_2_O; Table S12. Torsion angles for (1) [Nd_2_Qdca_3_(H_2_O)_3_]; Table S13. Torsion angles for (4) [Nd_2_(Qdca)_3_(H_2_O)_4_]·3H_2_O; Table S14. Torsion angles for (8) [Eu_2_(Qdca)_3_(H_2_O)_4_]·H_2_O; Table S15. Torsion angles for (9) [Tb_2_(Qdca)_3_(H_2_O)_4_]·H_2_O; Table S16. Torsion angles for (13) [Er_2_(Qdca)_3_(H_2_O)_4_]·4H_2_O; Table S17. Hydrogen bonds of (1) [Nd_2_(Qdca)_3_(H_2_O)_3_]; Table S18. Hydrogen bonds of (4) [Nd_2_(Qdca)_3_(H_2_O)_4_]·3H_2_O; Table S19. Hydrogen bonds of (8) [Eu_2_(Qdca)_3_(H_2_O)_4_]·H2O; Table S20. Hydrogen bonds of (9) [Tb_2_(Qdca)_3_(H_2_O)_4_]·H_2_O; Table S21. Hydrogen bonds of (13) [Er_2_(Qdca)_3_(H_2_O)_4_]·4H_2_O; Table S22. Conformation of the COO− groups of the quionoline-2,4-dicarboxylate ligand; Table S23. Dihedral angles between best planes of carboxylic groups and quinolone ring; Table S24. Data of thermogravimetric analysis; Table S25. The main crystallographic data for [Gd_2_(Qdca)_3_(H_2_O)_4_]·H_2_O.

## References

[1] Seidi F., Jouyandeh M., Taghizadeh M., Taghizadeh A., Vahabi H., Habibzadeh S., Formela K., Saeb M.R. (2020). Metal-Organic Framework (MOF)/Epoxy Coatings: A Review. Materials.

[2] Łyszczek R., Rusinek I., Sienkiewicz-Gromiuk J., Iwan M., Pavlyuk O. (2019). 3-D Lanthanide Coordination Polymers with the Flexible 1,3-Phenylenediacetate Linker: Spectroscopic, Structural and Thermal Investigations. Polyhedron.

[3] Gustafsson M., Bartoszewicz A., Martín-Matute B., Sun J., Grins J., Zhao T., Li Z., Zhu G., Zou X. (2010). A Family of Highly Stable Lanthanide Metal−Organic Frameworks: Structural Evolution and Catalytic Activity. Chem. Mater..

[4] Gao Y., Gong S.-Y., Chen B., Xing W.-H., Fei Y.-F., Hu Z.-T., Pan Z. (2022). Progress in Metal-Organic Framework Catalysts for Selective Catalytic Reduction of NOx: A Mini-Review. Atmosphere.

[5] Liu J.-Q., Luo Z.-D., Pan Y., Kumar Singh A., Trivedi M., Kumar A. (2020). Recent Developments in Luminescent Coordination Polymers: Designing Strategies, Sensing Application and Theoretical Evidences. Coord. Chem. Rev..

[6] Liu X., Wang X., Kapteijn F. (2020). Water and Metal–Organic Frameworks: From Interaction toward Utilization. Chem. Rev..

[7] Wu D., Navrotsky A. (2015). Thermodynamics of Metal-Organic Frameworks. J. Solid State Chem..

[8] Seetharaj R., Vandana P.V., Arya P., Mathew S. (2019). Dependence of Solvents, PH, Molar Ratio and Temperature in Tuning Metal Organic Framework Architecture. Arab. J. Chem..

[9] Pan L., Frydel T., Sander M.B., Huang X., Li J. (2001). The Effect of PH on the Dimensionality of Coordination Polymers. Inorg. Chem..

[10] Lee Y.-R., Kim J., Ahn W.-S. (2013). Synthesis of Metal-Organic Frameworks: A Mini Review. Korean J. Chem. Eng..

[11] Stock N., Biswas S. (2012). Synthesis of Metal-Organic Frameworks (MOFs): Routes to Various MOF Topologies, Morphologies, and Composites. Chem. Rev..

[12] Rubio-Martinez M., Avci-Camur C., Thornton A.W., Imaz I., Maspoch D., Hill M.R. (2017). New Synthetic Routes towards MOF Production at Scale. Chem. Soc. Rev..

[13] Zhang P., Kang X., Tao L., Zheng L., Xiang J., Duan R., Li J., Chen P., Xing X., Mo G. (2022). A New Route for the Rapid Synthesis of Metal–Organic Frameworks at Room Temperature. CCS Chem..

[14] Ehrling S., Senkovska I., Efimova A., Bon V., Abylgazina L., Petkov P., Evans J.D., Gamal Attallah A., Wharmby M.T., Roslova M. (2022). Temperature Driven Transformation of the Flexible Metal–Organic Framework DUT-8(Ni). Chem. Eur. J..

[15] Khoshhal S., Ghoreyshi A.A., Jahanshahi M., Mohammadi M. (2015). Study of the Temperature and Solvent Content Effects on the Structure of Cu–BTC Metal Organic Framework for Hydrogen Storage. RSC Adv..

[16] Krause S., Bon V., Du H., Dunin-Borkowski R.E., Stoeck U., Senkovska I., Kaskel S. (2019). The Impact of Crystal Size and Temperature on the Adsorption-Induced Flexibility of the Zr-Based Metal–Organic Framework DUT-98. Beilstein J. Nanotechnol..

[17] Tanaka H., Ohsaki S., Hiraide S., Yamamoto D., Watanabe S., Miyahara M.T. (2014). Adsorption-Induced Structural Transition of ZIF-8: A Combined Experimental and Simulation Study. J. Phys. Chem. C.

[18] Gallaba D.H., Albesa A.G., Migone A.D. (2016). Evidence of Gate-Opening on Xenon Adsorption on ZIF-8: An Adsorption and Computer Simulation Study. J. Phys. Chem. C.

[19] Zhang C., Gee J.A., Sholl D.S., Lively R.P. (2014). Crystal-Size-Dependent Structural Transitions in Nanoporous Crystals: Adsorption-Induced Transitions in ZIF-8. J. Phys. Chem. C.

[20] Yang F., Mu H., Wang C., Xiang L., Yao K.X., Liu L., Yang Y., Han Y., Li Y., Pan Y. (2018). Morphological Map of ZIF-8 Crystals with Five Distinctive Shapes: Feature of Filler in Mixed-Matrix Membranes on C_3_H_6_/C_3_H_8_ Separation. Chem. Mater..

[21] Kavoosi N., Bon V., Senkovska I., Krause S., Atzori C., Bonino F., Pallmann J., Paasch S., Brunner E., Kaskel S. (2017). Tailoring Adsorption Induced Phase Transitions in the Pillared-Layer Type Metal–Organic Framework DUT-8(Ni). Dalton Trans..

[22] Miura H., Bon V., Senkovska I., Ehrling S., Watanabe S., Ohba M., Kaskel S. (2017). Tuning the Gate-Opening Pressure and Particle Size Distribution of the Switchable Metal–Organic Framework DUT-8(Ni) by Controlled Nucleation in a Micromixer. Dalton Trans..

[23] Xia Q.-H., Guo Z.-F., Liu L., Wang Z., Li B. (2012). *Catena*-Poly[[[Diaquacopper(II)]-μ-Quinoline-2,3-Dicarboxylato-κ^3^*N*,*O*^2^:*O*^3^] Monohydrate]. Acta Crystallogr. E Struct. Rep. Online.

[24] Li X.-L., Liu G.-Z. (2010). Crystal Structure of Bis(Hydrogen 2,3-Quinolinedicarboxylato)Copper(II), Cu(C_11_H_6_NO_4_)_2_. Z. Für Krist. New Cryst. Struct..

[25] Gong Y., Zhang M.M., Qin J.B., Li J., Meng J.P., Lin J.H. (2014). Metal(II) Complexes Synthesized Based on Quinoline-2,3-Dicarboxylate as Electrocatalysts for the Degradation of Methyl Orange. Dalton Trans..

[26] Hu M.-Y., He Q., Fan S.-J., Wang Z.-C., Liu L.-Y., Mu Y.-J., Peng Q., Zhu S.-F. (2018). Ligands with 1,10-Phenanthroline Scaffold for Highly Regioselective Iron-Catalyzed Alkene Hydrosilylation. Nat. Commun..

[27] Awad D.J., Conrad F., Koch A., Schilde U., Pöppl A., Strauch P. (2010). 1,10-Phenanthroline-Dithiolate Mixed Ligand Transition Metal Complexes. Synthesis, Characterization and EPR Spectroscopy. Inorganica Chim. Acta.

[28] Biradha K., Sarkar M., Rajput L. (2006). Crystal Engineering of Coordination Polymers Using 4,4′-Bipyridine as a Bond between Transition Metal Atoms. Chem. Commun..

[29] Jia J., Blake A.J., Champness N.R., Hubberstey P., Wilson C., Schröder M. (2008). Multi-Dimensional Transition-Metal Coordination Polymers of 4,4′-Bipyridine-*N*,*N*′-Dioxide: 1D Chains and 2D Sheets. Inorg. Chem..

[30] Seidel R.W., Goddard R., Zibrowius B., Oppel I.M. (2011). A Molecular Antenna Coordination Polymer from Cadmium(II) and 4,4’-Bipyridine Featuring Three Distinct Polymer Strands in the Crystal. Polymers.

[31] Irwin M., Doyle L.R., Krämer T., Herchel R., McGrady J.E., Goicoechea J.M. (2012). A Homologous Series of First-Row Transition-Metal Complexes of 2,2′-Bipyridine and Their Ligand Radical Derivatives: Trends in Structure, Magnetism, and Bonding. Inorg. Chem..

[32] Constable E.C., Housecroft C.E. (2019). The Early Years of 2,2’-Bipyridine—A Ligand in Its Own Lifetime. Molecules.

[33] Bünzli J.C.G., Choppin G.R. (1989). Lanthanide Probes in Life, Chemical and Earth Sciences.

[34] Yu X., Ryadun A.A., Pavlov D.I., Guselnikova T.Y., Potapov A.S., Fedin V.P. (2023). Highly Luminescent Lanthanide Metal-Organic Frameworks with Tunable Color for Nanomolar Detection of Iron(III), Ofloxacin and Gossypol and Anti-counterfeiting Applications. Angew. Chem. Int. Ed..

[35] Yu X., Ryadun A.A., Kovalenko K.A., Guselnikova T.Y., Ponomareva V.G., Potapov A.S., Fedin V.P. (2023). 4 in 1: Multifunctional Europium–Organic Frameworks with Luminescence Sensing Properties, White Light Emission, Proton Conductivity and Reverse Acetylene–Carbon Dioxide Adsorption Selectivity. Dalton Trans..

[36] Ivanova E.A., Smirnova K.S., Pozdnyakov I.P., Potapov A.S., Lider E.V. (2023). Synthesis, Crystal Structures, and Luminescence Properties of Lanthanide(III) Complexes with 1-(1H-Benzimidazol-1yl-Methyl)-1H-Benzotriazole. Inorganica Chim. Acta.

[37] Ivanova E.A., Smirnova K.S., Pozdnyakov I.P., Potapov A.S., Lider E.V. (2023). Photoluminescent Lanthanide(III) Coordination Polymers with Bis(1,2,4-Triazol-1-Yl)Methane Linker. Inorganics.

[38] Lis S., Elbanowski M., Mąkowska B., Hnatejko Z. (2002). Energy Transfer in Solution of Lanthanide Complexes. J. Photochem. Photobiol. A Chem..

[39] Kłonkowski A.M., Lis S., Pietraszkiewicz M., Hnatejko Z., Czarnobaj K., Elbanowski M. (2003). Luminescence Properties of Materials with Eu(III) Complexes: Role of Ligand, Coligand, Anion, and Matrix. Chem. Mater..

[40] Eliseeva S.V., Bünzli J.-C.G. (2010). Lanthanide Luminescence for Functional Materials and Bio-Sciences. Chem. Soc. Rev..

[41] Weissleder R., Ntziachristos V. (2003). Shedding Light onto Live Molecular Targets. Nat Med.

[42] Lenaerts P., Driesen K., Van Deun R., Binnemans K. (2005). Covalent Coupling of Luminescent Tris(2-Thenoyltrifluoroacetonato)Lanthanide(III) Complexes on a Merrifield Resin. Chem. Mater..

[43] Hasegawa Y., Wada Y., Yanagida S. (2004). Strategies for the Design of Luminescent Lanthanide(III) Complexes and Their Photonic Applications. J. Photochem. Photobiol. C: Photochem. Rev..

[44] Chen L., Chen H., Bai G., Yang X., Xie H., Xu S. (2020). Near-Infrared Excitation and Emitting Thermometer Based on Nd^3+^ Doped Ytterbium Molybdate with Thermally Enhanced Emissions. J. Lumin..

[45] Bart S.C. (2023). What Is the “Lanthanide Contraction”?. Inorg. Chem..

[46] Sharma S.K. (2012). Comparative Vibrational Spectroscopic Studies of 7-Chloro-4-Hydroxy-3-Quinolinecarboxylic Acid Based on Density Functional Theory. IOSRJAP.

[47] Özel A.E., Büyükmurat Y., Akyüz S. (2001). Infrared-Spectra and Normal-Coordinate Analysis of Quinoline and Quinoline Complexes. J. Mol. Struct..

[48] Bünzli J.-C.G. (2015). On the Design of Highly Luminescent Lanthanide Complexes. Coord. Chem. Rev..

[49] Bünzli J.-C.G. (2010). Lanthanide Luminescence for Biomedical Analyses and Imaging. Chem. Rev..

[50] Howarth A.J., Liu Y., Li P., Li Z., Wang T.C., Hupp J.T., Farha O.K. (2016). Chemical, Thermal and Mechanical Stabilities of Metal–Organic Frameworks. Nat. Rev. Mater.

[51] Vlasyuk D., Łyszczek R. (2021). Effect of Different Synthesis Approaches on Structural and Thermal Properties of Lanthanide(III) Metal–Organic Frameworks Based on the 1H-Pyrazole-3,5-Dicarboxylate Linker. J. Inorg. Organomet. Polym..

[52] Łyszczek R., Vlasyuk D., Podkościelna B., Głuchowska H., Piramidowicz R., Jusza A. (2022). A Top-Down Approach and Thermal Characterization of Luminescent Hybrid BPA.DA-MMA@Ln2L3 Materials Based on Lanthanide(III) 1H-Pyrazole-3,5-Dicarboxylates. Materials.

[53] Głuchowska H., Łyszczek R., Jusza A., Piramidowicz R. (2022). Effect of N,N′-Dimethylformamide Solvent on Structure and Thermal Properties of Lanthanide(III) Complexes with Flexible Biphenyl-4,4′-Dioxydiacetic Acid. J. Therm. Anal. Calorim..

[54] Keene F.R., Szalda D.J., Wilson T.A. (1987). Mode of Coordination of Tris(2-Pyridyl)Methanol to Ruthenium(II): Synthetic, Spectral, and Structural Studies of the Bis(Ligand) Species. Inorg. Chem..

[55] Salaam J., N’Dala-Louika I., Balogh C., Suleimanov I., Pilet G., Veyre L., Camp C., Thieuleux C., Riobé F., Maury O. (2022). Tris-dipicolinate Lanthanide Complexes: Influence of the Second Hydration Sphere on the Solid-State Luminescence Properties. Eur. J. Inorg. Chem..

[56] Chen S., Fan R.-Q., Sun C.-F., Wang P., Yang Y.-L., Su Q., Mu Y. (2012). Synthesis, Structure, and Luminescent Properties of Lanthanide-Based Two-Dimensional and Three-Dimensional Metal–Organic Frameworks with 2,4′-Biphenyldicarboxylic Acid. Cryst. Growth Des..

[57] Gao Q., Zhang C.-Y., Gao W.-H., Wu Y., Xie Y.-B., Sun J.-H. (2009). Two Binuclear Lanthanide Complexes with 4-Quinoline Carboxylic Acid: Crystal Structures and Luminescent Properties. J. Coord. Chem..

[58] Yan B., Zhou B. (2005). Photophysical Properties of Dysprosium Complexes with Aromatic Carboxylic Acids by Molecular Spectroscopy. J. Photochem. Photobiol. A Chem..

[59] Latva M., Takalo H., Mukkala V.-M., Matachescu C., Rodríguez-Ubis J.C., Kankare J. (1997). Correlation between the Lowest Triplet State Energy Level of the Ligand and Lanthanide(III) Luminescence Quantum Yield. J. Lumin..

[60] Huskowska E., Turowska-Tyrk I., Legendziewicz J., Riehl J.P. (2002). The Structure and Spectroscopy of Lanthanide(iii) Complexes with 2,2′-Bipyridine-1,1′-Dioxide in Solution and in the Solid State: Effects of Ionic Size and Solvent on Photophysics, Ligand Structure and Coordination. New J. Chem..

[61] Aebischer A., Gumy F., Bünzli J.-C.G. (2009). Intrinsic Quantum Yields and Radiative Lifetimes of Lanthanide Tris(Dipicolinates). Phys. Chem. Chem. Phys..

[62] Kwiatek D., Kubicki M., Toliński T., Ferenc W., Lis S., Hnatejko Z. (2019). A Series of New Pyridine Carboxamide Complexes and Self-Assemblies with Tb(III), Eu(III), Zn(II), Cu(II) Ions and Their Luminescent and Magnetic Properties. J. Coord. Chem..

[63] Kimura T., Kato Y. (1998). Luminescence Study on the Inner-Sphere Hydration Number of Lanthanide(III) Ions in Concentrated Aqueous Salt Solutions in Fluid and Frozen States. J. Alloys Compd..

[64] Wong K.-L., Bünzli J.-C.G., Tanner P.A. (2020). Quantum Yield and Brightness. J. Lumin..

[65] Woźny P., Soler-Carracedo K., Stopikowska N., Martín I.R., Runowski M. (2023). Structure-Dependent Luminescence of Eu^3+^-Doped Strontium Vanadates Synthesized with Different V: Sr Ratios—Application in WLEDs and Ultra-Sensitive Optical Thermometry. J. Mater. Chem. C.

[66] Kolesnikov I.E., Kalinichev A.A., Kurochkin M.A., Golyeva E.V., Kolesnikov E.Y., Kurochkin A.V., Lähderanta E., Mikhailov M.D. (2017). YVO4:Nd3+ Nanophosphors as NIR-to-NIR Thermal Sensors in Wide Temperature Range. Sci. Rep..

[67] Manyum P., Rittisut W., Mool-am-kha P., Ekwongsa C., Wantana N., Ruangtaweep Y., Popanao M., Rujirawat S., Yimnirun R., Kidkhunthod P. (2023). Structural and Luminescence Investigations of Gd^3+^-Er^3+^ Doped in Lithium Aluminum Borate Glasses Using XANES and EXAFS Techniques. Radiat. Phys. Chem..

[68] (2016). CrysAlisPRO Software System.

[69] Sheldrick G.M. (2015). Crystal structure refinement with SHELXL. Acta Crystallogr. C Struct. Chem..

[70] Faruggia L.J. (1999). WinGX suite for small-molecule single-crystal crystallography. J. Appl. Crystallogr..

