# Supplementary material for: A Series of Novel 3D Coordination Polymers Based on the Quinoline-2,4-dicarboxylate Building Block and Lanthanide(III) Ions—Temperature Dependence Investigations
Source: Molecules. 2023 Aug 30;28(17):6360. doi: 10.3390/molecules28176360 (PMC10489008; doi:10.3390/molecules28176360)
Supplement: Supplementary file 1 [file molecules-28-06360-s001.zip › molecules-2541065-supplementary.pdf]

molecules

MDPI

Supplementary Materials

A Series of Novel 3D Coordination Polymers Based on the Quinoline-2,4-dicarboxylate Building Block and Lanthanide(III) Ions—Temperature Dependence Investigations

Dmytro Vlasyuk [1,*], Renata Łyszczek [1,*], Liliana Mazur [1], Agnieszka Pladzyk [2], Zbigniew Hnatejko [3] and Przemysław Woźny [3]

[1] Department of General and Coordination Chemistry and Crystallography, Faculty of Chemistry, Institute of Chemical Sciences, Maria Curie-Skłodowska University, M. C. Skłodowskiej Sq. 2, 20-031 Lublin, Poland; liliana.mazur@mail.umcs.pl

[2] Department of Inorganic Chemistry, Faculty of Chemistry, Gdańsk University of Technology, Narutowicza 11/12, 80-233 Gdańsk, Poland

[3] Department of Rare Earths, Faculty of Chemistry, Adam Mickiewicz University in Poznań, Uniwersytetu Poznańskiego 8, 61-614 Poznań, Poland; zbigniew.hnatejko@amu.edu.pl (Z.H.); przemyslaw.wozny@amu.edu.pl (P.W.)

* Correspondence: dmytro.vlasyuk@mail.umcs.pl (D.V.), renata.lyszczek@mail.umcs.pl (R.Ł.)

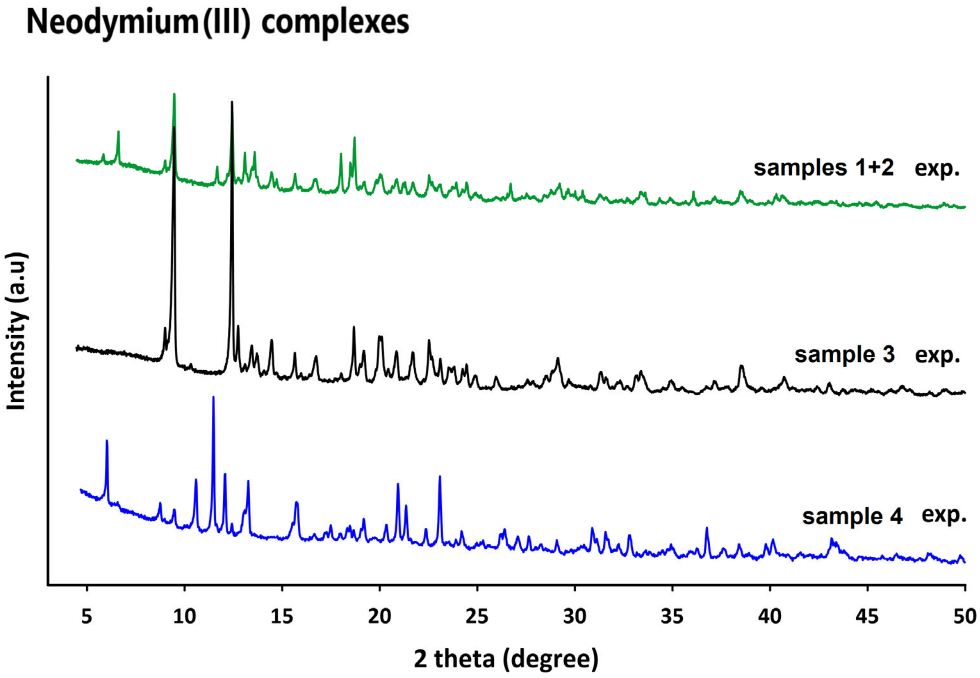

Figure S1. X-ray diffraction patterns of neodymium(III) samples obtained at different temperature (100, 120 and 150 °C).

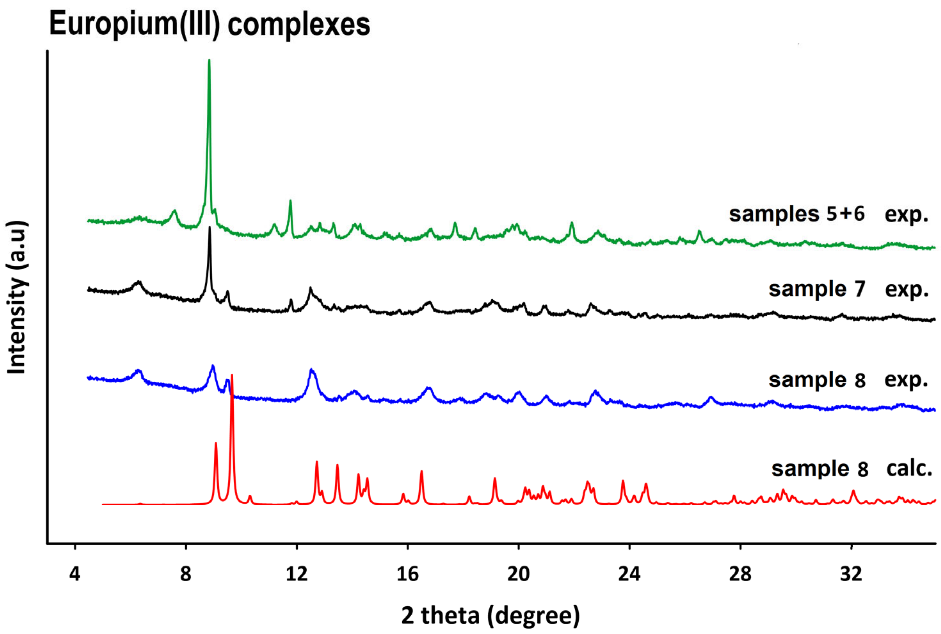

Figure S2. X-ray diffraction patterns of europium(III) samples obtained at different temperature (100, 120 and 150 °C).

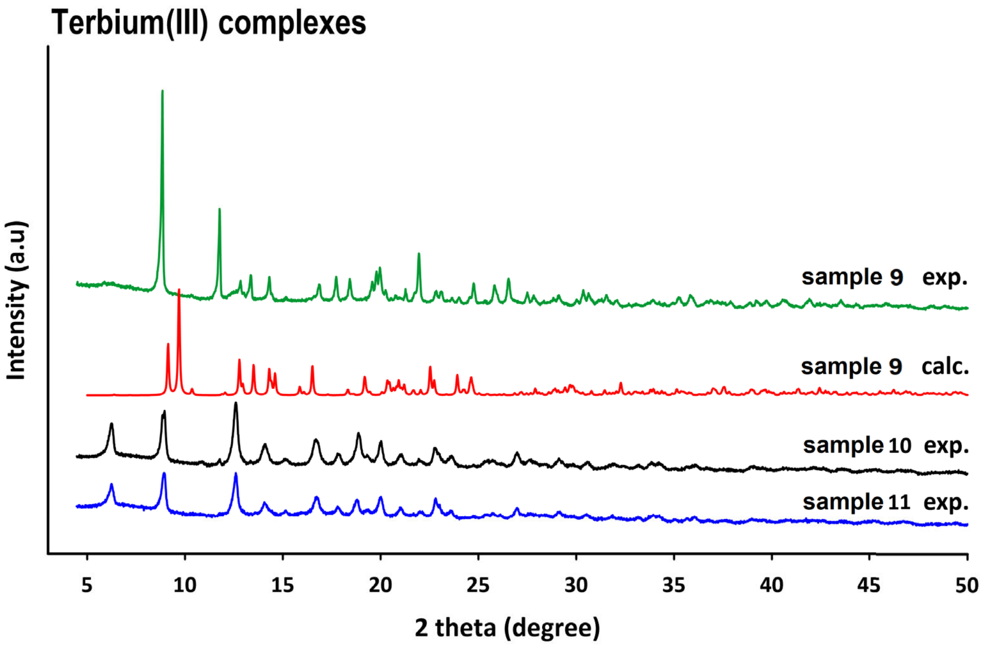

Figure S3. X-ray diffraction patterns of terbium(III) samples obtained at different temperature (100, 120 and 150 °C).

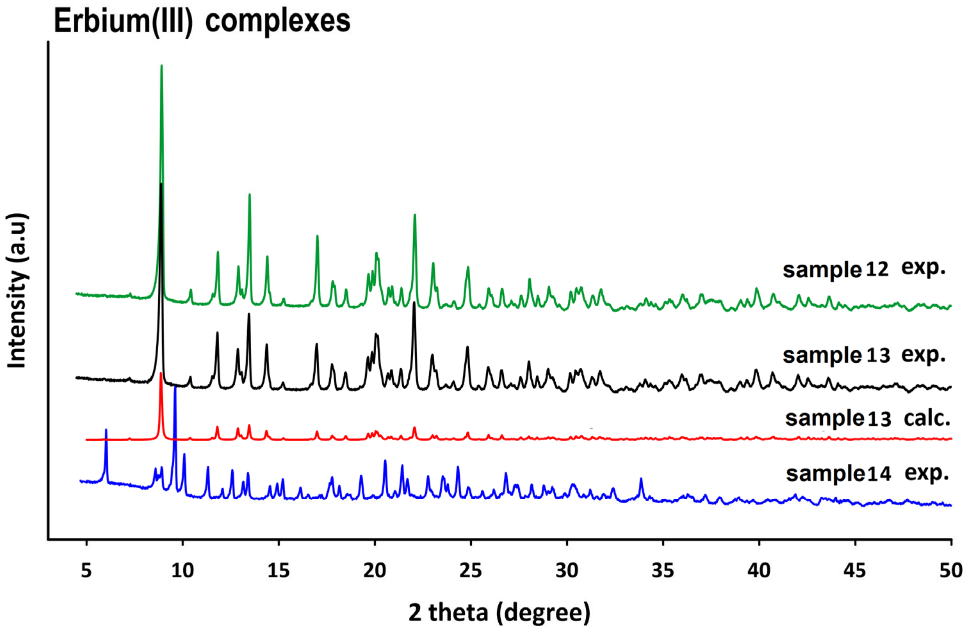

Figure S4. X-ray diffraction patterns of erbium(III) samples obtained at different temperature (100, 120 and 150 °C).

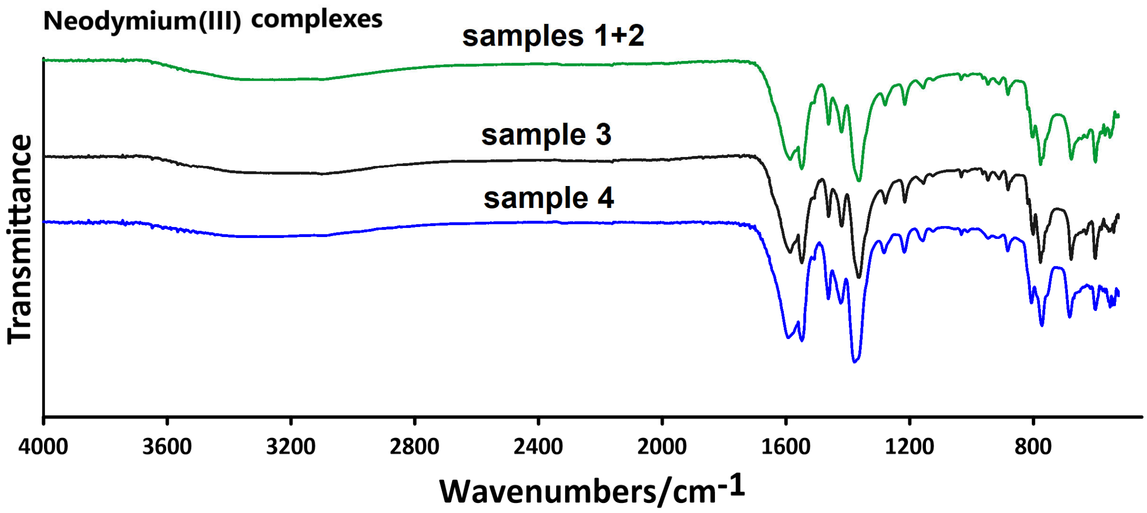

Figure S5. ATR/FTIR spectrum of neodymium(III) samples obtained at different temperature (100, 120 and 150 °C).

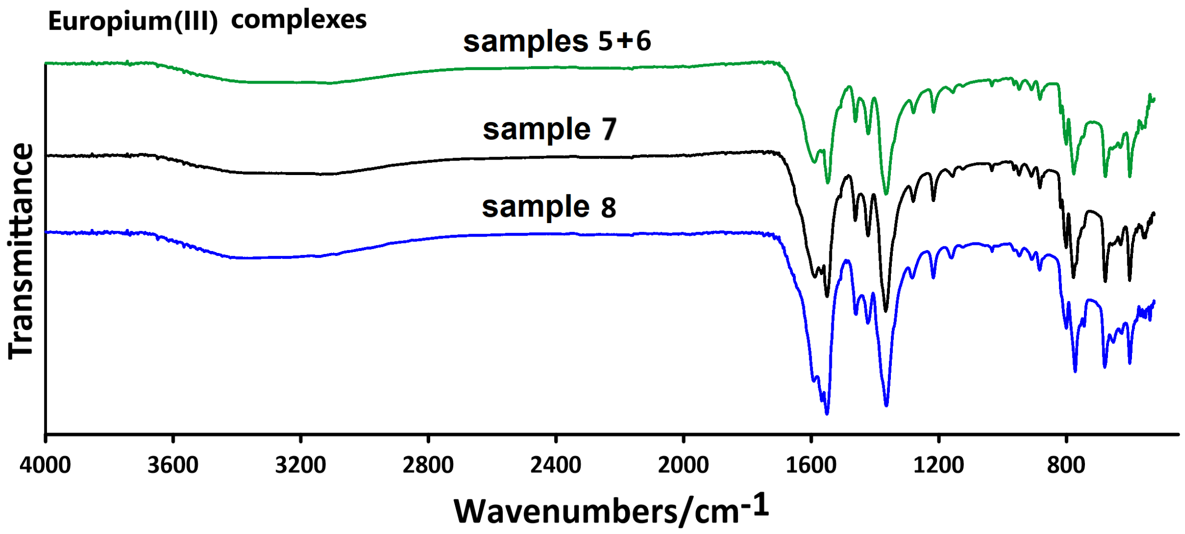

Figure S6. ATR/FTIR spectrum of europium(III) samples obtained at different temperature (100, 120 and 150 °C).

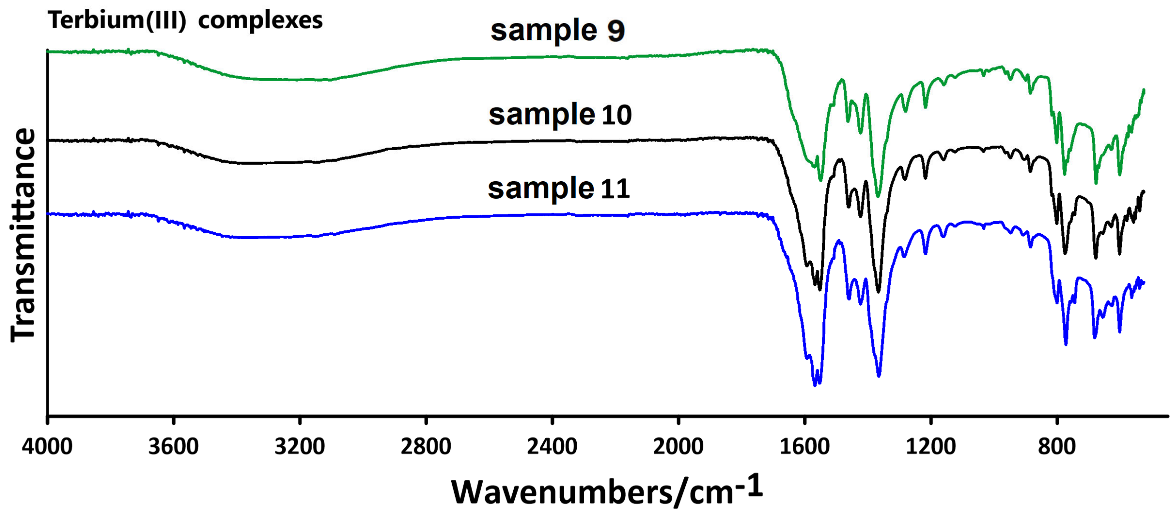

Figure S7. ATR/FTIR spectrum of terbium(III) samples obtained at different temperature (100, 120 and 150 °C).

Erbium(III) complexes

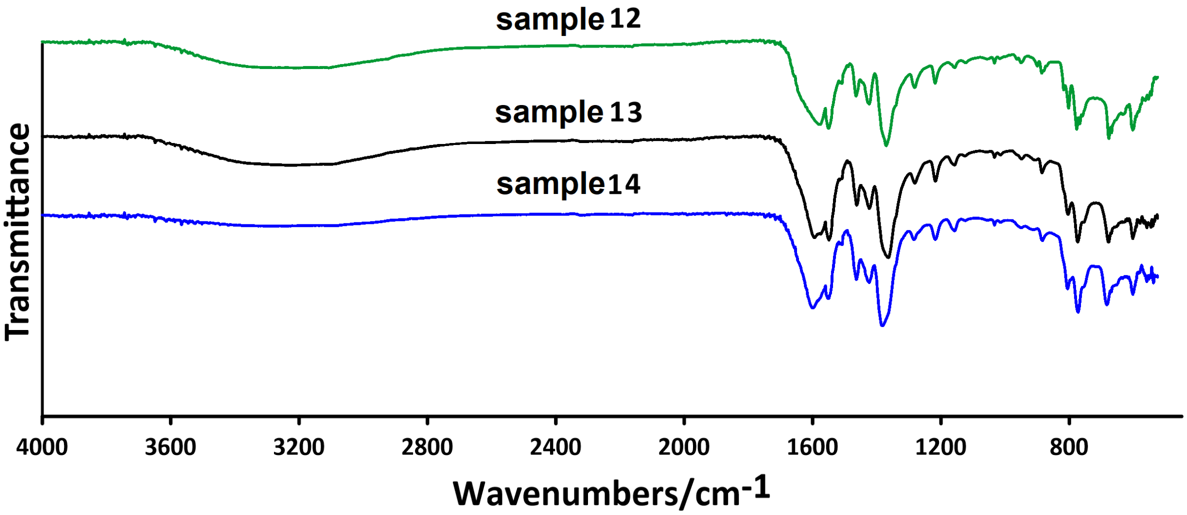

Figure S8. ATR/FTIR spectrum of erbium (III) samples obtained at different temperature (100, 120 and 150 °C).

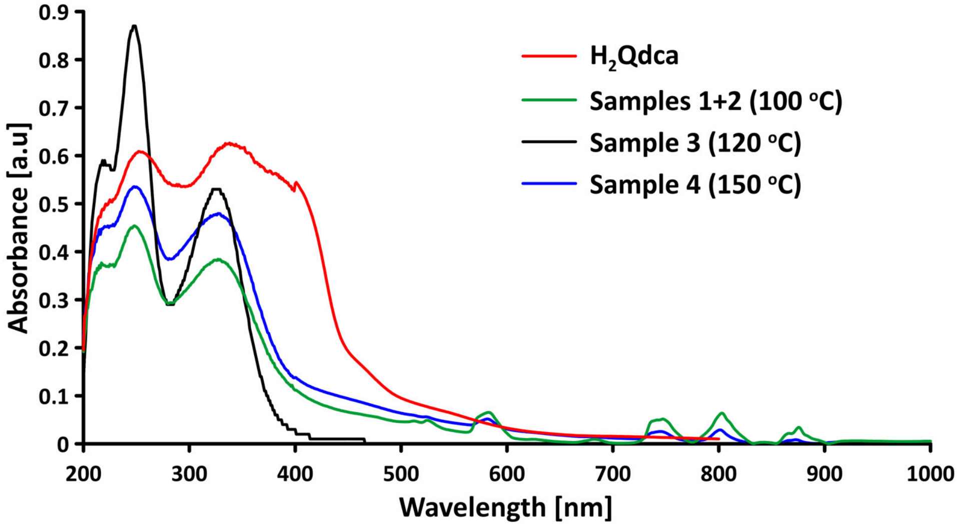

Figure S9. UV-VIS spectra of the neodymium(III) samples obtained at different temperature (100, 120 and 150 °C).

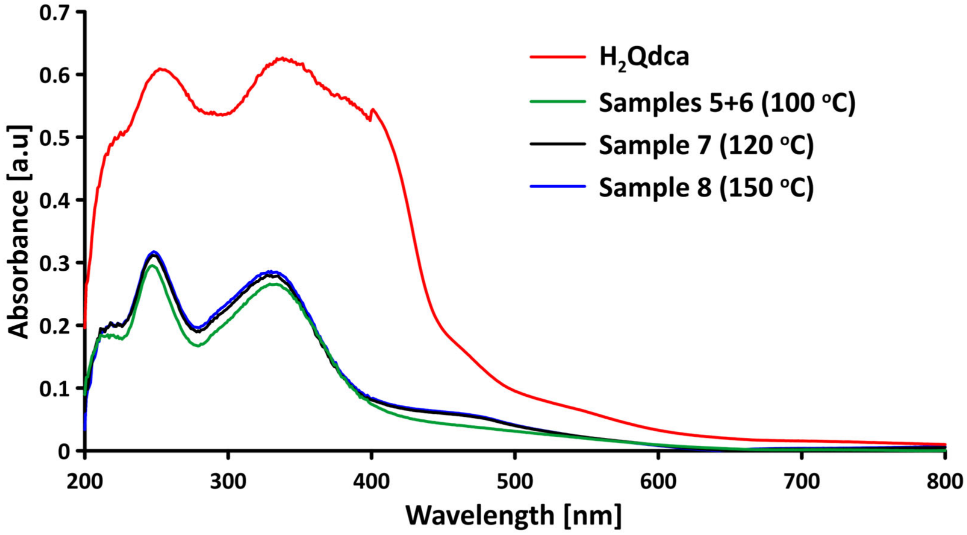

Figure S10. UV-VIS spectra of the europium(III) samples obtained at different temperature (100, 120 and 150 °C).

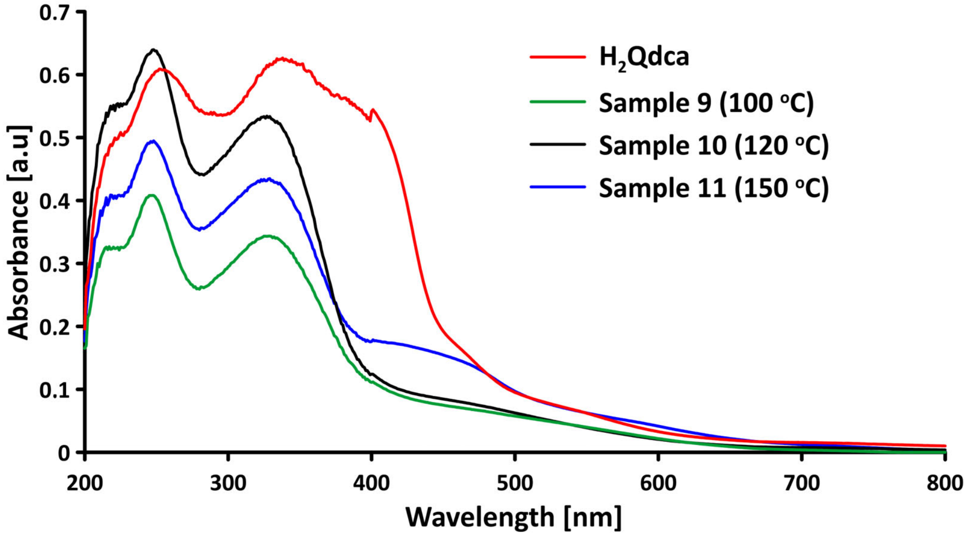

Figure S11. UV-VIS spectra of the terbium (III) samples obtained at different temperature (100, 120 and 150 °C).

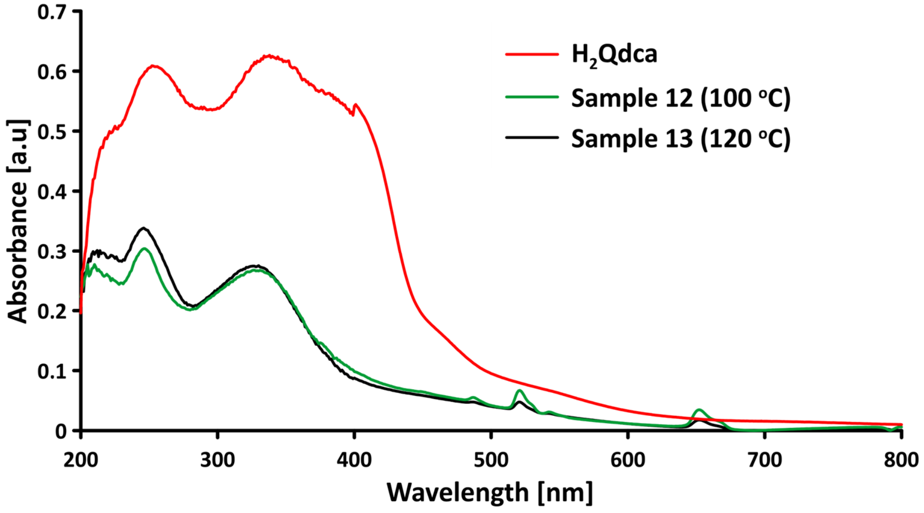

Figure S12. UV-VIS spectra of the erbium (III) samples obtained at different temperature (100 and 120 °C).

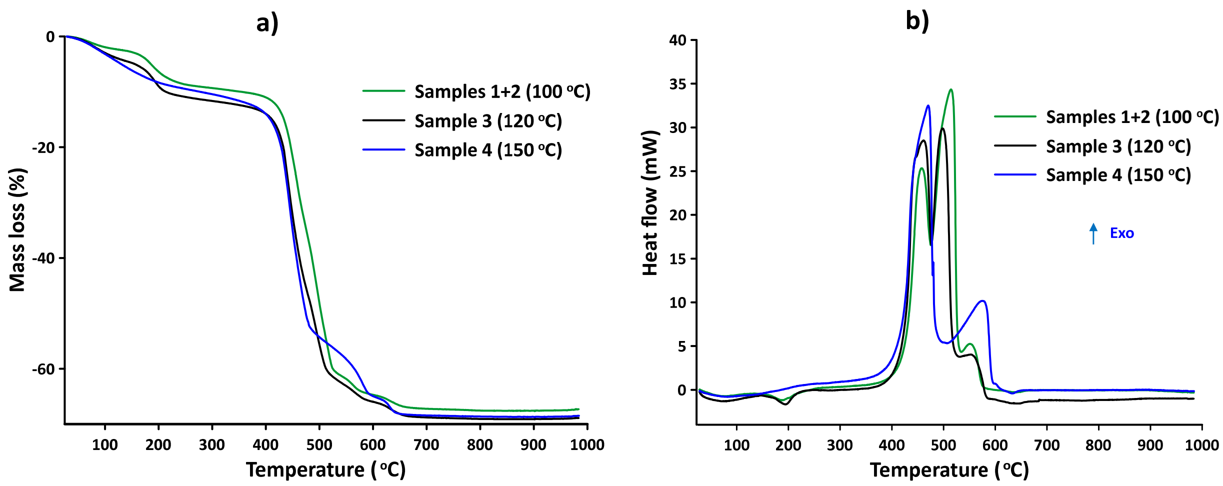

Figure S13. a) TG and b) DSC curves of neodymium (III) complexes in air atmosphere, obtained at different temperature (100, 120 and 150 °C).

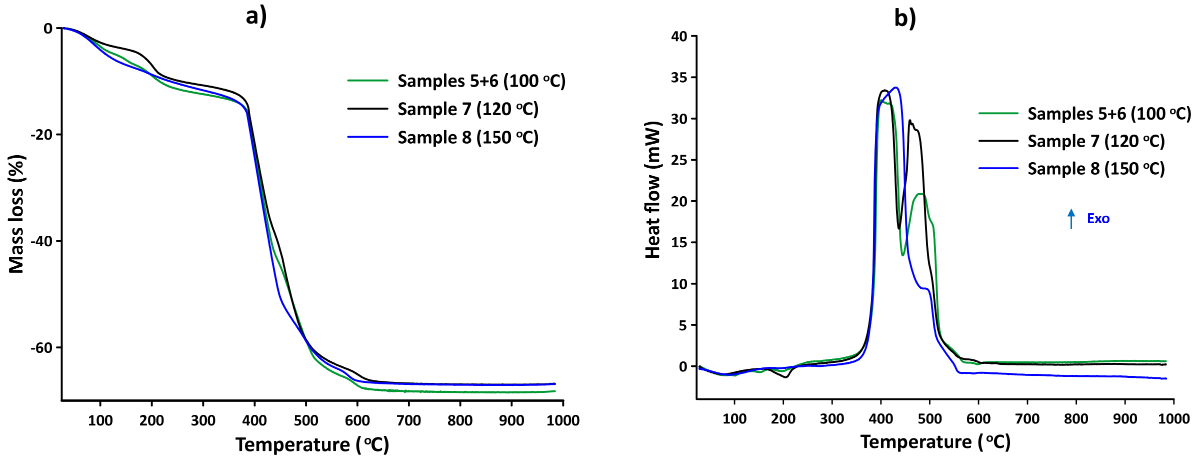

Figure S14. a) TG and b) DSC curves of europium (III) complexes in air atmosphere, obtained at different temperature (100, 120 and 150 °C).

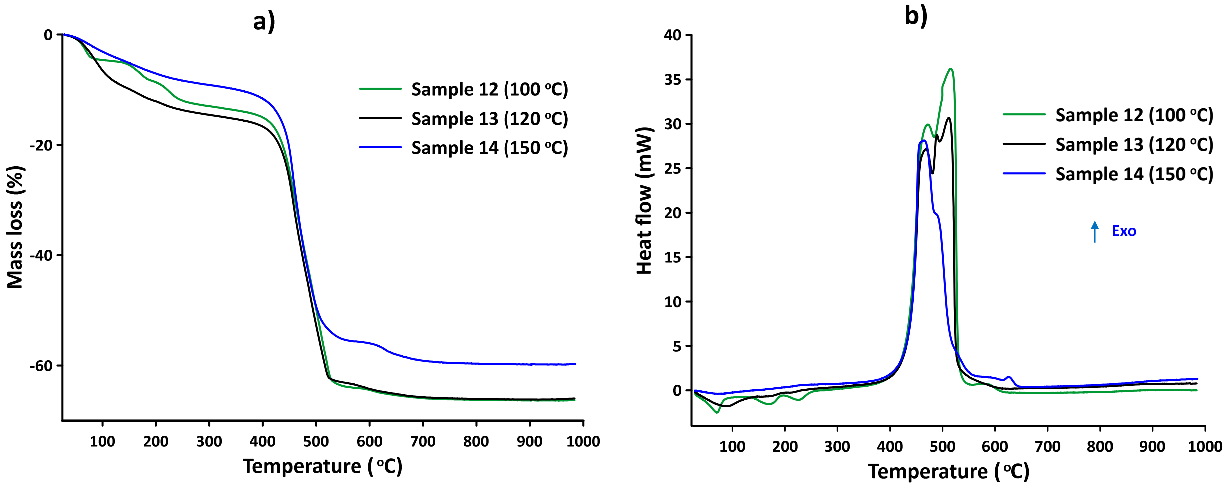

Figure S15. a) TG and b) DSC curves of erbium (III) complexes in air atmosphere, obtained at different temperature (100, 120 and 150 °C).

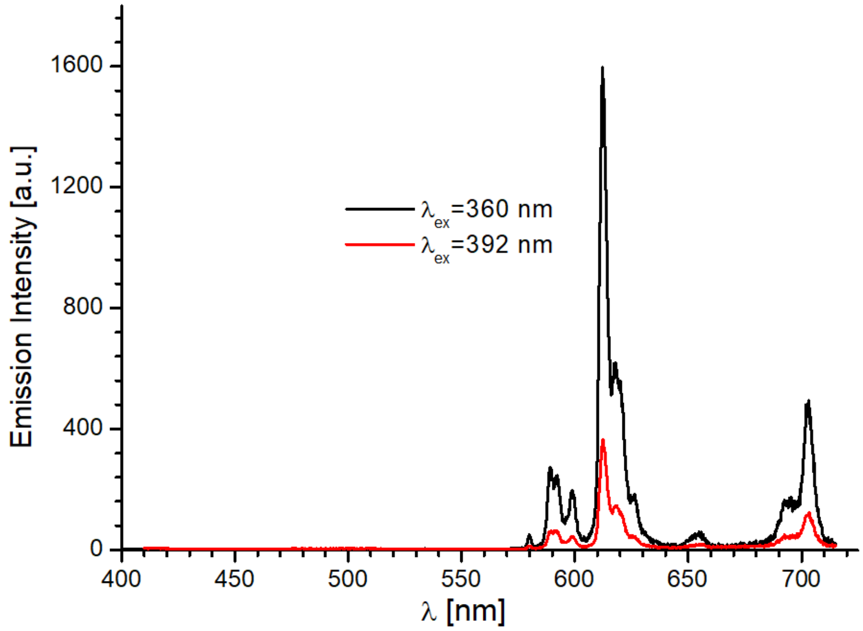

Figure S16. Room temperature emission spectra of $[Eu_2Qdca_3(H_2O)_4]{\bullet}H_2O$ in solid state (λ_{ex}= 360 and 392 nm).

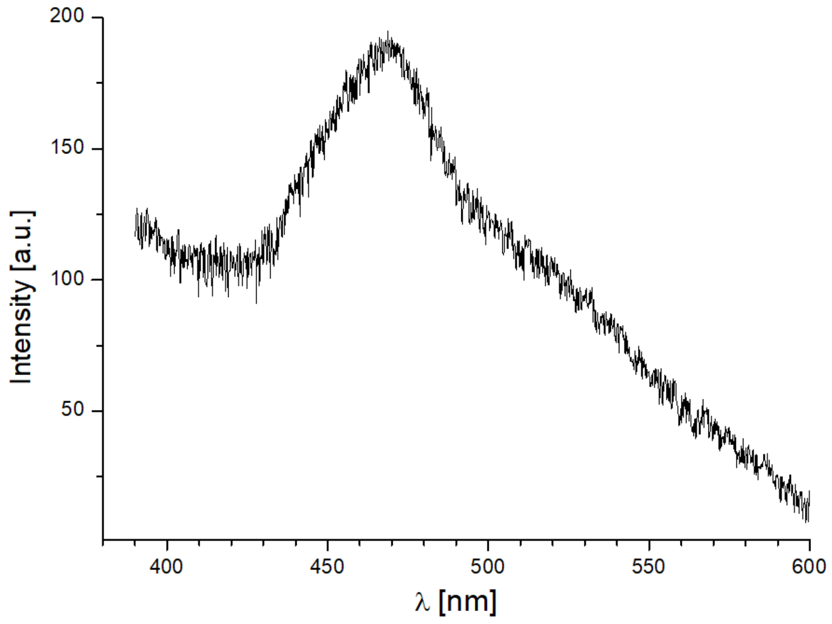

Figure S17. Low temperature phosphorescence spectrum of $[Gd_2(Qdca)_3(H_2O)_4] \bullet H_2O$ in ethanol solution at 148 °C (λ_{ex}=360 nm).

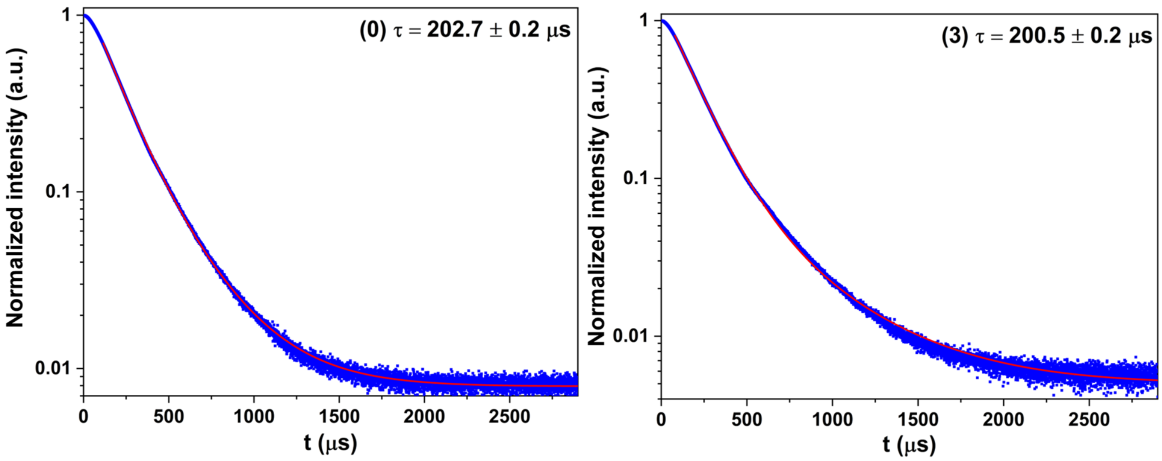

Figure S18. Typical luminescence decay profiles observed for Eu(III) complexes in the solid state at room temperature prepared in 100 °C and 150 °C (blue points are the raw data while the red line is a monoexponential fit).

Table S1. Results of the elemental analysis of prepared complexes.

Name of Complex	Temperature of Synthesis	Number of Compounds	C (%)		H (%)		N (%)	
			Cal.	Exp.	Cal.	Exp.	Cal.	Exp.
**Neodymium complexes**	**100 °C**	1+2	40.10[(1)]<br>38.69[(2)]	35.76	2.12[(1)]<br>2.44[(2)]	3.08	4.25[(1)]<br>4.10[(2)]	3.84
	**120 °C**	3	38.69	36.84	2.44	2.42	4.10	3.85
	**150 °C**	4	37.37	37.22	2.74	2.40	3.96	3.87
**Europium complexes**	**100 °C**	5+6	36.83[(5)]<br>38.11[(6)]	34.69	2.69[(5)]<br>2.41[(6)]	2.94	3.91[(5)]<br>3.91[(6)]	3.83
	**120 °C**	7	38.11	34.11	2.41	2.92	3.91	3.93
	**150 °C**	8	38.11	34.55	2.41	2.67	3.91	3.88
**Terbium complexes**	**100 °C**	9	37.61	35.77	2.37	2.96	3.99	3.92
	**120 °C**	10	36.36	34.61	2.66	2.95	3.86	4.06

	**150 °C**	11	36.36	34.25	2.66	2.76	3.86	4.07
**Erbium complexes**	**100 °C**	12	35.24	33.95	2.76	2.82	3.74	3.74
	**120 °C**	13	35.24	33.63	2.76	2.85	3.74	3.84
	**150 °C**	14	32.15	28.74	3.49	2.38	3.41	3.74

Table S2. Bond Lengths for (1)[$Nd_2(Qdca)_3(H_2O)_3$].

Atom	Atom	Length/Å	Atom	Atom	Length/Å
C2A	C3A	1.417(7)	C9B	C10B	1.420(8)
C2A	C11A	1.507(8)	C9C	C10C	1.425(9)
C2A	N1A	1.320(7)	C10A	N1A	1.374(7)
C2B	C3B	1.415(8)	C10B	N1B	1.350(8)
C2B	C11B	1.530(8)	C10C	N1C	1.374(8)
C2B	N1B	1.315(8)	C11A	O1A	1.269(7)
C2C	C3C	1.398(8)	C11A	O2A	1.240(7)
C2C	C11C	1.509(8)	C11B	O1B	1.254(8)
C2C	N1C	1.331(7)	C11B	O2B	1.270(8)
C3A	C4A	1.361(8)	C11C	O1C	1.251(7)
C3B	C4B	1.365(9)	C11C	O2C	1.250(8)
C3C	C4C	1.353(9)	C12A	O3A	1.256(10)
C4A	C5A	1.412(7)	C12A	O4A	1.239(10)
C4A	C12A	1.517(7)	C12B	O3B	1.251(11)
C4B	C5B	1.422(8)	C12B	O4B	1.243(11)
C4B	C12B	1.524(8)	C12C	O3C	1.250(7)
C4C	C5C	1.421(9)	C12C	O4C	1.255(7)
C4C	C12C	1.519(7)	C12C	Nd1	2.883(5)
C5A	C6A	1.427(8)	N1A	Nd1	2.664(4)
C5A	C10A	1.412(8)	N1C	Nd2[1]	2.888(4)
C5B	C6B	1.411(8)	O1A	Nd1	2.414(5)
C5B	C10B	1.427(8)	O1B	Nd2[2]	2.581(5)
C5C	C6C	1.432(9)	O1C	Nd2[1]	2.358(4)
C5C	C10C	1.428(8)	O2A	Nd2[2]	2.476(4)
C6A	C7A	1.355(9)	O1W	Nd1	2.449(6)
C6B	C7B	1.378(9)	O2B	Nd1	2.409(5)
C6C	C7C	1.353(10)	O2W	Nd2	2.433(5)
C7A	C8A	1.404(9)	O3A	Nd1[3]	2.464(5)
C7B	C8B	1.414(10)	O3B	Nd2	2.355(5)
C7C	C8C	1.411(10)	O3C	Nd1	2.534(4)
C8A	C9A	1.359(8)	O3W	Nd2	2.438(5)
C8B	C9B	1.357(9)	O4A	Nd1[4]	2.425(6)
C8C	C9C	1.353(10)	O4B	Nd2[5]	2.469(5)
C9A	C10A	1.420(7)	O4C	Nd1	2.502(4)

Symmetry code: [1]1/2+x,3/2-y,+z; [2]-1/2+x,3/2-y,+z; [3]1-x,1-y,-1/2+z; [4]1-x,1-y,1/2+z; [5]+x,+y,-1+z.

Table S3. Bond Lengths for (4) [$Nd_2(Qdca)_3(H_2O)_4$]·$3H_2O$.

Atom	Atom	Length/Å	Atom	Atom	Length/Å
Nd1	O1A[1]	2.368(4)	C5B	C4B	1.415(9)
Nd1	O4A	2.360(5)	C5B	C6B	1.427(9)
Nd1	O2A[2]	2.508(5)	C10B	C9B	1.432(9)
Nd1	O2B[3]	2.381(4)	C4B	C3B	1.375(9)
Nd1	O4B	2.368(5)	C4B	C12B	1.504(8)
Nd1	N1A[1]	2.780(5)	C10A	C5A	1.421(9)
Nd1	O1W	2.501(6)	C10A	C9A	1.413(9)
Nd1	O4W	2.571(7)	C3B	C2B	1.411(9)
Nd2	O3B	2.354(4)	C4A	C5A	1.429(9)

Nd2	O1B[4]	2.402(4)	C4A	C3A	1.367(9)
Nd2	O1C[5]	2.400(5)	C4A	C12A	1.525(9)
Nd2	O4C	2.302(5)	C5A	C6A	1.414(9)
Nd2	O3W	2.484(6)	C2B	C11B	1.496(9)
Nd2	N1B[4]	2.724(5)	C5C	C6C	1.433(10)
Nd2	N1C[5]	2.727(5)	C5C	C10C	1.412(9)
Nd2	O2W	2.518(6)	C5C	C4C	1.409(9)
O1A	C11A	1.270(8)	C3A	C2A	1.399(9)
O3B	C12B	1.255(8)	O3C	C12C	1.212(9)
O4A	C12A	1.245(8)	C2A	C11A	1.503(9)
O2A	C11A	1.255(8)	C9B	C8B	1.359(10)
O1B	C11B	1.261(8)	C6B	C7B	1.352(10)
O2B	C11B	1.248(8)	C6C	C7C	1.342(10)
O4B	C12B	1.236(8)	C9A	C8A	1.353(11)
O1C	C11C	1.267(8)	C10C	C9C	1.407(9)
O4C	C12C	1.257(8)	C2C	C3C	1.403(9)
N1A	C10A	1.377(8)	C2C	C11C	1.505(10)
N1A	C2A	1.325(8)	C4C	C3C	1.371(10)
N1B	C10B	1.355(8)	C4C	C12C	1.524(9)
N1B	C2B	1.335(8)	C9C	C8C	1.351(11)
O3A	C12A	1.240(9)	C6A	C7A	1.372(11)
O2C	C11C	1.251(8)	C7A	C8A	1.400(11)
N1C	C10C	1.382(9)	C8B	C7B	1.390(10)
N1C	C2C	1.322(8)	C8C	C7C	1.424(10)
C5B	C10B	1.434(8)			

Symmetry code: [1]2-x,2-y,1-z; [2]-1+x,+y,+z; [3]1-x,2-y,1-z; [4]1-x,1-y,1-z; [5]-x,1-y,-z.

Table S4. Bond Lengths for (8) $[Eu_2(Qdca)_3(H_2O)_4]\cdot H_2O$.

Atom	Atom	Length/Å	Atom	Atom	Length/Å
Eu1	N1A	2.830(4)	C4C	C12C	1.527(7)
Eu1	N1B[1]	2.775(4)	C5A	C6A	1.412(8)
Eu1	O1A	2.287(4)	C5A	C10A	1.420(7)
Eu1	O1B[1]	2.374(3)	C5B	C6B	1.419(8)
Eu1	O3A[2]	2.367(4)	C5B	C10B	1.425(8)
Eu1	O4A[3]	2.301(4)	C5C	C6C	1.417(9)
Eu1	O4B	2.296(4)	C5C	C10C	1.427(7)
Eu1	O4W	2.454(4)	C6A	C7A	1.377(9)
Eu2	C11C	3.224(5)	C6B	C7B	1.364(9)
Eu2	N1C	2.689(5)	C6C	C7C	1.338(9)
Eu2	O1C	2.280(4)	C7A	C8A	1.410(9)
Eu2	O1W	2.473(5)	C7B	C8B	1.395(9)
Eu2	O2B	2.424(4)	C7C	C8C	1.406(9)
Eu2	O2W	2.458(5)	C8A	C9A	1.357(9)
Eu2	O3C[2]	2.376(4)	C8B	C9B	1.359(9)
Eu2	O3W	2.462(5)	C8C	C9C	1.355(9)
Eu2	O4C[4]	2.319(4)	C9A	C10A	1.412(8)
C2A	C3A	1.403(7)	C9B	C10B	1.419(8)
C2A	C11A	1.496(8)	C9C	C10C	1.411(8)
C2A	N1A	1.328(7)	C10A	N1A	1.383(7)
C2B	C3B	1.410(8)	C10B	N1B	1.364(7)
C2B	C11B	1.499(8)	C10C	N1C	1.381(7)
C2B	N1B	1.330(7)	C11A	O1A	1.278(7)
C2C	C3C	1.412(7)	C11A	O2A	1.238(7)
C2C	C11C	1.492(8)	C11B	O1B	1.257(6)

C2C	N1C	1.327(7)	C11B	O2B	1.251(7)
C3A	C4A	1.362(8)	C11C	O1C	1.278(7)
C3B	C4B	1.369(8)	C11C	O2C	1.234(7)
C3C	C4C	1.354(8)	C12A	O3A	1.239(7)
C4A	C5A	1.413(8)	C12A	O4A	1.254(7)
C4A	C12A	1.533(7)	C12B	O3B	1.219(7)
C4B	C5B	1.421(7)	C12B	O4B	1.273(7)
C4B	C12B	1.510(8)	C12C	O3C	1.248(7)
C4C	C5C	1.421(8)	C12C	O4C	1.245(7)

Symmetry code: [1]2-x,1-y,1-z; [2]1+x,+y,+z; [3]1-x,-y,1-z; [4]1-x,1-y,2-z.

Table S5. Bond Lengths for (9) $[Tb_2(Qdca)_3(H_2O)_4]\cdot H_2O$.

Atom	Atom	Length/Å	Atom	Atom	Length/Å
C2A	C3A	1.416(8)	C9B	C10B	1.419(8)
C2A	C11A	1.518(8)	C9C	C10C	1.403(8)
C2A	N1A	1.322(7)	C10A	N1A	1.379(7)
C2B	C3B	1.406(8)	C10B	N1B	1.386(7)
C2B	C11B	1.499(8)	C10C	N1C	1.390(7)
C2B	N1B	1.321(7)	C11A	O1A	1.262(7)
C2C	C3C	1.423(7)	C11A	O2A	1.231(7)
C2C	C11C	1.511(8)	C11B	O1B	1.257(6)
C2C	N1C	1.305(7)	C11B	O2B	1.258(7)
C3A	C4A	1.345(8)	C11C	O1C	1.281(7)
C3B	C4B	1.361(8)	C11C	O2C	1.221(7)
C3C	C4C	1.360(8)	C12A	O3A	1.241(7)
C4A	C5A	1.434(8)	C12A	O4A	1.242(7)
C4A	C12A	1.530(7)	C12B	O3B	1.225(7)
C4B	C5B	1.431(8)	C12B	O4B	1.268(7)
C4B	C12B	1.524(8)	C12C	O3C	1.241(8)
C4C	C5C	1.426(8)	C12C	O4C	1.255(7)
C4C	C12C	1.528(8)	N1A	Tb1	2.817(5)
C5A	C6A	1.408(8)	N1B	Tb1[1]	2.751(5)
C5A	C10A	1.410(8)	N1C	Tb2	2.661(5)
C5B	C6B	1.416(8)	O1A	Tb1	2.269(4)
C5B	C10B	1.419(8)	O1B	Tb1[1]	2.349(4)
C5C	C6C	1.416(8)	O1C	Tb2	2.263(4)
C5C	C10C	1.419(8)	O1W	Tb2	2.421(4)
C6A	C7A	1.373(9)	O2B	Tb2	2.386(4)
C6B	C7B	1.343(9)	O2W	Tb2	2.431(5)
C6C	C7C	1.347(10)	O3A	Tb1[2]	2.340(4)
C7A	C8A	1.406(9)	O3C	Tb2[2]	2.351(4)
C7B	C8B	1.388(9)	O3W	Tb2	2.443(5)
C7C	C8C	1.406(10)	O4A	Tb1[3]	2.286(4)
C8A	C9A	1.351(9)	O4B	Tb1	2.263(4)
C8B	C9B	1.354(9)	O4C	Tb2[4]	2.290(4)
C8C	C9C	1.353(9)	O4W	Tb1	2.444(5)
C9A	C10A	1.431(8)			

Symmetry code: [1]2-x,1-y,1-z; [2]-1+x,+y,+z; [3]1-x,-y,1-z; [4]1-x,1-y,2-z.

Table S6. Bond Lengths for (13) $[Er_2(Qdca)_3(H_2O)_4]{\cdot}4H_2O$.

Atom	Atom	Length/Å	Atom	Atom	Length/Å
Er1	N1B[1]	2.715(3)	C5A	C6A	1.419(6)
Er1	O1A	2.236(3)	C5A	C10A	1.430(5)
Er1	O1B[1]	2.320(3)	C5B	C6B	1.425(6)
Er1	O1W	2.384(3)	C5B	C10B	1.422(5)
Er1	O3A[2]	2.313(3)	C5C	C6C	1.416(6)
Er1	O4A[3]	2.249(3)	C5C	C10C	1.413(5)
Er1	O4B	2.237(3)	C6A	C7A	1.356(7)
Er2	N1C	2.601(3)	C6B	C7B	1.350(6)
Er2	O1C	2.285(3)	C6C	C7C	1.358(7)
Er2	O2B	2.357(3)	C7A	C8A	1.412(7)
Er2	O2W	2.380(3)	C7B	C8B	1.406(6)
Er2	O3C[4]	2.296(3)	C7C	C8C	1.417(7)
Er2	O3W	2.357(3)	C8A	C9A	1.363(7)
Er2	O4C[2]	2.375(3)	C8B	C9B	1.361(6)
Er2	O4W	2.291(3)	C8C	C9C	1.360(6)
C2A	C3A	1.401(5)	C9A	C10A	1.409(6)
C2A	C11A	1.518(5)	C9B	C10B	1.417(5)
C2A	N1A	1.318(5)	C9C	C10C	1.415(6)
C2B	C3B	1.419(5)	C10A	N1A	1.374(5)
C2B	C11B	1.507(5)	C10B	N1B	1.365(5)
C2B	N1B	1.323(5)	C10C	N1C	1.375(5)
C2C	C3C	1.405(5)	C11A	O1A	1.273(5)
C2C	C11C	1.506(5)	C11A	O2A	1.226(5)
C2C	N1C	1.329(5)	C11B	O1B	1.249(4)
C3A	C4A	1.373(5)	C11B	O2B	1.262(5)
C3B	C4B	1.370(5)	C11C	O1C	1.258(5)
C3C	C4C	1.363(6)	C11C	O2C	1.238(5)
C4A	C5A	1.410(5)	C12A	O3A	1.242(5)
C4A	C12A	1.530(5)	C12A	O4A	1.237(5)
C4B	C5B	1.422(5)	C12B	O3B	1.232(5)
C4B	C12B	1.519(5)	C12B	O4B	1.247(5)
C4C	C5C	1.423(5)	C12C	O3C	1.269(5)
C4C	C12C	1.507(5)	C12C	O4C	1.232(5)

Symmetry code: [1]-x,1-y,1-z; [2]-1+x,+y,+z; [3]1-x,-y,1-z; [4]1-x,1-y,2-z.

Table S7. Bond Angles for (1) $[Nd_2(Qdca)_3(H_2O)_3]$.

Atom	Atom	Atom	Angle/°	Atom	Atom	Atom	Angle/°
C3A	C2A	C11A	121.6(5)	C2C	N1C	Nd2[1]	111.0(3)
N1A	C2A	C3A	123.0(5)	C10C	N1C	Nd2[1]	132.0(4)
N1A	C2A	C11A	115.4(5)	C11A	O1A	Nd1	125.9(4)
C3B	C2B	C11B	121.8(5)	C11B	O1B	Nd2[2]	158.6(5)
N1B	C2B	C3B	122.3(6)	C11C	O1C	Nd2[1]	132.2(4)
N1B	C2B	C11B	115.8(5)	C11A	O2A	Nd2[2]	116.4(3)
C3C	C2C	C11C	119.6(5)	C11B	O2B	Nd1	139.4(4)
N1C	C2C	C3C	123.7(5)	C12A	O3A	Nd1[3]	155.2(4)
N1C	C2C	C11C	116.7(5)	C12B	O3B	Nd2	154.4(5)
C4A	C3A	C2A	119.2(5)	C12C	O3C	Nd1	92.9(3)
C4B	C3B	C2B	118.9(5)	C12A	O4A	Nd1[4]	159.3(5)
C4C	C3C	C2C	120.8(6)	C12B	O4B	Nd2[5]	149.9(5)
C3A	C4A	C5A	119.2(5)	C12C	O4C	Nd1	94.3(3)
C3A	C4A	C12A	124.4(5)	N1A	Nd1	C12C	124.10(15)
C5A	C4A	C12A	116.4(4)	O1A	Nd1	C12C	170.29(19)

C3B	C4B	C5B	120.4(5)	O1A	Nd1	N1A	62.19(14)
C3B	C4B	C12B	120.5(5)	O1A	Nd1	O1W	77.1(2)
C5B	C4B	C12B	119.0(5)	O1A	Nd1	O3A[4]	90.36(18)
C3C	C4C	C5C	118.4(5)	O1A	Nd1	O3C	157.32(16)
C3C	C4C	C12C	117.7(5)	O1A	Nd1	O4A[3]	110.02(19)
C5C	C4C	C12C	123.8(5)	O1A	Nd1	O4C	148.51(14)
C4A	C5A	C6A	123.3(5)	O1W	Nd1	C12C	93.3(2)
C4A	C5A	C10A	118.5(5)	O1W	Nd1	N1A	125.1(2)
C10A	C5A	C6A	118.2(5)	O1W	Nd1	O3A[4]	69.51(19)
C4B	C5B	C10B	116.2(5)	O1W	Nd1	O3C	108.2(2)
C6B	C5B	C4B	124.9(5)	O1W	Nd1	O4C	77.6(2)
C6B	C5B	C10B	118.9(5)	O2B	Nd1	C12C	102.61(18)
C4C	C5C	C6C	124.1(6)	O2B	Nd1	N1A	119.39(18)
C4C	C5C	C10C	117.7(6)	O2B	Nd1	O1A	78.01(16)
C10C	C5C	C6C	118.1(6)	O2B	Nd1	O1W	82.9(2)
C7A	C6A	C5A	120.8(5)	O2B	Nd1	O3A[4]	151.86(19)
C7B	C6B	C5B	120.4(6)	O2B	Nd1	O3C	124.11(19)
C7C	C6C	C5C	120.5(6)	O2B	Nd1	O4A[3]	76.9(2)
C6A	C7A	C8A	120.1(5)	O2B	Nd1	O4C	80.54(16)
C6B	C7B	C8B	120.2(6)	O3A[4]	Nd1	C12C	84.7(2)
C6C	C7C	C8C	121.1(6)	O3A[4]	Nd1	N1A	75.2(2)
C9A	C8A	C7A	121.7(5)	O3A[4]	Nd1	O3C	71.9(2)
C9B	C8B	C7B	121.0(6)	O3A[4]	Nd1	O4C	98.03(19)
C9C	C8C	C7C	120.5(6)	O3C	Nd1	C12C	25.66(15)
C8A	C9A	C10A	119.2(5)	O3C	Nd1	N1A	98.77(13)
C8B	C9B	C10B	120.2(6)	O4A[3]	Nd1	C12C	79.4(2)
C8C	C9C	C10C	120.5(6)	O4A[3]	Nd1	N1A	76.4(2)
C5A	C10A	C9A	120.0(5)	O4A[3]	Nd1	O1W	156.3(2)
N1A	C10A	C5A	121.4(4)	O4A[3]	Nd1	O3A[4]	131.19(14)
N1A	C10A	C9A	118.6(5)	O4A[3]	Nd1	O3C	74.2(2)
C9B	C10B	C5B	119.3(6)	O4A[3]	Nd1	O4C	87.0(2)
N1B	C10B	C5B	122.2(5)	O4C	Nd1	C12C	25.72(16)
N1B	C10B	C9B	118.3(5)	O4C	Nd1	N1A	149.30(14)
C9C	C10C	C5C	119.2(6)	O4C	Nd1	O3C	51.37(14)
N1C	C10C	C5C	122.5(6)	O1B[1]	Nd2	N1C[2]	142.66(18)
N1C	C10C	C9C	118.3(6)	O1C[2]	Nd2	N1C[2]	61.18(14)
O1A	C11A	C2A	115.5(5)	O1C[2]	Nd2	O1B[1]	131.58(15)
O2A	C11A	C2A	121.1(5)	O1C[2]	Nd2	O2A[1]	143.23(15)
O2A	C11A	O1A	123.4(5)	O1C[2]	Nd2	O2W	66.01(15)
O1B	C11B	C2B	117.8(5)	O1C[2]	Nd2	O3W	89.76(18)
O1B	C11B	O2B	125.2(6)	O1C[2]	Nd2	O4B[6]	71.12(16)
O2B	C11B	C2B	117.0(5)	O2A[1]	Nd2	N1C[2]	85.63(13)
O1C	C11C	C2C	118.5(5)	O2A[1]	Nd2	O1B[1]	84.27(16)
O2C	C11C	C2C	117.8(5)	O2W	Nd2	N1C[2]	113.31(17)
O2C	C11C	O1C	123.7(6)	O2W	Nd2	O1B[1]	65.57(16)
O3A	C12A	C4A	116.7(7)	O2W	Nd2	O2A[1]	148.86(18)
O4A	C12A	C4A	117.3(8)	O2W	Nd2	O3W	78.8(2)
O4A	C12A	O3A	126.0(5)	O2W	Nd2	O4B[6]	126.54(19)
O3B	C12B	C4B	115.8(7)	O3B	Nd2	N1C[2]	71.95(19)
O4B	C12B	C4B	120.0(7)	O3B	Nd2	O1B[1]	71.71(18)
O4B	C12B	O3B	124.2(5)	O3B	Nd2	O1C[2]	96.27(17)
C4C	C12C	Nd1	177.7(5)	O3B	Nd2	O2A[1]	87.1(2)
O3C	C12C	C4C	119.9(5)	O3B	Nd2	O2W	76.6(2)
O3C	C12C	O4C	121.3(5)	O3B	Nd2	O3W	149.5(2)

O3C	C12C	Nd1	61.4(3)	O3B	Nd2	O4B[6]	139.57(18)
O4C	C12C	C4C	118.7(5)	O3W	Nd2	N1C[2]	135.36(19)
O4C	C12C	Nd1	60.0(3)	O3W	Nd2	O1B[1]	81.96(19)
C2A	N1A	C10A	118.6(4)	O3W	Nd2	O2A[1]	105.6(2)
C2A	N1A	Nd1	116.5(3)	O3W	Nd2	O4B[6]	70.5(2)
C10A	N1A	Nd1	124.9(3)	O4B[6]	Nd2	N1C[2]	68.35(18)
C2B	N1B	C10B	119.9(5)	O4B[6]	Nd2	O1B[1]	144.86(17)
C2C	N1C	C10C	117.0(5)	O4B[6]	Nd2	O2A[1]	82.7(2)

Symmetry codes: [1]1/2+x,3/2-y,+z; [2]-1/2+x,3/2-y,+z; [3]1-x,1-y,-1/2+z; [4]1-x,1-y,1/2+z; [5]+x,+y,-1+z; [6]+x,+y,1+z.

Table S8. Bond Angles for (4) $[Nd_2(Qdca)_3(H_2O)_4]\cdot 3H_2O$.

Atom	Atom	Atom	Angle/°	Atom	Atom	Atom	Angle/°
O1A[1]	Nd1	O2A[2]	73.53(16)	C2C	N1C	Nd2[5]	111.9(4)
O1A[1]	Nd1	O2B[3]	79.32(16)	C2C	N1C	C10C	117.9(5)
O1A[1]	Nd1	N1A[1]	61.64(16)	C4B	C5B	C10B	117.9(6)
O1A[1]	Nd1	O1W	140.7(2)	C4B	C5B	C6B	123.5(6)
O1A[1]	Nd1	O4W	156.3(2)	C6B	C5B	C10B	118.6(6)
O4A	Nd1	O1A[1]	91.86(17)	N1B	C10B	C5B	122.7(6)
O4A	Nd1	O2A[2]	149.90(18)	N1B	C10B	C9B	118.8(6)
O4A	Nd1	O2B[3]	76.04(17)	C9B	C10B	C5B	118.5(6)
O4A	Nd1	O4B	130.47(18)	C5B	C4B	C12B	122.7(6)
O4A	Nd1	N1A[1]	71.75(16)	C3B	C4B	C5B	118.4(6)
O4A	Nd1	O1W	107.2(2)	C3B	C4B	C12B	118.9(6)
O4A	Nd1	O4W	73.4(2)	N1A	C10A	C5A	122.3(6)
O2A[2]	Nd1	N1A[1]	119.94(16)	N1A	C10A	C9A	118.1(6)
O2A[2]	Nd1	O4W	127.8(2)	C9A	C10A	C5A	119.5(6)
O2B[3]	Nd1	O2A[2]	75.46(17)	C4B	C3B	C2B	120.0(6)
O2B[3]	Nd1	N1A[1]	127.37(16)	C5A	C4A	C12A	123.7(6)
O2B[3]	Nd1	O1W	72.8(2)	C3A	C4A	C5A	118.5(6)
O2B[3]	Nd1	O4W	113.7(2)	C3A	C4A	C12A	117.6(6)
O4B	Nd1	O1A[1]	100.93(17)	C10A	C5A	C4A	117.4(6)
O4B	Nd1	O2A[2]	78.92(18)	C6A	C5A	C10A	118.1(6)
O4B	Nd1	O2B[3]	153.19(18)	C6A	C5A	C4A	124.5(6)
O4B	Nd1	N1A[1]	73.07(16)	N1B	C2B	C3B	123.2(6)
O4B	Nd1	O1W	92.3(2)	N1B	C2B	C11B	116.6(5)
O4B	Nd1	O4W	76.6(2)	C3B	C2B	C11B	120.2(6)
O1W	Nd1	O2A[2]	73.1(2)	C10C	C5C	C6C	117.9(6)
O1W	Nd1	N1A[1]	156.7(2)	C4C	C5C	C6C	123.4(6)
O1W	Nd1	O4W	62.8(3)	C4C	C5C	C10C	118.7(6)
O4W	Nd1	N1A[1]	95.6(2)	C4A	C3A	C2A	120.3(6)
O3B	Nd2	O1B[4]	105.81(17)	O3B	C12B	C4B	116.7(6)
O3B	Nd2	O1C[5]	138.61(17)	O4B	C12B	O3B	125.5(6)
O3B	Nd2	O3W	146.89(19)	O4B	C12B	C4B	117.8(6)
O3B	Nd2	N1B[4]	73.48(16)	N1A	C2A	C3A	123.2(6)
O3B	Nd2	N1C[5]	87.50(16)	N1A	C2A	C11A	115.6(5)
O3B	Nd2	O2W	73.4(2)	C3A	C2A	C11A	121.1(6)
O1B[4]	Nd2	O3W	72.4(2)	C8B	C9B	C10B	119.6(6)
O1B[4]	Nd2	N1B[4]	62.48(16)	C7B	C6B	C5B	120.4(6)
O1B[4]	Nd2	N1C[5]	140.09(17)	C7C	C6C	C5C	121.3(7)
O1B[4]	Nd2	O2W	69.72(19)	O4A	C12A	C4A	118.0(6)
O1C[5]	Nd2	O1B[4]	82.60(16)	O3A	C12A	O4A	124.8(6)
O1C[5]	Nd2	O3W	74.50(19)	O3A	C12A	C4A	117.1(6)
O1C[5]	Nd2	N1B[4]	140.17(15)	O1A	C11A	C2A	115.4(6)

O1C[5]	Nd2	N1C[5]	64.23(16)	O2A	C11A	O1A	125.7(6)
O1C[5]	Nd2	O2W	72.0(2)	O2A	C11A	C2A	118.9(6)
O4C	Nd2	O3B	92.59(19)	O1B	C11B	C2B	117.0(6)
O4C	Nd2	O1B[4]	142.29(18)	O2B	C11B	O1B	124.3(6)
O4C	Nd2	O1C[5]	105.10(18)	O2B	C11B	C2B	118.6(6)
O4C	Nd2	O3W	74.3(2)	C8A	C9A	C10A	120.8(7)
O4C	Nd2	N1B[4]	93.12(16)	N1C	C10C	C5C	121.7(6)
O4C	Nd2	N1C[5]	71.68(17)	N1C	C10C	C9C	118.9(6)
O4C	Nd2	O2W	148.0(2)	C9C	C10C	C5C	119.4(6)
O3W	Nd2	N1B[4]	76.97(18)	N1C	C2C	C3C	123.0(6)
O3W	Nd2	N1C[5]	115.53(19)	N1C	C2C	C11C	117.0(6)
O3W	Nd2	O2W	131.8(2)	C3C	C2C	C11C	120.0(6)
N1B[4]	Nd2	N1C[5]	155.28(16)	C5C	C4C	C12C	123.5(6)
O2W	Nd2	N1B[4]	109.45(18)	C3C	C4C	C5C	118.2(6)
O2W	Nd2	N1C[5]	78.92(18)	C3C	C4C	C12C	118.3(6)
C11A	O1A	Nd1[1]	126.9(4)	C8C	C9C	C10C	121.2(7)
C12B	O3B	Nd2	173.7(4)	C4C	C3C	C2C	120.3(6)
C12A	O4A	Nd1	159.6(5)	C7A	C6A	C5A	120.6(7)
C11A	O2A	Nd1[6]	128.9(4)	C6A	C7A	C8A	120.7(7)
C11B	O1B	Nd2[4]	128.9(4)	O1C	C11C	C2C	117.3(6)
C11B	O2B	Nd1[3]	146.4(5)	O2C	C11C	O1C	124.7(7)
C12B	O4B	Nd1	161.6(4)	O2C	C11C	C2C	118.0(6)
C11C	O1C	Nd2[5]	124.4(4)	C9B	C8B	C7B	121.8(6)
C12C	O4C	Nd2	171.2(5)	C6B	C7B	C8B	121.0(7)
C10A	N1A	Nd1[1]	128.8(4)	O4C	C12C	C4C	117.3(6)
C2A	N1A	Nd1[1]	113.0(4)	O3C	C12C	O4C	125.5(7)
C2A	N1A	C10A	117.9(5)	O3C	C12C	C4C	117.2(6)
C10B	N1B	Nd2[4]	127.1(4)	C9A	C8A	C7A	120.3(7)
C2B	N1B	Nd2[4]	114.3(4)	C9C	C8C	C7C	120.1(7)
C2B	N1B	C10B	117.8(5)	C6C	C7C	C8C	120.0(7)
C10C	N1C	Nd2[5]	127.8(4)				

Symmetry codes:[1]2-x,2-y,1-z; [2]-1+x,+y,+z; [3]1-x,2-y,1-z; [4]1-x,1-y,1-z; [5]-x,1-y,-z; [6]1+x,+y,+z.

Table S9. Bond Angles for (8) $[Eu_2(Qdca)_3(H_2O)_4]\cdot H_2O$.

Atom	Atom	Atom	Angle/°	Atom	Atom	Atom	Angle/°
N1B[1]	Eu1	N1A	86.89(12)	C3A	C4A	C12A	116.1(5)
O1A	Eu1	N1A	62.30(13)	C5A	C4A	C12A	125.4(5)
O1A	Eu1	N1B[1]	76.98(15)	C3B	C4B	C5B	117.9(5)
O1A	Eu1	O1B[1]	83.12(14)	C3B	C4B	C12B	120.4(5)
O1A	Eu1	O3A[2]	146.39(14)	C5B	C4B	C12B	121.7(5)
O1A	Eu1	O4A[3]	99.33(15)	C3C	C4C	C5C	119.4(5)
O1A	Eu1	O4B	136.45(15)	C3C	C4C	C12C	117.7(5)
O1A	Eu1	O4W	77.08(16)	C5C	C4C	C12C	122.9(5)
O1B[1]	Eu1	N1A	138.37(13)	C4A	C5A	C10A	117.5(5)
O1B[1]	Eu1	N1B[1]	61.91(13)	C6A	C5A	C4A	124.2(5)
O1B[1]	Eu1	O4W	75.37(15)	C6A	C5A	C10A	118.2(5)
O3A[2]	Eu1	N1A	147.69(13)	C4B	C5B	C10B	118.2(5)
O3A[2]	Eu1	N1B[1]	110.47(14)	C6B	C5B	C4B	123.1(5)
O3A[2]	Eu1	O1B[1]	73.14(13)	C6B	C5B	C10B	118.8(5)
O3A[2]	Eu1	O4W	74.17(15)	C4C	C5C	C10C	117.3(5)
O4A[3]	Eu1	N1A	67.31(13)	C6C	C5C	C4C	124.6(5)
O4A[3]	Eu1	N1B[1]	151.81(13)	C6C	C5C	C10C	118.1(5)
O4A[3]	Eu1	O1B[1]	146.08(14)	C7A	C6A	C5A	121.2(5)
O4A[3]	Eu1	O3A[2]	88.24(14)	C7B	C6B	C5B	121.0(6)

O4A[3]	Eu1	O4W	72.32(15)	C7C	C6C	C5C	120.5(6)
O4B	Eu1	N1A	83.92(14)	C6A	C7A	C8A	119.6(6)
O4B	Eu1	N1B[1]	74.39(14)	C6B	C7B	C8B	119.8(6)
O4B	Eu1	O1B[1]	110.46(14)	C6C	C7C	C8C	121.8(6)
O4B	Eu1	O3A[2]	75.43(15)	C9A	C8A	C7A	120.7(6)
O4B	Eu1	O4A[3]	91.10(15)	C9B	C8B	C7B	121.5(6)
O4B	Eu1	O4W	145.60(15)	C9C	C8C	C7C	119.8(6)
O4W	Eu1	N1A	114.89(14)	C8A	C9A	C10A	120.7(5)
O4W	Eu1	N1B[1]	131.95(14)	C8B	C9B	C10B	120.6(6)
N1C	Eu2	C11C	47.03(14)	C8C	C9C	C10C	120.6(5)
O1C	Eu2	C11C	18.28(15)	C9A	C10A	C5A	119.5(5)
O1C	Eu2	N1C	65.08(15)	N1A	C10A	C5A	123.0(5)
O1C	Eu2	O1W	78.69(18)	N1A	C10A	C9A	117.5(5)
O1C	Eu2	O2B	146.63(16)	C9B	C10B	C5B	118.4(5)
O1C	Eu2	O2W	75.81(16)	N1B	C10B	C5B	122.8(5)
O1C	Eu2	O3C[2]	137.11(16)	N1B	C10B	C9B	118.8(5)
O1C	Eu2	O3W	73.15(17)	C9C	C10C	C5C	119.2(5)
O1C	Eu2	O4C[4]	103.43(16)	N1C	C10C	C5C	122.0(5)
O1W	Eu2	C11C	92.85(17)	N1C	C10C	C9C	118.8(5)
O1W	Eu2	N1C	132.38(16)	O1A	C11A	C2A	116.8(5)
O2B	Eu2	C11C	131.60(15)	O2A	C11A	C2A	119.8(5)
O2B	Eu2	N1C	91.68(14)	O2A	C11A	O1A	123.4(5)
O2B	Eu2	O1W	104.15(19)	O1B	C11B	C2B	117.6(5)
O2B	Eu2	O2W	74.45(15)	O2B	C11B	C2B	117.6(5)
O2B	Eu2	O3W	140.10(16)	O2B	C11B	O1B	124.8(5)
O2W	Eu2	C11C	70.05(15)	C2C	C11C	Eu2	83.7(3)
O2W	Eu2	N1C	72.71(15)	O1C	C11C	Eu2	34.0(3)
O2W	Eu2	O1W	69.10(19)	O1C	C11C	C2C	116.5(5)
O2W	Eu2	O3W	136.04(19)	O2C	C11C	Eu2	156.4(4)
O3C[2]	Eu2	C11C	155.10(17)	O2C	C11C	C2C	118.5(5)
O3C[2]	Eu2	N1C	154.36(15)	O2C	C11C	O1C	125.0(6)
O3C[2]	Eu2	O1W	72.30(17)	O3A	C12A	C4A	115.5(5)
O3C[2]	Eu2	O2B	72.56(16)	O3A	C12A	O4A	126.8(5)
O3C[2]	Eu2	O2W	119.91(16)	O4A	C12A	C4A	117.7(5)
O3C[2]	Eu2	O3W	69.25(18)	O3B	C12B	C4B	120.2(5)
O3W	Eu2	C11C	87.86(17)	O3B	C12B	O4B	125.1(5)
O3W	Eu2	N1C	118.93(17)	O4B	C12B	C4B	114.8(5)
O3W	Eu2	O1W	74.8(2)	O3C	C12C	C4C	115.8(5)
O4C[4]	Eu2	C11C	96.73(15)	O4C	C12C	C4C	118.7(5)
O4C[4]	Eu2	N1C	71.60(14)	O4C	C12C	O3C	125.4(5)
O4C[4]	Eu2	O1W	150.29(19)	C2A	N1A	Eu1	110.0(3)
O4C[4]	Eu2	O2B	90.25(16)	C2A	N1A	C10A	117.0(4)
O4C[4]	Eu2	O2W	140.53(17)	C10A	N1A	Eu1	132.0(3)
O4C[4]	Eu2	O3C[2]	87.99(15)	C2B	N1B	Eu1[1]	113.4(3)
O4C[4]	Eu2	O3W	77.5(2)	C2B	N1B	C10B	117.1(5)
C3A	C2A	C11A	119.5(5)	C10B	N1B	Eu1[1]	129.0(3)
N1A	C2A	C3A	122.9(5)	C2C	N1C	Eu2	111.1(4)
N1A	C2A	C11A	117.5(5)	C2C	N1C	C10C	118.1(5)
C3B	C2B	C11B	120.3(5)	C10C	N1C	Eu2	130.1(3)
N1B	C2B	C3B	123.7(5)	C11A	O1A	Eu1	132.4(4)
N1B	C2B	C11B	116.0(5)	C11B	O1B	Eu1[1]	130.3(3)
C3C	C2C	C11C	119.9(5)	C11C	O1C	Eu2	127.7(4)
N1C	C2C	C3C	122.7(5)	C11B	O2B	Eu2	128.0(4)
N1C	C2C	C11C	117.4(5)	C12A	O3A	Eu1[5]	138.8(4)

C4A	C3A	C2A	121.1(5)	C12C	O3C	Eu2^{5}	138.7(4)
C4B	C3B	C2B	120.1(5)	C12A	O4A	Eu1^{3}	157.1(4)
C4C	C3C	C2C	120.2(5)	C12B	O4B	Eu1	174.7(4)
C3A	C4A	C5A	118.4(5)	C12C	O4C	Eu2^{4}	166.7(5)

Symmetry codes:^{1}2-x,1-y,1-z; ^{2}1+x,+y,+z; ^{3}1-x,-y,1-z; ^{4}1-x,1-y,2-z; ^{5}-1+x,+y,+z.

Table S10. Bond Angles for (9) $[Tb_2(Qdca)_3(H_2O)_4]\cdot H_2O$.

Atom	Atom	Atom	Angle/°	Atom	Atom	Atom	Angle/°
C3A	C2A	C11A	119.5(5)	C2B	N1B	C10B	116.1(5)
N1A	C2A	C3A	122.7(5)	C2B	N1B	Tb1^{1}	114.0(4)
N1A	C2A	C11A	117.8(5)	C10B	N1B	Tb1^{1}	129.4(3)
C3B	C2B	C11B	120.0(5)	C2C	N1C	C10C	117.9(5)
N1B	C2B	C3B	124.7(6)	C2C	N1C	Tb2	111.2(4)
N1B	C2B	C11B	115.3(5)	C10C	N1C	Tb2	130.1(4)
C3C	C2C	C11C	118.4(5)	C11A	O1A	Tb1	132.7(4)
N1C	C2C	C3C	123.7(5)	C11B	O1B	Tb1^{1}	130.6(4)
N1C	C2C	C11C	117.9(5)	C11C	O1C	Tb2	127.4(4)
C4A	C3A	C2A	121.1(5)	C11B	O2B	Tb2	129.2(4)
C4B	C3B	C2B	120.0(5)	C12A	O3A	Tb1^{2}	139.3(4)
C4C	C3C	C2C	119.3(6)	C12C	O3C	Tb2^{2}	139.1(4)
C3A	C4A	C5A	118.8(5)	C12A	O4A	Tb1^{3}	157.4(4)
C3A	C4A	C12A	115.9(5)	C12B	O4B	Tb1	173.7(4)
C5A	C4A	C12A	125.1(5)	C12C	O4C	Tb2^{4}	168.1(5)
C3B	C4B	C5B	118.4(5)	N1B^{1}	Tb1	N1A	86.80(13)
C3B	C4B	C12B	121.1(5)	O1A	Tb1	N1A	62.96(14)
C5B	C4B	C12B	120.5(5)	O1A	Tb1	N1B^{1}	76.83(15)
C3C	C4C	C5C	119.4(5)	O1A	Tb1	O1B^{1}	82.97(14)
C3C	C4C	C12C	116.8(5)	O1A	Tb1	O3A^{5}	146.44(15)
C5C	C4C	C12C	123.7(5)	O1A	Tb1	O4A^{3}	99.13(15)
C6A	C5A	C4A	123.8(5)	O1A	Tb1	O4W	76.85(16)
C6A	C5A	C10A	119.8(5)	O1B^{1}	Tb1	N1A	138.74(13)
C10A	C5A	C4A	116.4(5)	O1B^{1}	Tb1	N1B^{1}	62.00(14)
C6B	C5B	C4B	123.7(5)	O1B^{1}	Tb1	O4W	74.84(15)
C6B	C5B	C10B	118.8(5)	O3A^{5}	Tb1	N1A	146.96(14)
C10B	C5B	C4B	117.5(5)	O3A^{5}	Tb1	N1B^{1}	110.83(14)
C6C	C5C	C4C	124.0(5)	O3A^{5}	Tb1	O1B^{1}	73.43(14)
C6C	C5C	C10C	118.7(6)	O3A^{5}	Tb1	O4W	74.19(16)
C10C	C5C	C4C	117.2(5)	O4A^{3}	Tb1	N1A	67.29(14)
C7A	C6A	C5A	119.9(6)	O4A^{3}	Tb1	N1B^{1}	152.00(15)
C7B	C6B	C5B	120.9(6)	O4A^{3}	Tb1	O1B^{1}	145.74(15)
C7C	C6C	C5C	120.6(6)	O4A^{3}	Tb1	O3A^{5}	88.03(15)
C6A	C7A	C8A	120.6(6)	O4A^{3}	Tb1	O4W	72.45(16)
C6B	C7B	C8B	120.7(6)	O4B	Tb1	N1A	83.02(14)
C6C	C7C	C8C	120.3(6)	O4B	Tb1	N1B^{1}	74.23(14)
C9A	C8A	C7A	120.6(6)	O4B	Tb1	O1A	136.09(16)
C9B	C8B	C7B	120.8(6)	O4B	Tb1	O1B^{1}	110.65(14)
C9C	C8C	C7C	120.8(6)	O4B	Tb1	O3A^{5}	75.79(16)
C8A	C9A	C10A	120.6(5)	O4B	Tb1	O4A^{3}	91.57(15)
C8B	C9B	C10B	120.7(6)	O4B	Tb1	O4W	146.27(16)
C8C	C9C	C10C	120.5(6)	O4W	Tb1	N1A	115.53(15)
C5A	C10A	C9A	118.4(5)	O4W	Tb1	N1B^{1}	131.43(15)
N1A	C10A	C5A	124.1(5)	O1C	Tb2	N1C	65.67(15)
N1A	C10A	C9A	117.5(5)	O1C	Tb2	O1W	75.39(17)
C9B	C10B	C5B	118.0(5)	O1C	Tb2	O2B	146.69(17)

N1B	C10B	C5B	123.2(5)	O1C	Tb2	O2W	72.69(18)
N1B	C10B	C9B	118.8(5)	O1C	Tb2	O3C^{5}	136.52(16)
C9C	C10C	C5C	119.0(5)	O1C	Tb2	O3W	77.79(18)
N1C	C10C	C5C	122.2(5)	O1C	Tb2	O4C^{4}	104.26(17)
N1C	C10C	C9C	118.8(5)	O1W	Tb2	N1C	72.74(15)
O1A	C11A	C2A	116.3(5)	O1W	Tb2	O2W	136.1(2)
O2A	C11A	C2A	119.3(5)	O1W	Tb2	O3W	69.73(19)
O2A	C11A	O1A	124.4(6)	O2B	Tb2	N1C	91.71(15)
O1B	C11B	C2B	117.3(5)	O2B	Tb2	O1W	74.59(16)
O1B	C11B	O2B	124.3(5)	O2B	Tb2	O2W	140.49(18)
O2B	C11B	C2B	118.4(5)	O2B	Tb2	O3W	104.70(19)
O1C	C11C	C2C	115.2(5)	O2W	Tb2	N1C	118.24(18)
O2C	C11C	C2C	119.3(5)	O2W	Tb2	O3W	74.6(2)
O2C	C11C	O1C	125.5(6)	O3C^{5}	Tb2	N1C	154.18(15)
O3A	C12A	C4A	115.2(5)	O3C^{5}	Tb2	O1W	120.46(16)
O3A	C12A	O4A	126.8(5)	O3C^{5}	Tb2	O2B	72.83(17)
O4A	C12A	C4A	118.0(5)	O3C^{5}	Tb2	O2W	69.43(18)
O3B	C12B	C4B	118.9(5)	O3C^{5}	Tb2	O3W	72.31(17)
O3B	C12B	O4B	126.5(5)	O3W	Tb2	N1C	132.75(16)
O4B	C12B	C4B	114.6(5)	O4C^{4}	Tb2	N1C	71.66(15)
O3C	C12C	C4C	116.5(5)	O4C^{4}	Tb2	O1W	140.60(17)
O3C	C12C	O4C	125.3(6)	O4C^{4}	Tb2	O2B	90.10(16)
O4C	C12C	C4C	118.1(6)	O4C^{4}	Tb2	O2W	77.1(2)
C2A	N1A	C10A	116.9(5)	O4C^{4}	Tb2	O3C^{5}	87.46(15)
C2A	N1A	Tb1	109.4(4)	O4C^{4}	Tb2	O3W	149.57(19)
C10A	N1A	Tb1	132.7(4)				

Symmetry codes:^{1}2-x,1-y,1-z; ^{2}-1+x,+y,+z; ^{3}1-x,-y,1-z; ^{4}1-x,1-y,2-z; ^{5}1+x,+y,+z.

Table S11. Bond Angles for (13) [Er$_2$(Qdca)$_3$(H$_2$O)$_4$]·4H$_2$O.

Atom	Atom	Atom	Angle/°	Atom	Atom	Atom	Angle/°
O1A	Er1	N1B^{1}	79.49(10)	C3C	C4C	C5C	118.7(3)
O1A	Er1	O1B^{1}	84.74(10)	C3C	C4C	C12C	119.4(3)
O1A	Er1	O1W	78.90(11)	C5C	C4C	C12C	121.9(3)
O1A	Er1	O3A^{2}	149.85(11)	C4A	C5A	C6A	124.3(4)
O1A	Er1	O4A^{3}	97.06(10)	C4A	C5A	C10A	117.4(3)
O1A	Er1	O4B	132.48(11)	C6A	C5A	C10A	118.3(4)
O1B^{1}	Er1	N1B^{1}	62.89(9)	C4B	C5B	C6B	123.8(4)
O1B^{1}	Er1	O1W	75.20(10)	C10B	C5B	C4B	118.0(3)
O1W	Er1	N1B^{1}	134.12(10)	C10B	C5B	C6B	118.2(3)
O3A^{2}	Er1	N1B^{1}	110.04(10)	C6C	C5C	C4C	122.4(3)
O3A^{2}	Er1	O1B^{1}	75.45(10)	C10C	C5C	C4C	118.2(3)
O3A^{2}	Er1	O1W	74.24(11)	C10C	C5C	C6C	119.4(4)
O4A^{3}	Er1	N1B^{1}	149.43(10)	C7A	C6A	C5A	120.7(4)
O4A^{3}	Er1	O1B^{1}	147.54(10)	C7B	C6B	C5B	121.0(4)
O4A^{3}	Er1	O1W	73.39(11)	C7C	C6C	C5C	120.8(4)
O4A^{3}	Er1	O3A^{2}	88.26(10)	C6A	C7A	C8A	120.8(4)
O4B	Er1	N1B^{1}	71.41(10)	C6B	C7B	C8B	120.9(4)
O4B	Er1	O1B^{1}	112.43(10)	C6C	C7C	C8C	119.7(4)
O4B	Er1	O1W	146.99(11)	C9A	C8A	C7A	120.3(4)
O4B	Er1	O3A^{2}	76.87(11)	C9B	C8B	C7B	120.0(4)
O4B	Er1	O4A^{3}	90.21(10)	C9C	C8C	C7C	120.7(4)
O1C	Er2	N1C	65.66(10)	C8A	C9A	C10A	120.5(4)
O1C	Er2	O2B	153.87(11)	C8B	C9B	C10B	121.1(4)
O1C	Er2	O2W	70.43(11)	C8C	C9C	C10C	120.7(4)

O1C	Er2	O3C^{4}	110.82(11)	C9A	C10A	C5A	119.4(4)
O1C	Er2	O3W	79.41(13)	N1A	C10A	C5A	122.7(3)
O1C	Er2	O4C^{2}	133.94(10)	N1A	C10A	C9A	117.9(4)
O1C	Er2	O4W	75.63(11)	C9B	C10B	C5B	118.8(3)
O2B	Er2	N1C	95.63(10)	N1B	C10B	C5B	122.6(3)
O2B	Er2	O2W	135.14(10)	N1B	C10B	C9B	118.6(3)
O2B	Er2	O4C^{2}	69.34(10)	C5C	C10C	C9C	118.6(4)
O2W	Er2	N1C	108.69(10)	N1C	C10C	C5C	122.2(3)
O3C^{4}	Er2	N1C	73.62(9)	N1C	C10C	C9C	119.2(3)
O3C^{4}	Er2	O2B	78.58(10)	O1A	C11A	C2A	116.4(3)
O3C^{4}	Er2	O2W	73.27(11)	O2A	C11A	C2A	119.4(3)
O3C^{4}	Er2	O3W	134.52(12)	O2A	C11A	O1A	124.1(4)
O3C^{4}	Er2	O4C^{2}	85.54(10)	O1B	C11B	C2B	117.3(3)
O3W	Er2	N1C	71.18(11)	O1B	C11B	O2B	124.6(3)
O3W	Er2	O2B	77.19(12)	O2B	C11B	C2B	118.2(3)
O3W	Er2	O2W	145.84(13)	O1C	C11C	C2C	116.4(3)
O3W	Er2	O4C^{2}	119.96(11)	O2C	C11C	C2C	119.5(3)
O4C^{2}	Er2	N1C	156.59(10)	O2C	C11C	O1C	124.1(4)
O4C^{2}	Er2	O2W	74.24(10)	O3A	C12A	C4A	115.5(3)
O4W	Er2	N1C	132.25(11)	O4A	C12A	C4A	118.6(3)
O4W	Er2	O2B	109.05(11)	O4A	C12A	O3A	125.8(3)
O4W	Er2	O2W	81.88(13)	O3B	C12B	C4B	118.7(3)
O4W	Er2	O3C^{4}	149.66(12)	O3B	C12B	O4B	124.8(4)
O4W	Er2	O3W	75.30(14)	O4B	C12B	C4B	116.5(3)
O4W	Er2	O4C^{2}	70.92(11)	O3C	C12C	C4C	116.1(3)
C3A	C2A	C11A	119.7(3)	O4C	C12C	C4C	118.1(3)
N1A	C2A	C3A	123.6(4)	O4C	C12C	O3C	125.8(3)
N1A	C2A	C11A	116.7(3)	C2A	N1A	C10A	117.4(3)
C3B	C2B	C11B	120.9(3)	C2B	N1B	Er1^{1}	113.1(2)
N1B	C2B	C3B	124.0(3)	C2B	N1B	C10B	117.5(3)
N1B	C2B	C11B	115.0(3)	C10B	N1B	Er1^{1}	127.8(2)
C3C	C2C	C11C	119.7(3)	C2C	N1C	Er2	113.4(2)
N1C	C2C	C3C	123.9(4)	C2C	N1C	C10C	117.4(3)
N1C	C2C	C11C	116.4(3)	C10C	N1C	Er2	129.1(2)
C4A	C3A	C2A	120.2(4)	C11A	O1A	Er1	137.6(3)
C4B	C3B	C2B	119.2(3)	C11B	O1B	Er1^{1}	129.9(2)
C4C	C3C	C2C	119.6(4)	C11C	O1C	Er2	127.7(3)
C3A	C4A	C5A	118.6(3)	C11B	O2B	Er2	122.9(2)
C3A	C4A	C12A	116.9(3)	C12A	O3A	Er1^{5}	145.4(2)
C5A	C4A	C12A	124.4(3)	C12C	O3C	Er2^{4}	139.6(3)
C3B	C4B	C5B	118.5(3)	C12A	O4A	Er1^{3}	168.6(3)
C3B	C4B	C12B	121.3(3)	C12B	O4B	Er1	161.5(3)
C5B	C4B	C12B	120.2(3)	C12C	O4C	Er2^{5}	137.3(2)

Symmetry codes: ^{1}-x,1-y,1-z; ^{2}-1+x,+y,+z; ^{3}1-x,-y,1-z; ^{4}1-x,1-y,2-z; ^{5}1+x,+y,+z.

Table S12. Torsion Angles for (1) $[Nd_2(Qdca)_3(H_2O)_3]$.

A	B	C	D	Angle/°	A	B	C	D	Angle/°
C2A	C3A	C4A	C5A	-1.6(14)	C5C	C10C	N1C	Nd2^{2}	-177.7(5)
C2A	C3A	C4A	C12A	178.6(9)	C6A	C5A	C10A	C9A	3.7(13)
C2A	C11A	O1A	Nd1	25.4(9)	C6A	C5A	C10A	N1A	-177.5(8)
C2A	C11A	O2A	Nd2^{1}	-172.3(6)	C6A	C7A	C8A	C9A	2.4(18)
C2B	C3B	C4B	C5B	1.5(9)	C6B	C5B	C10B	C9B	1.6(11)
C2B	C3B	C4B	C12B	177.3(6)	C6B	C5B	C10B	N1B	-175.0(7)
C2B	C11B	O1B	Nd2^{1}	-92.7(13)	C6B	C7B	C8B	C9B	1.2(11)
C2B	C11B	O2B	Nd1	158.2(5)	C6C	C5C	C10C	C9C	-0.4(10)

C2C	C3C	C4C	C5C	0.4(11)	C6C	C5C	C10C	N1C	179.1(7)
C2C	C3C	C4C	C12C	176.9(7)	C6C	C7C	C8C	C9C	-2.4(12)
C2C	C11C	O1C	Nd2[2]	-8.4(9)	C7A	C8A	C9A	C10A	-2.3(17)
C3A	C2A	C11A	O1A	166.8(8)	C7B	C8B	C9B	C10B	0.6(12)
C3A	C2A	C11A	O2A	-12.1(13)	C7C	C8C	C9C	C10C	1.7(12)
C3A	C2A	N1A	C10A	-3.5(13)	C8A	C9A	C10A	C5A	-0.8(14)
C3A	C2A	N1A	Nd1	177.3(7)	C8A	C9A	C10A	N1A	-179.6(9)
C3A	C4A	C5A	C6A	177.8(9)	C8B	C9B	C10B	C5B	-2.0(12)
C3A	C4A	C5A	C10A	-0.8(13)	C8B	C9B	C10B	N1B	174.8(7)
C3A	C4A	C12A	O3A	88.4(10)	C8C	C9C	C10C	C5C	-0.3(11)
C3A	C4A	C12A	O4A	-93.1(10)	C8C	C9C	C10C	N1C	-179.9(8)
C3B	C2B	C11B	O1B	174.3(6)	C9A	C10A	N1A	C2A	179.7(9)
C3B	C2B	C11B	O2B	-5.4(9)	C9A	C10A	N1A	Nd1	-1.2(11)
C3B	C2B	N1B	C10B	-1.6(9)	C9B	C10B	N1B	C2B	-176.8(6)
C3B	C4B	C5B	C6B	174.2(7)	C9C	C10C	N1C	C2C	179.1(7)
C3B	C4B	C5B	C10B	-3.0(10)	C9C	C10C	N1C	Nd2[2]	1.9(10)
C3B	C4B	C12B	O3B	-61.5(8)	C10A	C5A	C6A	C7A	-3.8(14)
C3B	C4B	C12B	O4B	120.1(7)	C10B	C5B	C6B	C7B	0.2(11)
C3C	C2C	C11C	O1C	-173.5(6)	C10C	C5C	C6C	C7C	-0.2(11)
C3C	C2C	C11C	O2C	7.0(10)	C11A	C2A	C3A	C4A	-174.4(8)
C3C	C2C	N1C	C10C	-0.4(11)	C11A	C2A	N1A	C10A	175.0(8)
C3C	C2C	N1C	Nd2[2]	177.4(6)	C11A	C2A	N1A	Nd1	-4.3(10)
C3C	C4C	C5C	C6C	-178.9(7)	C11B	C2B	C3B	C4B	-174.8(6)
C3C	C4C	C5C	C10C	-1.2(10)	C11B	C2B	N1B	C10B	174.4(6)
C3C	C4C	C12C	O3C	-152.6(8)	C11C	C2C	C3C	C4C	-179.3(7)
C3C	C4C	C12C	O4C	24.4(10)	C11C	C2C	N1C	C10C	179.3(6)
C4A	C5A	C6A	C7A	177.6(10)	C11C	C2C	N1C	Nd2[2]	-2.8(8)
C4A	C5A	C10A	C9A	-177.6(9)	C12A	C4A	C5A	C6A	-2.4(13)
C4A	C5A	C10A	N1A	1.2(13)	C12A	C4A	C5A	C10A	179.0(9)
C4A	C12A	O3A	Nd1[3]	63.9(12)	C12B	C4B	C5B	C6B	-1.6(12)
C4A	C12A	O4A	Nd1[4]	-51.0(16)	C12B	C4B	C5B	C10B	-178.9(7)
C4B	C5B	C6B	C7B	-177.0(7)	C12C	C4C	C5C	C6C	4.8(11)
C4B	C5B	C10B	C9B	179.0(7)	C12C	C4C	C5C	C10C	-177.5(6)
C4B	C5B	C10B	N1B	2.4(10)	N1A	C2A	C3A	C4A	3.9(15)
C4B	C12B	O3B	Nd2	10.7(13)	N1A	C2A	C11A	O1A	-11.7(11)
C4B	C12B	O4B	Nd2[5]	-80.4(9)	N1A	C2A	C11A	O2A	169.4(8)
C4C	C5C	C6C	C7C	177.5(7)	N1B	C2B	C3B	C4B	0.9(9)
C4C	C5C	C10C	C9C	-178.3(7)	N1B	C2B	C11B	O1B	-1.7(9)
C4C	C5C	C10C	N1C	1.3(10)	N1B	C2B	C11B	O2B	178.7(6)
C4C	C12C	O3C	Nd1	177.8(6)	N1C	C2C	C3C	C4C	0.5(12)
C4C	C12C	O4C	Nd1	-177.8(6)	N1C	C2C	C11C	O1C	6.7(9)
C5A	C4A	C12A	O3A	-91.4(9)	N1C	C2C	C11C	O2C	-172.8(6)
C5A	C4A	C12A	O4A	87.1(9)	O1A	C11A	O2A	Nd2[1]	9.0(10)
C5A	C6A	C7A	C8A	0.8(16)	O1B	C11B	O2B	Nd1	-21.4(12)
C5A	C10A	N1A	C2A	0.9(13)	O2A	C11A	O1A	Nd1	-155.8(6)
C5A	C10A	N1A	Nd1	-180.0(6)	O2B	C11B	O1B	Nd2[1]	86.9(13)
C5B	C4B	C12B	O3B	114.3(7)	O2C	C11C	O1C	Nd2[2]	171.1(5)
C5B	C4B	C12B	O4B	-64.1(8)	O3A	C12A	O4A	Nd1[4]	127.3(13)
C5B	C6B	C7B	C8B	-1.6(11)	O3B	C12B	O4B	Nd2[5]	101.4(9)
C5B	C10B	N1B	C2B	-0.1(10)	O3C	C12C	O4C	Nd1	-1.0(9)
C5C	C4C	C12C	O3C	23.8(12)	O4A	C12A	O3A	Nd1[3]	-114.5(11)
C5C	C4C	C12C	O4C	-159.3(7)	O4B	C12B	O3B	Nd2	-171.1(8)
C5C	C6C	C7C	C8C	1.6(12)	O4C	C12C	O3C	Nd1	1.0(9)
C5C	C10C	N1C	C2C	-0.4(10)					

Symmetry codes: [1]-1/2+x,3/2-y,+z; [2]1/2+x,3/2-y,+z; [3]1-x,1-y,-1/2+z; [4]1-x,1-y,1/2+z; [5]+x,+y,-1+z.

Table S13. Torsion Angles for (4) $[Nd_2(Qdca)_3(H_2O)_4]·3H_2O$.

A	B	C	D	Angle/°	A	B	C	D	Angle/°
Nd1[1]	O1A	C11A	O2A	145.0(5)	C3B	C2B	C11B	O1B	171.2(6)
Nd1[1]	O1A	C11A	C2A	-35.2(8)	C3B	C2B	C11B	O2B	-7.0(10)
Nd1	O4A	C12A	O3A	83.0(15)	C4A	C5A	C6A	C7A	178.7(7)
Nd1	O4A	C12A	C4A	-91.4(14)	C4A	C3A	C2A	N1A	-3.9(10)
Nd1[2]	O2A	C11A	O1A	-34.7(10)	C4A	C3A	C2A	C11A	172.3(6)
Nd1[2]	O2A	C11A	C2A	145.5(5)	C5A	C10A	C9A	C8A	-0.3(11)
Nd1[3]	O2B	C11B	O1B	92.3(10)	C5A	C4A	C3A	C2A	6.5(10)
Nd1[3]	O2B	C11B	C2B	-89.7(9)	C5A	C4A	C12A	O4A	-32.9(10)
Nd1	O4B	C12B	O3B	-153.0(12)	C5A	C4A	C12A	O3A	152.4(7)
Nd1	O4B	C12B	C4B	28(2)	C5A	C6A	C7A	C8A	0.4(12)
Nd1[1]	N1A	C10A	C5A	-169.0(5)	C2B	N1B	C10B	C5B	-3.0(9)
Nd1[1]	N1A	C10A	C9A	8.1(9)	C2B	N1B	C10B	C9B	177.1(6)
Nd1[1]	N1A	C2A	C3A	172.7(5)	C5C	C6C	C7C	C8C	-0.7(13)
Nd1[1]	N1A	C2A	C11A	-3.7(7)	C5C	C10C	C9C	C8C	0.2(11)
Nd2[4]	O1B	C11B	O2B	-179.3(5)	C5C	C4C	C3C	C2C	-4.8(10)
Nd2[4]	O1B	C11B	C2B	2.7(9)	C5C	C4C	C12C	O4C	59.5(10)
Nd2[5]	O1C	C11C	O2C	-155.3(6)	C5C	C4C	C12C	O3C	-120.3(9)
Nd2[5]	O1C	C11C	C2C	24.5(9)	C3A	C4A	C5A	C10A	-3.8(9)
Nd2[4]	N1B	C10B	C5B	166.2(5)	C3A	C4A	C5A	C6A	176.5(7)
Nd2[4]	N1B	C10B	C9B	-13.7(8)	C3A	C4A	C12A	O4A	142.7(7)
Nd2[4]	N1B	C2B	C3B	-170.3(5)	C3A	C4A	C12A	O3A	-32.0(10)
Nd2[4]	N1B	C2B	C11B	9.7(7)	C3A	C2A	C11A	O1A	-154.2(6)
Nd2[5]	N1C	C10C	C5C	156.1(5)	C3A	C2A	C11A	O2A	25.6(9)
Nd2[5]	N1C	C10C	C9C	-24.0(9)	C12B	C4B	C3B	C2B	177.3(6)
Nd2[5]	N1C	C2C	C3C	-162.4(5)	C2A	N1A	C10A	C5A	4.3(9)
Nd2[5]	N1C	C2C	C11C	15.5(7)	C2A	N1A	C10A	C9A	-178.5(6)
N1A	C10A	C5A	C4A	-1.7(10)	C9B	C8B	C7B	C6B	0.0(12)
N1A	C10A	C5A	C6A	178.1(6)	C6B	C5B	C10B	N1B	-178.5(6)
N1A	C10A	C9A	C8A	-177.5(7)	C6B	C5B	C10B	C9B	1.5(9)
N1A	C2A	C11A	O1A	22.3(8)	C6B	C5B	C4B	C3B	-179.1(6)
N1A	C2A	C11A	O2A	-157.9(6)	C6B	C5B	C4B	C12B	2.2(10)
N1B	C10B	C9B	C8B	178.7(7)	C6C	C5C	C10C	N1C	-178.7(6)
N1B	C2B	C11B	O1B	-8.8(9)	C6C	C5C	C10C	C9C	1.4(10)
N1B	C2B	C11B	O2B	173.0(6)	C6C	C5C	C4C	C3C	-176.2(7)
N1C	C10C	C9C	C8C	-179.7(7)	C6C	C5C	C4C	C12C	0.7(11)
N1C	C2C	C3C	C4C	3.4(11)	C12A	C4A	C5A	C10A	171.8(6)
N1C	C2C	C11C	O1C	-26.6(9)	C12A	C4A	C5A	C6A	-7.9(11)
N1C	C2C	C11C	O2C	153.3(7)	C12A	C4A	C3A	C2A	-169.4(6)
C5B	C10B	C9B	C8B	-1.2(10)	C9A	C10A	C5A	C4A	-178.8(6)
C5B	C4B	C3B	C2B	-1.5(10)	C9A	C10A	C5A	C6A	1.0(10)
C5B	C4B	C12B	O3B	60.6(9)	C10C	N1C	C2C	C3C	1.6(10)
C5B	C4B	C12B	O4B	-120.0(7)	C10C	N1C	C2C	C11C	179.5(6)
C5B	C6B	C7B	C8B	0.3(12)	C10C	C5C	C6C	C7C	-1.1(11)
C10B	N1B	C2B	C3B	0.4(9)	C10C	C5C	C4C	C3C	1.5(10)
C10B	N1B	C2B	C11B	-179.6(6)	C10C	C5C	C4C	C12C	178.3(7)
C10B	C5B	C4B	C3B	-1.0(9)	C10C	C9C	C8C	C7C	-2.0(13)
C10B	C5B	C4B	C12B	-179.8(6)	C2C	N1C	C10C	C5C	-5.0(10)
C10B	C5B	C6B	C7B	-1.0(10)	C2C	N1C	C10C	C9C	174.9(7)
C10B	C9B	C8B	C7B	0.5(12)	C4C	C5C	C6C	C7C	176.5(7)
C4B	C5B	C10B	N1B	3.4(9)	C4C	C5C	C10C	N1C	3.5(10)
C4B	C5B	C10B	C9B	-176.7(6)	C4C	C5C	C10C	C9C	-176.4(7)
C4B	C5B	C6B	C7B	177.0(7)	C9C	C8C	C7C	C6C	2.2(13)

C4B	C3B	C2B	N1B	1.9(10)	C3C	C2C	C11C	O1C	151.4(7)
C4B	C3B	C2B	C11B	-178.1(6)	C3C	C2C	C11C	O2C	-28.7(10)
C10A	N1A	C2A	C3A	-1.6(10)	C3C	C4C	C12C	O4C	-123.6(8)
C10A	N1A	C2A	C11A	-178.0(5)	C3C	C4C	C12C	O3C	56.6(11)
C10A	C5A	C6A	C7A	-1.1(11)	C6A	C7A	C8A	C9A	0.3(12)
C10A	C9A	C8A	C7A	-0.4(12)	C11C	C2C	C3C	C4C	-174.4(7)
C3B	C4B	C12B	O3B	-118.2(7)	C12C	C4C	C3C	C2C	178.1(6)
C3B	C4B	C12B	O4B	61.2(9)					

Symmetry codes: [1]2-x,2-y,1-z; [2]1+x,+y,+z; [3]1-x,1-y,1-z; [4]1-x,2-y,1-z; [5]-x,1-y,-z.

Table S14. Torsion Angles for (8) $[Eu_2(Qdca)_3(H_2O)_4]\cdot H_2O$.

A	B	C	D	Angle/°	A	B	C	D	Angle/°
C2A	C3A	C4A	C5A	1.9(8)	C5C	C10C	N1C	C2C	-4.0(8)
C2A	C3A	C4A	C12A	-173.5(5)	C6A	C5A	C10A	C9A	2.6(8)
C2A	C11A	O1A	Eu1	4.5(8)	C6A	C5A	C10A	N1A	-178.7(5)
C2B	C3B	C4B	C5B	3.3(8)	C6A	C7A	C8A	C9A	1.3(10)
C2B	C3B	C4B	C12B	-175.5(5)	C6B	C5B	C10B	C9B	-0.5(8)
C2B	C11B	O1B	Eu1[1]	-1.8(8)	C6B	C5B	C10B	N1B	-180.0(5)
C2B	C11B	O2B	Eu2	154.6(4)	C6B	C7B	C8B	C9B	1.1(11)
C2C	C3C	C4C	C5C	-5.6(8)	C6C	C5C	C10C	C9C	-0.3(8)
C2C	C3C	C4C	C12C	171.2(5)	C6C	C5C	C10C	N1C	-179.1(5)
C2C	C11C	O1C	Eu2	16.7(8)	C6C	C7C	C8C	C9C	-1.5(11)
C3A	C2A	C11A	O1A	-172.0(5)	C7A	C8A	C9A	C10A	-0.5(10)
C3A	C2A	C11A	O2A	5.5(9)	C7B	C8B	C9B	C10B	0.5(10)
C3A	C2A	N1A	Eu1	167.7(4)	C7C	C8C	C9C	C10C	2.6(10)
C3A	C2A	N1A	C10A	-2.4(8)	C8A	C9A	C10A	C5A	-1.5(9)
C3A	C4A	C5A	C6A	177.3(5)	C8A	C9A	C10A	N1A	179.7(6)
C3A	C4A	C5A	C10A	-2.9(8)	C8B	C9B	C10B	C5B	-0.7(9)
C3A	C4A	C12A	O3A	-32.1(7)	C8B	C9B	C10B	N1B	178.8(6)
C3A	C4A	C12A	O4A	145.6(5)	C8C	C9C	C10C	C5C	-1.7(9)
C3B	C2B	C11B	O1B	-171.0(5)	C8C	C9C	C10C	N1C	177.0(6)
C3B	C2B	C11B	O2B	10.2(8)	C9A	C10A	N1A	Eu1	12.4(7)
C3B	C2B	N1B	Eu1[1]	169.6(4)	C9A	C10A	N1A	C2A	179.9(5)
C3B	C2B	N1B	C10B	-3.0(8)	C9B	C10B	N1B	Eu1[1]	12.3(8)
C3B	C4B	C5B	C6B	176.8(6)	C9B	C10B	N1B	C2B	-176.5(5)
C3B	C4B	C5B	C10B	-3.2(8)	C9C	C10C	N1C	Eu2	-13.3(8)
C3B	C4B	C12B	O3B	-58.7(8)	C9C	C10C	N1C	C2C	177.2(5)
C3B	C4B	C12B	O4B	120.7(6)	C10A	C5A	C6A	C7A	-1.7(9)
C3C	C2C	C11C	Eu2	171.4(5)	C10B	C5B	C6B	C7B	2.0(9)
C3C	C2C	C11C	O1C	162.1(5)	C10C	C5C	C6C	C7C	1.5(9)
C3C	C2C	C11C	O2C	-17.2(8)	C11A	C2A	C3A	C4A	178.5(5)
C3C	C2C	N1C	Eu2	-169.1(4)	C11A	C2A	N1A	Eu1	-9.9(6)
C3C	C2C	N1C	C10C	2.3(8)	C11A	C2A	N1A	C10A	179.9(5)
C3C	C4C	C5C	C6C	-176.1(6)	C11B	C2B	C3B	C4B	179.6(5)
C3C	C4C	C5C	C10C	3.8(8)	C11B	C2B	N1B	Eu1[1]	-10.3(6)
C3C	C4C	C12C	O3C	37.7(8)	C11B	C2B	N1B	C10B	177.2(5)
C3C	C4C	C12C	O4C	-139.2(6)	C11C	C2C	C3C	C4C	-176.9(5)
C4A	C5A	C6A	C7A	178.1(6)	C11C	C2C	N1C	Eu2	10.4(6)
C4A	C5A	C10A	C9A	-177.3(5)	C11C	C2C	N1C	C10C	-178.2(5)
C4A	C5A	C10A	N1A	1.4(8)	C12A	C4A	C5A	C6A	-7.8(9)
C4A	C12A	O3A	Eu1[2]	-151.0(4)	C12A	C4A	C5A	C10A	172.0(5)
C4A	C12A	O4A	Eu1[3]	-103.2(10)	C12B	C4B	C5B	C6B	-4.4(9)
C4B	C5B	C6B	C7B	-178.0(6)	C12B	C4B	C5B	C10B	175.6(5)
C4B	C5B	C10B	C9B	179.6(5)	C12C	C4C	C5C	C6C	7.3(9)

C4B	C5B	C10B	N1B	0.1(8)	C12C	C4C	C5C	C10C	-172.8(5)
C4C	C5C	C6C	C7C	-178.6(6)	N1A	C2A	C3A	C4A	0.9(9)
C4C	C5C	C10C	C9C	179.8(5)	N1A	C2A	C11A	O1A	5.7(8)
C4C	C5C	C10C	N1C	1.0(8)	N1A	C2A	C11A	O2A	-176.7(6)
C4C	C12C	O3C	Eu2[2]	156.5(5)	N1B	C2B	C3B	C4B	-0.2(9)
C4C	C12C	O4C	Eu2[4]	83.8(18)	N1B	C2B	C11B	O1B	8.8(8)
C5A	C4A	C12A	O3A	152.8(5)	N1B	C2B	C11B	O2B	-169.9(5)
C5A	C4A	C12A	O4A	-29.4(8)	N1C	C2C	C3C	C4C	2.6(9)
C5A	C6A	C7A	C8A	-0.2(10)	N1C	C2C	C11C	Eu2	-8.2(5)
C5A	C10A	N1A	Eu1	-166.3(4)	N1C	C2C	C11C	O1C	-17.5(8)
C5A	C10A	N1A	C2A	1.2(8)	N1C	C2C	C11C	O2C	163.2(6)
C5B	C4B	C12B	O3B	122.5(6)	O1B	C11B	O2B	Eu2	-24.1(8)
C5B	C4B	C12B	O4B	-58.0(7)	O2A	C11A	O1A	Eu1	-173.0(5)
C5B	C6B	C7B	C8B	-2.3(10)	O2B	C11B	O1B	Eu1[1]	176.9(4)
C5B	C10B	N1B	Eu1[1]	-168.2(4)	O2C	C11C	O1C	Eu2	-164.1(5)
C5B	C10B	N1B	C2B	3.0(8)	O3A	C12A	O4A	Eu1[3]	74.2(11)
C5C	C4C	C12C	O3C	-145.6(6)	O3C	C12C	O4C	Eu2[4]	-92.8(17)
C5C	C4C	C12C	O4C	37.5(8)	O4A	C12A	O3A	Eu1[2]	31.5(10)
C5C	C6C	C7C	C8C	-0.7(10)	O4C	C12C	O3C	Eu2[2]	-26.8(11)
C5C	C10C	N1C	Eu2	165.4(4)					

Symmetry codes: [1]2-x,1-y,1-z; [2]-1+x,+y,+z; [3]1-x,-y,1-z; [4]1-x,1-y,2-z.

Table S15. Torsion Angles for (9) $[Tb_2(Qdca)_3(H_2O)_4]\cdot H_2O$.

A	B	C	D	Angle/°	A	B	C	D	Angle/°
C2A	C3A	C4A	C5A	3.2(8)	C5C	C10C	N1C	Tb2	164.3(4)
C2A	C3A	C4A	C12A	-172.3(5)	C6A	C5A	C10A	C9A	3.0(8)
C2A	C11A	O1A	Tb1	2.5(8)	C6A	C5A	C10A	N1A	-178.8(5)
C2B	C3B	C4B	C5B	3.6(8)	C6A	C7A	C8A	C9A	3.1(10)
C2B	C3B	C4B	C12B	-175.6(5)	C6B	C5B	C10B	C9B	0.1(9)
C2B	C11B	O1B	Tb1[1]	-2.6(8)	C6B	C5B	C10B	N1B	-179.9(5)
C2B	C11B	O2B	Tb2	153.8(4)	C6B	C7B	C8B	C9B	0.4(12)
C2C	C3C	C4C	C5C	-5.4(9)	C6C	C5C	C10C	C9C	-1.3(8)
C2C	C3C	C4C	C12C	171.2(5)	C6C	C5C	C10C	N1C	-179.0(5)
C2C	C11C	O1C	Tb2	17.8(8)	C6C	C7C	C8C	C9C	0.0(11)
C3A	C2A	C11A	O1A	-171.6(5)	C7A	C8A	C9A	C10A	-1.9(10)
C3A	C2A	C11A	O2A	5.7(9)	C7B	C8B	C9B	C10B	0.6(11)
C3A	C2A	N1A	C10A	-1.3(8)	C7C	C8C	C9C	C10C	1.8(11)
C3A	C2A	N1A	Tb1	168.4(4)	C8A	C9A	C10A	C5A	-1.1(9)
C3A	C4A	C5A	C6A	176.9(6)	C8A	C9A	C10A	N1A	-179.3(6)
C3A	C4A	C5A	C10A	-3.5(8)	C8B	C9B	C10B	C5B	-0.9(10)
C3A	C4A	C12A	O3A	-32.3(7)	C8B	C9B	C10B	N1B	179.2(6)
C3A	C4A	C12A	O4A	146.3(6)	C8C	C9C	C10C	C5C	-1.1(9)
C3B	C2B	C11B	O1B	-170.3(5)	C8C	C9C	C10C	N1C	176.7(6)
C3B	C2B	C11B	O2B	10.3(8)	C9A	C10A	N1A	C2A	179.0(5)
C3B	C2B	N1B	C10B	-3.7(8)	C9A	C10A	N1A	Tb1	12.2(7)
C3B	C2B	N1B	Tb1[1]	169.3(5)	C9B	C10B	N1B	C2B	-176.4(6)
C3B	C4B	C5B	C6B	176.3(6)	C9B	C10B	N1B	Tb1[1]	11.9(8)
C3B	C4B	C5B	C10B	-3.5(8)	C9C	C10C	N1C	C2C	177.5(5)
C3B	C4B	C12B	O3B	-59.3(8)	C9C	C10C	N1C	Tb2	-13.4(8)
C3B	C4B	C12B	O4B	119.6(6)	C10A	C5A	C6A	C7A	-2.0(9)
C3C	C2C	C11C	O1C	161.2(6)	C10B	C5B	C6B	C7B	0.9(9)
C3C	C2C	C11C	O2C	-18.2(9)	C10C	C5C	C6C	C7C	3.1(9)
C3C	C2C	N1C	C10C	2.4(8)	C11A	C2A	C3A	C4A	177.4(5)
C3C	C2C	N1C	Tb2	-168.7(5)	C11A	C2A	N1A	C10A	-179.5(5)

C3C	C4C	C5C	C6C	-175.7(6)	C11A	C2A	N1A	Tb1	-9.8(6)
C3C	C4C	C5C	C10C	3.0(8)	C11B	C2B	C3B	C4B	179.3(5)
C3C	C4C	C12C	O3C	37.8(8)	C11B	C2B	N1B	C10B	177.1(5)
C3C	C4C	C12C	O4C	-139.1(6)	C11B	C2B	N1B	Tb1[1]	-9.9(6)
C4A	C5A	C6A	C7A	177.6(6)	C11C	C2C	C3C	C4C	-176.8(6)
C4A	C5A	C10A	C9A	-176.6(5)	C11C	C2C	N1C	C10C	-178.1(5)
C4A	C5A	C10A	N1A	1.6(8)	C11C	C2C	N1C	Tb2	10.8(6)
C4A	C12A	O3A	Tb1[2]	-151.4(4)	C12A	C4A	C5A	C6A	-8.0(9)
C4A	C12A	O4A	Tb1[3]	-102.8(10)	C12A	C4A	C5A	C10A	171.6(5)
C4B	C5B	C6B	C7B	-178.9(7)	C12B	C4B	C5B	C6B	-4.6(9)
C4B	C5B	C10B	C9B	179.9(6)	C12B	C4B	C5B	C10B	175.7(5)
C4B	C5B	C10B	N1B	-0.2(9)	C12C	C4C	C5C	C6C	7.9(9)
C4C	C5C	C6C	C7C	-178.2(6)	C12C	C4C	C5C	C10C	-173.3(5)
C4C	C5C	C10C	C9C	179.9(5)	N1A	C2A	C3A	C4A	-0.7(9)
C4C	C5C	C10C	N1C	2.2(8)	N1A	C2A	C11A	O1A	6.7(8)
C4C	C12C	O3C	Tb2[2]	155.4(5)	N1A	C2A	C11A	O2A	-176.0(6)
C4C	C12C	O4C	Tb2[4]	82(2)	N1B	C2B	C3B	C4B	0.2(9)
C5A	C4A	C12A	O3A	152.4(5)	N1B	C2B	C11B	O1B	8.9(8)
C5A	C4A	C12A	O4A	-29.0(8)	N1B	C2B	C11B	O2B	-170.5(6)
C5A	C6A	C7A	C8A	-1.1(10)	N1C	C2C	C3C	C4C	2.8(9)
C5A	C10A	N1A	C2A	0.9(8)	N1C	C2C	C11C	O1C	-18.3(8)
C5A	C10A	N1A	Tb1	-165.9(4)	N1C	C2C	C11C	O2C	162.3(6)
C5B	C4B	C12B	O3B	121.5(7)	O1B	C11B	O2B	Tb2	-25.6(9)
C5B	C4B	C12B	O4B	-59.5(7)	O2A	C11A	O1A	Tb1	-174.6(5)
C5B	C6B	C7B	C8B	-1.2(11)	O2B	C11B	O1B	Tb1[1]	176.8(4)
C5B	C10B	N1B	C2B	3.7(8)	O2C	C11C	O1C	Tb2	-162.8(5)
C5B	C10B	N1B	Tb1[1]	-168.1(4)	O3A	C12A	O4A	Tb1[3]	75.7(12)
C5C	C4C	C12C	O3C	-145.7(6)	O3C	C12C	O4C	Tb2[4]	-95(2)
C5C	C4C	C12C	O4C	37.3(8)	O4A	C12A	O3A	Tb1[2]	30.1(10)
C5C	C6C	C7C	C8C	-2.4(10)	O4C	C12C	O3C	Tb2[2]	-27.9(11)
C5C	C10C	N1C	C2C	-4.8(8)					

Symmetry codes: [1]2-x,1-y,1-z; [2]-1+x,+y,+z; [3]1-x,-y,1-z; [4]1-x,1-y,2-z.

Table S16. Torsion Angles for (13) $[Er_2(Qdca)_3(H_2O)_4]\cdot 4H_2O$.

A	B	C	D	Angle/°	A	B	C	D	Angle/°
C2A	C3A	C4A	C5A	-0.9(6)	C5C	C10C	N1C	C2C	1.4(5)
C2A	C3A	C4A	C12A	177.5(3)	C6A	C5A	C10A	C9A	-2.9(6)
C2A	C11A	O1A	Er1	-9.1(6)	C6A	C5A	C10A	N1A	178.0(4)
C2B	C3B	C4B	C5B	-4.0(5)	C6A	C7A	C8A	C9A	-3.6(9)
C2B	C3B	C4B	C12B	175.2(3)	C6B	C5B	C10B	C9B	2.7(6)
C2B	C11B	O1B	Er1[1]	-2.3(5)	C6B	C5B	C10B	N1B	-177.9(4)
C2B	C11B	O2B	Er2	-156.0(2)	C6B	C7B	C8B	C9B	1.7(8)
C2C	C3C	C4C	C5C	1.2(6)	C6C	C5C	C10C	C9C	1.7(6)
C2C	C3C	C4C	C12C	-176.7(3)	C6C	C5C	C10C	N1C	179.9(4)
C2C	C11C	O1C	Er2	-4.4(5)	C6C	C7C	C8C	C9C	2.3(8)
C3A	C2A	C11A	O1A	170.7(4)	C7A	C8A	C9A	C10A	2.5(8)
C3A	C2A	C11A	O2A	-7.7(6)	C7B	C8B	C9B	C10B	-1.1(7)
C3A	C2A	N1A	C10A	2.0(5)	C7C	C8C	C9C	C10C	-2.7(7)
C3A	C4A	C5A	C6A	-177.0(4)	C8A	C9A	C10A	C5A	0.7(7)
C3A	C4A	C5A	C10A	1.8(5)	C8A	C9A	C10A	N1A	179.8(4)
C3A	C4A	C12A	O3A	44.8(5)	C8B	C9B	C10B	C5B	-1.1(6)
C3A	C4A	C12A	O4A	-134.2(4)	C8B	C9B	C10B	N1B	179.5(4)
C3B	C2B	C11B	O1B	169.3(3)	C8C	C9C	C10C	C5C	0.7(6)
C3B	C2B	C11B	O2B	-11.5(5)	C8C	C9C	C10C	N1C	-177.6(4)

C3B	C2B	N1B	Er1[1]	-164.6(3)	C9A	C10A	N1A	C2A	179.9(4)
C3B	C2B	N1B	C10B	1.7(5)	C9B	C10B	N1B	Er1[1]	-20.5(5)
C3B	C4B	C5B	C6B	-178.1(4)	C9B	C10B	N1B	C2B	175.6(4)
C3B	C4B	C5B	C10B	1.9(5)	C9C	C10C	N1C	Er2	4.1(5)
C3B	C4B	C12B	O3B	68.6(5)	C9C	C10C	N1C	C2C	179.6(3)
C3B	C4B	C12B	O4B	-110.8(4)	C10A	C5A	C6A	C7A	1.9(6)
C3C	C2C	C11C	O1C	-171.8(4)	C10B	C5B	C6B	C7B	-2.2(6)
C3C	C2C	C11C	O2C	8.2(6)	C10C	C5C	C6C	C7C	-2.1(6)
C3C	C2C	N1C	Er2	172.5(3)	C11A	C2A	C3A	C4A	-179.4(3)
C3C	C2C	N1C	C10C	-3.7(5)	C11A	C2A	N1A	C10A	-179.7(3)
C3C	C4C	C5C	C6C	179.0(4)	C11B	C2B	C3B	C4B	-177.1(3)
C3C	C4C	C5C	C10C	-3.3(5)	C11B	C2B	N1B	Er1[1]	14.8(4)
C3C	C4C	C12C	O3C	79.4(5)	C11B	C2B	N1B	C10B	-178.9(3)
C3C	C4C	C12C	O4C	-97.3(5)	C11C	C2C	C3C	C4C	-178.0(4)
C4A	C5A	C6A	C7A	-179.3(4)	C11C	C2C	N1C	Er2	-7.1(4)
C4A	C5A	C10A	C9A	178.2(4)	C11C	C2C	N1C	C10C	176.7(3)
C4A	C5A	C10A	N1A	-0.9(5)	C12A	C4A	C5A	C6A	4.7(6)
C4A	C12A	O3A	Er1[2]	155.8(3)	C12A	C4A	C5A	C10A	-176.5(3)
C4A	C12A	O4A	Er1[3]	112.8(13)	C12B	C4B	C5B	C6B	2.7(6)
C4B	C5B	C6B	C7B	177.8(4)	C12B	C4B	C5B	C10B	-177.3(3)
C4B	C5B	C10B	C9B	-177.3(4)	C12C	C4C	C5C	C6C	-3.2(6)
C4B	C5B	C10B	N1B	2.1(6)	C12C	C4C	C5C	C10C	174.6(3)
C4B	C12B	O4B	Er1	-100.1(8)	N1A	C2A	C3A	C4A	-1.1(6)
C4C	C5C	C6C	C7C	175.7(4)	N1A	C2A	C11A	O1A	-7.8(5)
C4C	C5C	C10C	C9C	-176.1(4)	N1A	C2A	C11A	O2A	173.9(4)
C4C	C5C	C10C	N1C	2.1(5)	N1B	C2B	C3B	C4B	2.3(6)
C4C	C12C	O3C	Er2[4]	88.6(4)	N1B	C2B	C11B	O1B	-10.1(5)
C4C	C12C	O4C	Er2[2]	162.9(3)	N1B	C2B	C11B	O2B	169.1(3)
C5A	C4A	C12A	O3A	-136.9(4)	N1C	C2C	C3C	C4C	2.5(6)
C5A	C4A	C12A	O4A	44.1(5)	N1C	C2C	C11C	O1C	7.8(5)
C5A	C6A	C7A	C8A	1.3(8)	N1C	C2C	C11C	O2C	-172.2(4)
C5A	C10A	N1A	C2A	-1.0(5)	O1B	C11B	O2B	Er2	23.1(5)
C5B	C4B	C12B	O3B	-112.2(5)	O2A	C11A	O1A	Er1	169.2(3)
C5B	C4B	C12B	O4B	68.4(5)	O2B	C11B	O1B	Er1[1]	178.6(3)
C5B	C6B	C7B	C8B	0.0(7)	O2C	C11C	O1C	Er2	175.7(3)
C5B	C10B	N1B	Er1[1]	160.1(3)	O3A	C12A	O4A	Er1[3]	-66.1(15)
C5B	C10B	N1B	C2B	-3.9(5)	O3B	C12B	O4B	Er1	80.5(9)
C5C	C4C	C12C	O3C	-98.4(4)	O3C	C12C	O4C	Er2[2]	-13.5(7)
C5C	C4C	C12C	O4C	84.8(5)	O4A	C12A	O3A	Er1[2]	-25.3(7)
C5C	C6C	C7C	C8C	0.1(7)	O4C	C12C	O3C	Er2[4]	-94.9(5)
C5C	C10C	N1C	Er2	-174.1(3)					

Symmetry codes: [1]-x,1-y,1-z; [2]1+x,+y,+z; [3]1-x,-y,1-z; [4]1-x,1-y,2-z.

Table S17. Hydrogen bonds of the **(1)** [$Nd_2(Qdca)_3(H_2O)_3$].

D-H..A	d(D-H) /Å	d(H...A) /Å	d(D...A) /Å	<DHA/°
O1W-H1WB...O2C[1]	0.84	1.85	2.666	163.93
O2W-H2WA...O4C	0.84	1.96	2.778	163.93
O2W-H2WB...N1B[2]	0.84	1.92	2.746	168.50
O3W-H3WA...O1A[2]	0.84	2.60	3.364	152.90
O3W-H3WB...N1B[3]	0.84	2.50	3.053	123.92
O1W-H1WA...O1A	0.84	2.37	3.032	135.84
O1W-H1WA...O3A[4]	0.84	2.37	2.800	112.85

Symmetry codes: [1] x-1/2, -y+3/2, z; [2] x+1/2, -y+3/2, z; [3] x+1/2, -y+3/2, z+1; [4]-x+1, -y+1, z+1/2.

Table S18. Hydrogen bonds of the **(4)** [$Nd_2(Qdca)_3(H_2O)_4$]·$3H_2O$.

D-H..A	d(D-H) /Å	d(H...A) /Å	d(D...A) /Å	<DHA/°
O3W-H3WA... O3A[1]	0.84	1.85	2.670	164.86
O2W-H2WA... O2C[2]	0.84	2.06	2.815	149.73
O2W-H2WB... O6W	0.84	2.26	2.949	139.76
O1W-H1WA... O7W	0.84	1.95	2.699	148.15
O4W-H4WA... O2C[2]	0.84	2.02	2.778	149.21
O5W-H5WA... O3A[3]	0.84	1.92	2.695	153.77
O5W-H5WB... O6W[4]	0.84	2.19	2.852	135.27
O7W-H7WA... O5W	0.84	1.92	2.709	156.50
O7W-H7WB... O1C[5]	0.84	2.02	2.779	150.25
O6W-H6WA... O1B[6]	0.84	1.91	2.733	166.27
O6W-H6WB... O3C[2]	0.84	2.10	2.867	151.44

Symmetry codes: [1] x-1, y-1, z; [2] x+1, y, z; [3]-x+1, -y+2, -z; [4]-x+1, -y+1, -z; [5]-x, -y+2, -z; [6]-x+1, -y+1, -z+1.

Table S19. Hydrogen bonds of the **(8)** [$Eu_2(Qdca)_3(H_2O)_4$]·H_2O.

D-H..A	d(D-H) /Å	d(H...A) /Å	d(D...A) /Å	<DHA/°
O2W-H2WA... O1B	0.84	2.10	2.799	139.98
O2W-H2WB...O2A[1]	0.84	1.87	2.698	166.67
O3W-H3WA...O3B[2]	0.84	1.98	2.756	153.43
O4W-H4WA...O5W[3]	0.84	2.01	2.757	147.26
O4W-H4WB...O3B[4]	0.84	2.18	2.989	160.94
O5W-H5WA...O1C[5]	0.84	2.81	3.407	129.21
O5W-H5WA...O2C[5]	0.84	1.91	2.741	169.08
O5W-H5WB...O1A[3]	0.84	2.27	2.936	136.20
O5W-H5WB...O2A[3]	0.84	2.70	3.490	156.66

Symmetry codes: [1] -x+1, -y+1, -z+1; [2] -x+2, -y+1, -z+2; [3] -x+2, -y+1, -z+1; [4] -x+2, -y, -z+1; [5]-x+2, -y+2, -z+2.

Table S20. Hydrogen bonds of the **(9)** [$Tb_2(Qdca)_3(H_2O)_4$]·H_2O.

D-H..A	d(D-H) /Å	d(H...A) /Å	d(D...A) /Å	<DHA/°
O1W-H1WA...O1B	0.84	2.09	2.800	142.55
O1W-H1WB...O2A[1]	0.84	1.90	2.711	163.27
O2W-H2WB...O3B[2]	0.84	1.93	2.758	169.96
O3W-H3WA...O5W[3]	0.84	1.92	2.710	156.72
O4W-H4WA...O3B[4]	0.84	2.21	3.013	159.21
O4W-H4WB...O5W[5]	0.84	1.94	2.761	167.17
O5W-H5WA...O1A[6]	0.84	2.51	2.942	113.38
O5W-H5WB...O2C	0.84	1.89	2.726	175.54

Symmetry codes: [1] -x+1, -y+1, -z+1; [2] -x+2, -y+1, -z+2; [3] -x+2, -y+2, -z+2; [4] -x+2, -y, -z+1; [5]x, y-1, z-1, -z+1; [6]x, y+1, z+1.

Table S21. Hydrogen bonds of the **(13)** [$Er_2(Qdca)_3(H_2O)_4$]·$4H_2O$.

D-H..A	d(D-H) /Å	d(H...A) /Å	d(D...A) /Å	<DHA/°
O1W-H1WA...O3B[1]	0.84	2.08	2.904	165.59
O1W-H1WB...O6W[2]	0.84	1.96	2.741	154.95
O2W-H2WA...O3C[3]	0.84	1.98	2.761	154.28
O2W-H2WB...O5W	0.84	1.94	2.681	147.00
O3W-H3WA...O1B	0.84	2.27	2.895	131.92
O3W-H3WB...O2A[4]	0.84	1.87	2.708	171.34
O4W-H4WA...O6W	0.84	1.84	2.673	171.96
O4W-H4WB...O8W	0.84	1.96	2.656	140.33
O5W-H5WB...O3B[5]	0.84	1.89	2.700	160.76

O6W-H6WA...O7W	0.84	1.88	2.688	160.16
O6W-H6WB...O1A[2]	0.84	2.34	2.810	116.04
O6W-H6WB...O2A[2]	0.84	2.54	3.261	145.06
O7W-H7WA...O2C[3]	0.84	1.95	2.728	152.70
O7W-H7WB...O2C[6]	0.84	1.96	2.793	175.13
O8W-H8WB...O7W[6]	0.84	1.95	2.779	169.31
O8W-H8WA...O5W[6]	0.84	2.16	2.788	131.06

Symmetry codes: [1] -x, -y, -z+1; [2] -x, -y+1, -z+1; [3] x-1, y, z; [4] -x+1, -y+1, -z+1; [5] -x, -y+1, -z+2; [6] -x, -y+2, -z+2.

Table S22. Conformation of the COO^- groups of the quionoline-2,4-dicarboxylate ligand.

Position of COO^-	A-Qdca		B-Qdca		C-Qdca	
	**(1)** *[$Nd_2(Qdca)_3(H_2O)_3$]*					
2	C3A-C2A-C11A-O1A	166.8(8)	C3B-C2B-C11B-O1B	174.3(6)	C3C-C2C-C11C-O1C	-173.5(6)
	C3A-C2A-C11A-O2A	-12.1(13)	C3B-C2B-C11B-O2B	-5.4(9)	C3C-C2C-C11C-O2C	7.0(10)
4	C3A-C4A-C12A-O3A	88.4(10)	C3B-C4B-C12B-O3B	-61.5(8)	C3C-C4C-C12C-O3C	-152.6(8)
	C3A-C4A-C12A-O4A	-93.1(10)	C3B-C4B-C12B-O4B	120.1(7)	C3C-C4C-C12C-O4C	24.4(10)
	**(4)** *[$Nd_2(Qdca)_3(H_2O)_4$]·$3H_2O$*					
	**A-Qdca**		**B-Qdca**		**C-Qdca**	
2	C3A-C2A-C11A-O1A	-154.2(6)	C3B-C2B-C11B-O1B	171.2(6)	C3C-C2C-C11C-O1C	151.4(7)
	C3A-C2A-C11A-O2A	25.6(9)	C3B-C2B-C11B-O2B	-7.0(10)	C3C-C2C-C11C-O2C	-28.7(10)
4	C3A-C4A-C12A-O3A	-32.0(10)	C3B-C4B-C12B-O3B	118.2(7)	C3C-C4C-C12C-O3C	56.6(11)
	C3A-C4A-C12A-O4A	142.7(7)	C3B-C4B-C12B-O4B	61.2(9)	C3C-C4C-C12C-O4C	-123.6(8)
	**(8)** *$Eu_2(Qdca)_3(H_2O)_4$·H_2O*					
	**A-Qdca**		**B-Qdca**		**C-Qdca**	
2	C3A-C2A-C11A-O1A	-172.0(5)	C3B-C2B-C11B-O1B	-171.0(5)	C3C-C2C-C11C-O1C	162.1(5)
	C3A-C2A-C11A-O2A	5.5(9)	C3B-C2B-C11B-O2B	10.2(8)	C3C-C2C-C11C-O2C	-17.2(8)
4	C3A-C4A-C12A-O3A	-32.1(7)	C3B-C4B-C12B-O3B	-58.7(8)	C3C-C4C-C12C-O3C	37.7(8)
	C3A-C4A-C12A-O4A	145.6(5)	C3B-C4B-C12B-O4B	120.7(6)	C3C-C4C-C12C-O4C	-139.2(6)
	**(13)** *[$Er_2(Qdca)_3(H_2O)_4$]·$4H_2O$*					
	**A-Qdca**		**B-Qdca**		**C-Qdca**	
2	C3A-C2A-C11A-O1A	170.7(4)	C3B-C2B-C11B-O1B	169.3(3)	C3C-C2C-C11C-O1C	-171.8(4)
	C3A-C2A-C11A-O2A	-7.7(6)	C3B-C2B-C11B-O2B	-11.5(5)	C3C-C2C-C11C-O2C	8.2(6)
4	C3A-C4A-C12A-O3A	44.8(5)	C3B-C4B-C12B-O3B	68.6(5)	C3C-C4C-C12C-O3C	79.4(5)
	C3A-C4A-C12A-O4A	-134.2(4)	C3B-C4B-C12B-O4B	-110.8(4)	C3C-C4C-C12C-O4C	-97.3(5)

Table S23. Dihedral angles between best planes of carboxylic groups and quinolone ring.

Position of COO^-	A-Qdca		B-Qdca		C-Qdca	
	**(1)** *[$Nd_2(Qdca)_3(H_2O)_3$]*					
2	atoms: C4A C3A C2A N1A C10A C5A / atoms:O1A C11A O2A	12.90°	atoms:C3B C4B C5B C10B N1B C2B / atoms: O2B C11B O1B	6.11°	atoms: C2C C3C C4C C5C C10C N1C / atoms: O1C C11C O2C	6.96°
4	atoms:C4A C3A C2A N1A C10A C5A / atoms:O4A C12A O3A	88.37°	atoms: C3B C4B C5B C10B N1B C2B / atoms: O3B C12B O4B	62.61°	atoms: C2C C3C C4C C5C C10C N1C / atoms: O4C C12C O3C	24.96°
	**(4)** *[$Nd_2(Qdca)_3(H_2O)_4$]·$3H_2O$*					
	**A-Qdca**		**B-Qdca**		**C-Qdca**	
2	atoms: C2A N1A C10A C5A C4A C3A / atoms: O1A C11A O2A	25.40°	atoms: C10B N1B C2B C3B C4B C5B / atoms: O2B C11B O1B	8.82°	atoms:C10C N1C C2C C3C C4C C5C / atoms: O2C C11C O1C	28.88°
4	atoms: C2A N1A C10A C5A C4A C3A / atoms: O4A C12A O3A	35.88°	atoms: C10B N1B C2B C3B C4B C5B / atoms:O3B	61.65°	atoms:C10C N1C C2C C3C C4C C5C / atoms:O4C C12C O3C	51.93°
	**(8)** *$Eu_2(Qdca)_3(H_2O)_4$]·H_2O*					
	**A-Qdca**		**B-Qdca**		**C-Qdca**	

2	atoms: C2A C3A C4A C5A C10A N1A<br>atoms:O2A C11A O1A	6.70°	atoms:C4B C5B C10B N1B C2B C3B<br>atoms: O2B C11B O1B	10.64°	atoms:C4C C5C C10C N1C C2C C3C<br>atoms:O1C C11C O2C	18.58°
4	atoms: C2A C3A C4A C5A C10A N1A<br>atoms: O3A C12A O4A	32.20°	atoms: C4B C5B C10B N1B C2B C3B<br>atoms:O4B C12B O3B	58.63°	atoms: C4C C5C C10C N1C C2C C3C<br>atoms:O4C C12C O3C	39.50°
			(**13**) *[$Er_2(Qdca)_3(H_2O)_4$]·4H_2O*			
	**A-Qdca**		**B-Qdca**		**C-Qdca**	
2	atoms:N1A C2A C3A C4A C5A C10A<br>atoms:O2A C11A O1A	8.20°	atoms:C4B C5B C10B N1B C2B C3B<br>atoms:O1B C11B O2B	11.70°	atoms:N1C C10C C5C C4C C3C C2C<br>atoms:O1C C11C O2C	8.74°
4	atoms:N1A C2A C3A C4A C5A C10A<br>atoms:O3A C12A O4A	44.51°	atoms:C4B C5B C10B N1B C2B C3B<br>atoms:O4B C12B O3B	69.12°	atoms:N1C C10C C5C C4C C3C C2C<br>atoms:O3C C12C O4C	82.27°

Table S24. Data of thermogravimetric analysis.

**Neodymium complexes**	**ΔT_1 (°C)**	**Mass loss (%)**	**ΔT_2 (°C)**	**Mass loss (%)**	**ΔT_3 (°C)**	**Mass loss (%)**	**ΔT_4 (°C)**	**Mass loss (%)**	**ΔT_5 (°C)**	**Mass loss (%)**
**100°C**	30-135	2.38	135-357	7.49	357-527	50.53	527-592	4.19	592-697	2.69
**120°C**	30-122	3.91	122-345	8.33	345-522	48.81	522-587	4.40	587-680	3.12
**150°C**	30-304	10.54	-	-	304-487	42.75	487-591	11.23	591-675	3.83
**Europium complexes**	**ΔT_1 (°C)**	**Mass loss (%)**	**ΔT_2 (°C)**	**Mass loss (%)**	**ΔT_3 (°C)**	**Mass loss (%)**	**ΔT_4 (°C)**	**Mass loss (%)**	**ΔT_5 (°C)**	**Mass loss (%)**
**100°C**	30-121<br>121-165*	4.67<br>2.17*	165-326	6.02	326-443	30.69	443-516	49.25	516-628	5.81
**120°C**	30-143	3.86	143-294	6.85	294-430	25.83	430-516	23.86	516-667	5.81
**150°C**	30-153	7.07	153-285	4.28	285-443	37.42	443-534	14.52	534-638	3.98
**Terbium complexes**	**ΔT_1 (°C)**	**Mass loss (%)**	**ΔT_2 (°C)**	**Mass loss (%)**	**ΔT_3 (°C)**	**Mass loss (%)**	**ΔT_4 (°C)**	**Mass loss (%)**	**ΔT_5 (°C)**	**Mass loss (%)**
**100°C**	30-122	3.63	122-325	8.06	325-704	53.08	-	-	-	-
**120°C**	30-132	4.43	132-314	6.97	314-689	53.73	-	-	-	-
**150°C**	30-323	10.47	323-712	52.00	-	-	-	-	-	-
**Erbium complexes**	**ΔT_1 (°C)**	**Mass loss (%)**	**ΔT_2 (°C)**	**Mass loss (%)**	**ΔT_3 (°C)**	**Mass loss (%)**	**ΔT_4 (°C)**	**Mass loss (%)**	**ΔT_5 (°C)**	**Mass loss (%)**
**100°C**	30-114	4.76	114-190*<br>190-330	3.58*<br>5.09	330-570	50.57	-	-	570-720	2.00
**120°C**	30-144	9.69	144-192*<br>192-303	2.25*<br>2.72	303-544	48.32	-	-	544-723	2.90
**150°C**	30-326*	9.52	-	-	326-573	46.10	-	-	573-741	3.85

Table S25. The main crystallographic data for *[$Gd_2(Qdca)_3(H_2O)_4$]•H_2O*.

**Compound**	**[$Gd_2(Qdca)_3(H_2O)_4$]·H_2O**
Empirical formula	$C_{33}H_{25}N_3O_{17}Gd_2$
Formula weight	1049.5
T /K	295(2)
Crystal system	*Triclinic*
Space group	*P*-1
a /Å	9.8314(1)
b /Å	12.3257(2)
c /Å	14.1759(1)
α /°	89.643(9)
β /°	82.577(1)
γ /°	86.612(1)
Volume /Å^3	1700.44(1)
